# Stereoselective Control of the Cu Activation of β,β-Diboryl Acrylates for Allylic Coupling Protocols with Concomitant Lactonization

**DOI:** 10.1021/acs.orglett.3c03640

**Published:** 2023-12-15

**Authors:** Mireia Pujol, María Méndez, Elena Fernández

**Affiliations:** † Faculty of Chemistry, University Rovira i Virgili, 43007 Tarragona, Spain; ‡ Sanofi R&D, Integrated Drug Discovery, Industriepark Höchst, Building G838, 65926 Frankfurt am Main, Germany

## Abstract

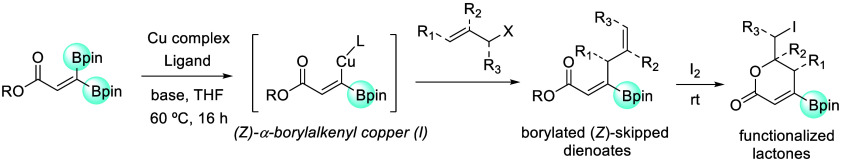

The key to a successful C–B activation is to discriminate between two geminal boryl moieties that are exposed to the same reaction conditions. Here we describe a stereoselective C–B activation of β,β-diboryl acrylates forming exclusively the (*Z*)-α-borylalkenyl copper(I) key intermediate, for subsequent allylic alkylation reactions. The new borylated (*Z*)-skipped dienoates followed a feasible iodo-lactonization sequence for the preparation of borylated lactone cores, which can be used in drug discovery.

Boron-selective chemical transformations allow the modular and rapid construction of molecular diversity and complexity for applications in organic synthesis for biomedical purposes.^1^ The two installed geminal pinacolboryl substituents on 1,1-diborylalkenes^2−6^ can be stereoselectively differentiated and transformed in a stepwise manner, showing the potential of 1,1-diborylalkenes as versatile intermediates in organic synthesis.^7−12^ The inclusion of carbonyl groups in β,β-diboryl acrylates contributes to the increase in the functional diversity through the C–B discrimination pathway because Suzuki–Miyaura coupling with arylhalides, in the presence of Pd(OAc)_2_ and DtBPF [DtBPF = 1,1′-bis(di-*tert*-butylphosphino)ferrocene], occurred selectively at the boron site *trans* to the ester groups (Scheme 1a).^13^ However, here we present a complementary stereoselective C–B activation of β,β-diboryl acrylates with Cu(I) salts that exclusively activates the boron site *cis* to the ester group, promoting nucleophilic C–C bond formation at the more hindered C(sp^2^)–B position through allylic coupling reactions (Scheme 1b).

**Scheme 1:**
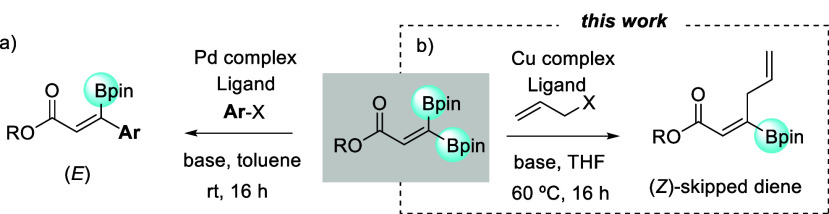
Complementary Stereoselective C–B Activation of β,β-Diborylacrylates in Pd-Catalyzed Cross-Coupling or Cu-Catalyzed Allylic Alkylation Reactions

The access to (*Z*)- or (*E*)-3-aryl 3-pinacolboryl acrylate compounds has been achieved via *syn*(14,15) or *anti*(16) catalytic hydroboration of alkynoates, respectively. However, substituents other than aryl groups at C_β_ have been less explored, particularly those that include allylic substituents, due to the inherent competition with respect to the hydroboration pathway. In that context, the synthesis of borylated (*Z*)-skipped dienoates is performed for the first time in this work (Scheme 2), taking advantage of the exclusive formation of the (*Z*)-stereoisomer for subsequent iodo-lactonization to prepare a series of functionalized lactones, with potential interest in medicinal chemistry. In particular, we focused on the preparation of α,β-unsaturated δ-lactone moieties for straightforward access to the essential core of novel kazusamycin A derivatives, for use as potent antitumor agents.^17^

**Scheme 2:**
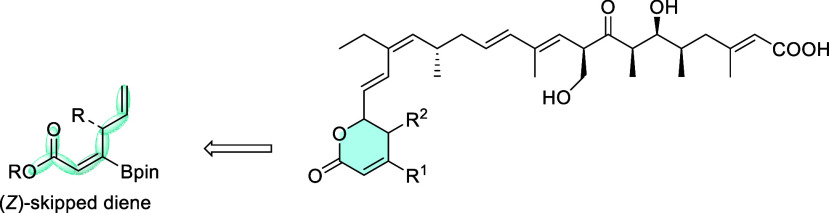
Structural Design of Kazusamycin A Derivatives

In our initial experiments, we investigated the allylic coupling reaction of ethyl 3,3′-bispinacolboryl propenoate (**1a**) with 3-bromoprop-1-ene by employing a catalytic amount of copper salt CuCl and ligand PPh_3_ in the presence of a variety of bases, in THF. We initially chose LiO^*t*^Bu on the basis of its efficiency in the Cu-catalyzed site-selective activation of 1,1-diborylalkenes, containing aryl or vinyl substituents.^11^ However, only ethyl (*E*)-3-(4,4,5,5-tetramethyl-1,3,2-dioxaborolan-2-yl)acrylate (**3**a) could be identified in the reaction mixture as a consequence of the selective activation on the boron site *cis* to the ester group, followed by protonation (Table 1, entry 1). A similar reaction outcome was observed when the *i*PrCuCl complex was used, in the presence of LiO^*t*^Bu as the base (Table 1, entry 2). The lack of allylic coupling moved us to consider alternative bases, avoiding the alkoxide groups that might be responsible for B activation through boron“ate” intermediates followed by a protonation step. When the base involved was Cs_2_CO_3_, we identified a moderate yield of the desired product ethyl (*Z*)-3-pinacolboryl-2,5-hexadienoate **2a**, with a small amount of protodeborated byproduct **3a** (Table 1, entry 3). Replacing Cs_2_CO_3_ with K_2_CO_3_ favored the exclusive formation of coupled product **2a**, although in a moderate yield (Table 1, entry 4). The highest yield with exclusive formation of product **2a** was achieved when K_3_PO_4_ was the base used, at 60 °C, because lower temperatures decreased the yield (Table 1, entries 5 and 6).

**Table 1:**
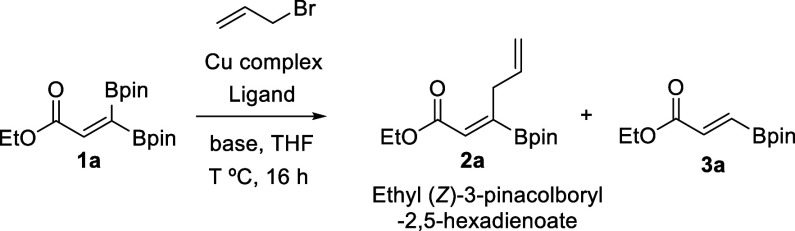
Optimization of the Reaction Conditions for Stereoselective Cu Activation of β,β-Diborylacrylate **1a** toward Nucleophilic Allylic Coupling

entry^a^	Cu(I)/ligand	base	*T* (°C)	NMR yield^b^ (%)	**2a**:**3a**	**2a** (*Z*:*E*)
1	CuCl/PPh_3_	LiO^*t*^Bu	60	>90	1:99	–
2	iPrCuCl	LiO^*t*^Bu	60	>90	1:99	–
3	CuCl/PPh_3_	Cs_2_CO_3_	60	52	89:11	99:1
4	CuCl/PPh_3_	K_2_CO_3_	60	68	99:1	99:1
5	CuCl/PPh_3_	K_3_PO_4_	60	93	99:1	99:1
6	CuCl/PPh_3_	K_3_PO_4_	30	79	99:1	99:1

a General conditions: 1,1-diborylalkene (0.2 mmol), 3-bromoprop-1-ene (1.5 equiv), Cu salt (10 mol %), PPh_3_ (10 mol %), base (2 equiv), THF (4 mL), *T*, 16 h.

b Yields determined by NMR with naphthalene as the internal standard.

This reaction can be described as a stereoselective C–B activation of β,β-diboryl acrylates toward the synthesis of borylated (*Z*)-skipped dienoates, which to the best of our knowledge have been prepared for the first time in this work. Our methodology guarantees the control of the stereoselectivity, under the convenient CuCl/PPh_3_ catalytic system, and complements the reported protocols based on catalytic allylboration of alkynes for the synthesis of borylated skipped dienes.^18^ With the established reaction conditions presented above, we explored the scope of this Cu-catalyzed stereoselective deborylative allylic alkylation reaction, as summarized in Table 2. The reaction was performed well with methyl 3,3′-bispinacolboryl propenoate (**1b**) and 3-bromoprop-1-ene, demonstrating the compatibility of an alternative ester group (Table 2, entries 1 and 2, respectively). The reaction of **1a** with 3-bromo-3,3-difluoroprop-1-ene allowed the formation of perfluorinated (*Z*)-skipped product **4**, suggesting that the C–C coupling might proceed through an S_N_2′ mechanism (Table 2, entry 2). An additional substituent at the R_2_ position of the allyl halide is tolerated, as shown by the efficient allylic coupling for both 3-bromo-2-methylprop-1-ene and (3-bromoprop-1-en-2-yl)cyclopentane (Table 2, entries 3 and 4, respectively). Interestingly, even sterically hindered aryl and benzyl groups in this position were tolerated with the Cu-catalyzed strereoselective deborylative allylic alkylation reaction (Table 2, entries 5 and 6, respectively).

**Table 2:**
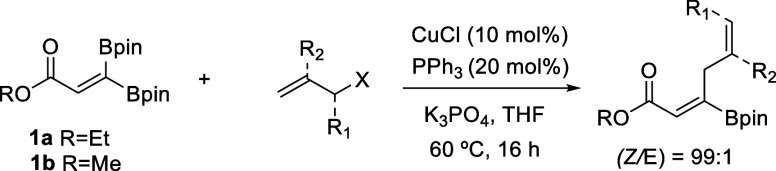

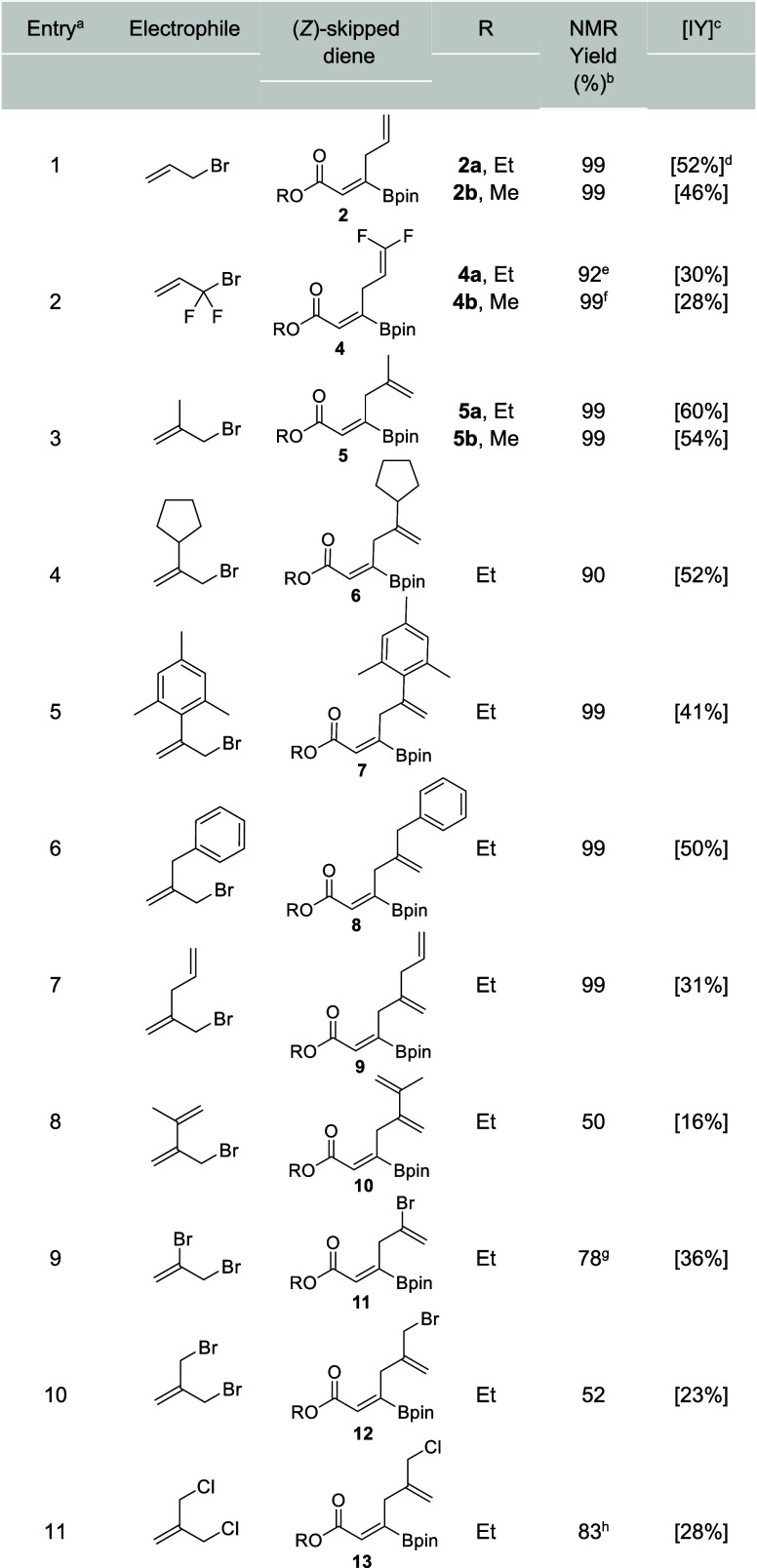
Substrate Scope for the Stereoselective Cu-Catalyzed Allylic Alkylation of β,β-Diborylacrylates **1a** and **1b**

a General conditions: 1,1-diborylalkene (0.2 mmol), allyl halide (1.5 equiv), CuCl (10 mol %), PPh_3_ (20 mol %), K_3_PO_4_ (2 equiv), THF (4 mL), 60 °C, 16 h.

b Yields determined by NMR with naphthalene as the internal standard.

c Isolated yield.

d NMR yield of 78%, isolated yield of 44% on a 1 mmol scale.

e Yield of **3a** of 6%.

f Yield of **3b** of 8%.

g Yield of **3a** of 18%.

h Yield of **3a** of 8%.

The inclusion of unsaturated functional groups in 2-(bromomethyl)penta-1,4-diene and 2-(bromomethyl)-3-methylbuta-1,3-diene proved to be compatible with C–C bond formation through allylic coupling, providing access to double skipped system **9** and double diene product **10**, although the latter in moderate yield (Table 2, entries 7 and 8, respectively). When we studied the allylic coupling of **1a** with 2,3-dibromoprop-1-ene, skipped (*Z*)-diene **11** was exclusively formed as a result of a chemoselective C–Br coupling, together with the protodeborylated byproduct (18%) (Table 2, entry 9). Eventually, the reactivity of **1a** with 3-bromo-2-(bromomethyl)prop-1-ene and 3-chloro-2-(chloromethyl)prop-1-ene provided access to polyfunctionalized products **12** and **13**, respectively, with the remaining C(sp^3^)–halide functionality for downstream transformations (Table 2, entries 10 and 11, respectively). Isolated yields are modest due to the instability of the C(sp^2^)–Bpin fragment under the purification conditions, despite differently treated silica species being used as stationary phases. According to the overall reactivity found, we suggest that the Cu-catalyzed allylic alkylation between β,β-diboryl acrylates and the allyl halides depicted in Table 2 might include an S_N_2′ mechanism. This is in agreement with the reported copper-catalyzed S_N_2′-selective allylic alkylation reactions involving *gem*-diborylalkanes^19−25^ or *gem*-diborylalkenes.^11^ Aiming to generalize this S_N_2′ allylic coupling, we explored the most challenging Cu-catalyzed coupling between **1a** and (*E*)-1-bromobut-2-ene and observed the formation of only product **14**, consistent with the favored γ selectivity, although the yield was only moderate presumably due to the greater steric hindrance (Scheme 3). Similarly, the coupling between **1a** and (*E*)-1,4-dibromobut-2-ene or (*E*)-1,4-dichlorobut-2-ene generated exclusively γ-selective products **15** and **16**, respectively, in moderate yields (Scheme 3). Notably, the cyclic 3-bromocyclohex-1-ene was a suitable electrophile along the Cu-catalyzed allylic alkylation of **1a** and **1b**, because products **17a** and **17b** were efficiently synthesized and isolated in 61% and 56% yields, respectively (Scheme 3).

**Scheme 3:**
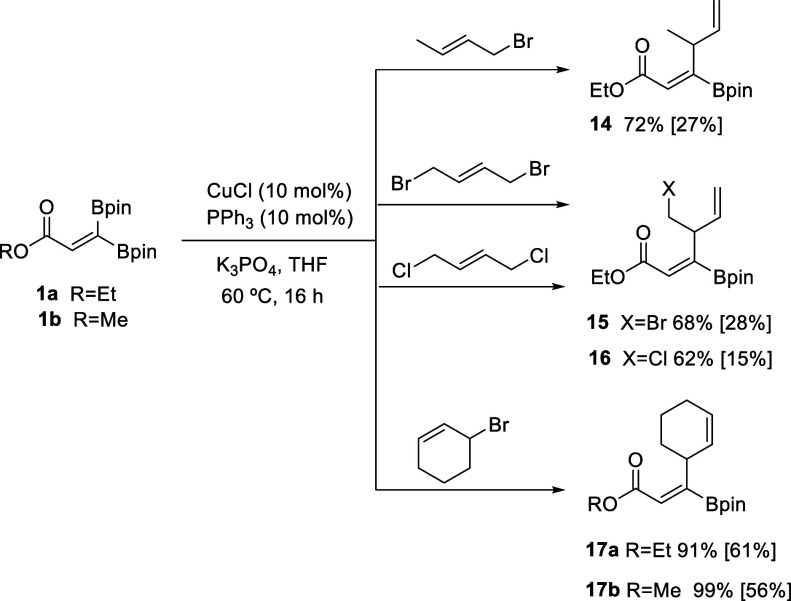
Control of the γ Selectivity in the Cu-Catalyzed Allylic Alkylation of β,β-Diborylacrylate **1** with γ-Substituted Allyl Halides General conditions: 1,1-diborylalkene (0.2 mmol), allyl halide (1.5 equiv), CuCl (10 mol %), PPh_3_ (10 mol %), K_3_PO_4_ (2 equiv), THF (4 mL), 60 °C, 16 h. Yields determined by NMR with naphthalene as the internal standard. Isolated yields in brackets.

Bearing in mind the inherent difficulty of discriminating between the two geminal Bpin–C(sp^2^)–Bpin bonds in 1,1-diborylalkenes, we can justify the preferred formation of (*Z*)-α-borylalkenyl copper(I) species, and the subsequent coupling reaction with allyl bromides, by releasing the steric repulsion between the pinacolboryl and the ester group in the *cis* disposition.^11^

The ultimate goal of the stereoselective synthesis of borylated (*Z*)-skipped dienoates is the iodo-lactonization sequence for preparing a series of borylated lactones, with the versatile C(sp^2^)–B handle for downstream functionalization of the lactone core (Scheme 4). To the best of our knowledge, borylated lactones have been achieved only through Pd-catalyzed cross-coupling pathways^26^ or electrophilic oxyboration protocols.^27^ Here, inspired by previous works on iodo-lactonization reactions by Larock^28−31^ and Knochel,^32^ we screened the feasible reaction of borylated 1,4-dienonate **2a** with I_2_, at room temperature. After 16 h, we observed complete conversion into borylated lactone **18** (Scheme 4), suggesting that the terminal double bond coordinates selectively to the iodine cation generated from I_2_ to render an iodonium intermediate followed by intramolecular rearrangement through the ester group. Interestingly, I_2_ activation of the double bond seems to be favored versus the competitive iodo-deborylation reaction,^33^ showing the remarkable stability of the Bpin group during the lactonization process. With the aim of preparing α,β-unsaturated δ-lactone moieties for straightforward access to the essential core of novel kazusamycin A derivatives,^17^ we extended the study of the iodo-lactonization to γ-substituted borylated (*Z*)-skipped dienoates **14** and **15**. Polysubstituted lactones **19** and **20** were efficiently prepared, in the presence of I_2_ at room temperature, with a notable preference for the *cis* stereoisomer in each case (Scheme 4). It is worth noting that fused lactone **21** was synthesized from borylated (*Z*)-skipped dienoate **17a**, as an exclusive *cis* stereoisomer, being isolated in 96% yield (Scheme 4). The relative configuration of the C–I bond with respect to the fused C–H bonds was unambiguously assigned by one-dimensional NMR NOE experiments and X-ray single-crystal diffraction analysis of bicycle **21**, synthesized as a single diastereoisomer (Scheme 4).

**Scheme 4:**
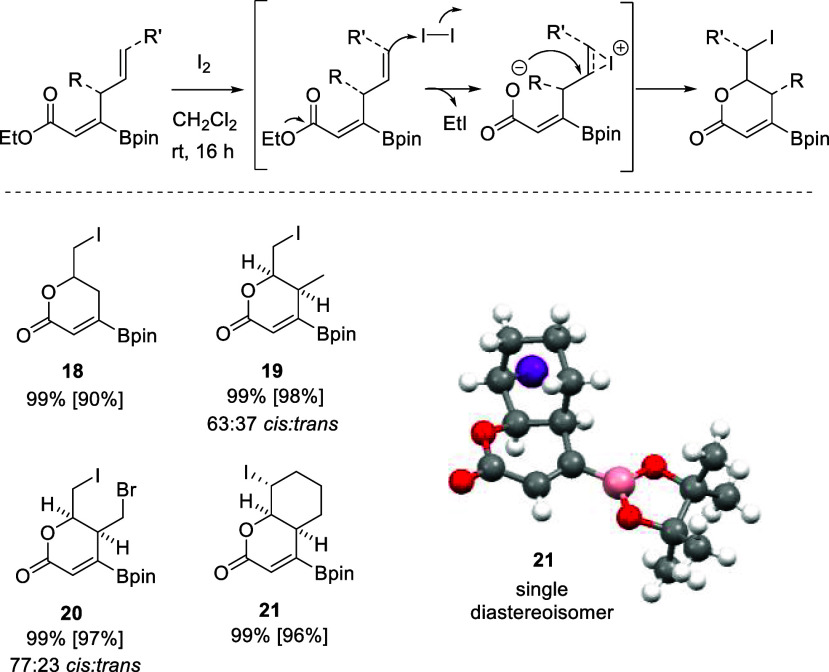
Iodo-lactonization of Borylated (*Z*)-Skipped Dienoates and X-ray Diffraction of Bicycle **21** General conditions: 1,4-dienonate (0.2 mmol), I_2_ (3 equiv), CH_2_Cl_2_ (2 mL), rt, 16 h. Yields determined by NMR with naphthalene as the internal standard. Isolated yields in brackets. In the ORTEP drawing, thermal ellipsoids are drawn at the 50% level.

We conducted the global transformation of β,β-diboryl acrylate **1a** into lactones **18**–**21**, through one-pot, two-step allylic coupling/lactonization, and the overall yields were similar to those involving the purification of the intermediate borylated 1,4-dienoates. We also studied the iodo-lactonization of borylated 1,4-dienonate **4a** with I_2_, but the reaction was not completed, even at longer reaction times, probably due to the less nucleophilic nature of the *gem*-difluoro-substituted terminal alkene. Purification of the corresponding borylated lactone turned out to be operationally difficult. To circumvent this obstacle, we strategically planned the cross-coupling of **4a** with PhI, prior to the iodo-lactonization, in the presence of Pd(PPh_3_)_4_/K_2_CO_3_, and arylated 1,4-dienonate **22** could be isolated in high yields (Scheme 5). Subsequent iodo-lactonization of **22** with I_2_ resulted in the formation of lactone **23**, which contains the pending CF_2_I group. Current interest in difluoroalkyl iodide motifs is due to its suitability as a surrogate model for the construction of alkyl–CF_2_–alkyl bonds via site-selective coupling reactions.^34,35^ Next, we explored the iodo-lactonization of borylated (*Z*)-skipped dienoate **5a** with I_2_, and the reaction outcome showed the formation of the desired borylated lactone as the main product; however, once again, purification was not successful. However, when we conducted the Pd-catalyzed cross-coupling between **5a** and PhI, the resulting (*Z*)-skipped dienoate **24** was easily isolated in high yields (Scheme 5) and successful iodo-lactonization was achieved in the presence of I_2_, generating α,β-unsaturated δ-lactone **25**, containing a quaternary carbon, in a synthetically useful isolated yield.

**Scheme 5:**
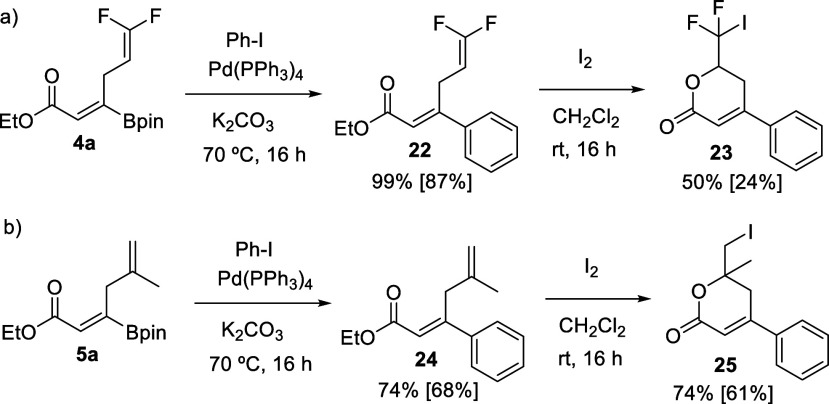
Pd-Catalyzed Cross-Coupling of Borylated 1,4-Dienoates **4a** and **5a**, Followed by an Iodo-lactonization Step General conditions for cross-coupling: borylated 1,4-dienonate (0.2 mmol), Pd(PPh_3_)_4_ (5 mol %), PhI (2 equiv), K_2_CO_3_ (2 equiv), 70 °C, 16 h. General conditions for iodo-lactonization: arylated 1,4-dienonate (0.2 mmol), I_2_ (3 equiv), CH_2_Cl_2_ (2 mL), rt, 16 h. Yields determined by NMR with naphthalene as the internal standard. Isolated yields in brackets.

Alternatively, we explored the Pd-catalyzed cross-coupling reaction between the borylated lactones and PhI to illustrate the ability to functionalize the Bpin moiety on the lactone core in the last step. When borylated lactone **19** [as a mixture of stereoisomers (63:37 *cis*:*trans*)] was reacted with PhI in the presence of Pd(PPh_3_)_4_/K_2_CO_3_, both stereoisomers evolved to the corresponding coupled products (Scheme 6a), although the less sterically hindered *trans* stereoisomer suffered from H–I elimination, which generated the corresponding exocyclic alkene that was subsequently isomerized to deliver α-pyrone **27**.^36^ Functionalized *cis*-lactone **26** was the major product isolated (Scheme 6a), resulting in an appropriate core for the synthesis of kazusamycin A derivatives.^17^ The single diastereoisomer **21** was also coupled with PhI, and the resulting lactone **28** was quantitatively formed and isolated in 90% yield (Scheme 6b). Finally, we conducted the Cu-catalyzed borylation of **21** with B_2_pin_2_ and were delighted to observe the formation of the new β,β-diborylated lactone **29**, increasing the size of the polyfunctionalized platform through the *gem*-diboron moiety, becoming the first lactone with β,β-diborylated motifs (Scheme 6b).

**Scheme 6:**
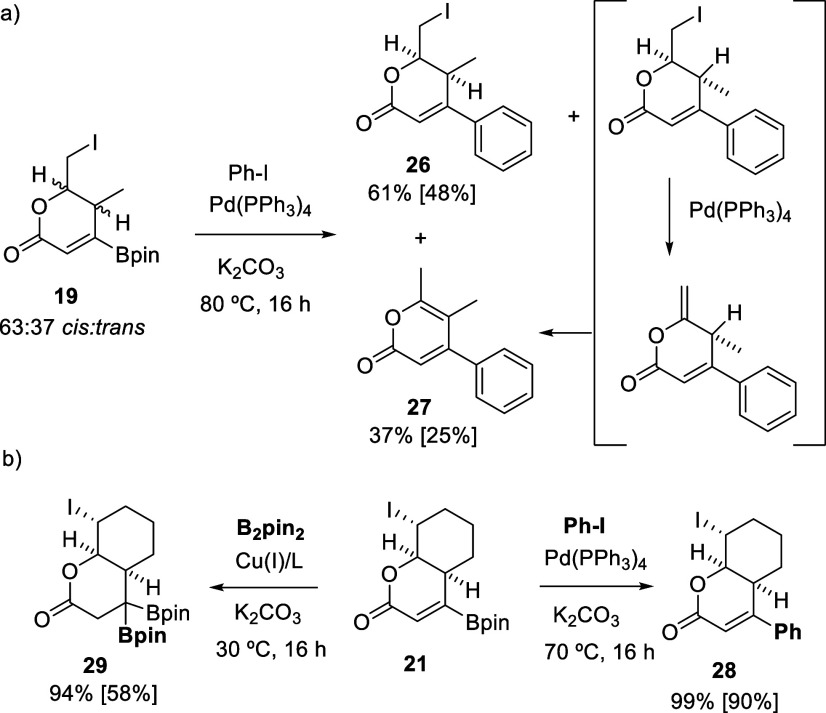
Pd-Catalyzed Cross-Coupling of Borylated Lactones **19** and **21** General conditions for cross-coupling: borylated lactone (0.2 mmol), Pd(PPh_3_)_4_ (5 mol %), PhI (2 equiv), K_2_CO_3_ (2 equiv), THF, 70 °C, 16 h. General conditions for β-borylation: borylated lactone (0.2 mmol), [Cu(MeCN)_4_]PF_6_ (5 mol %), P^n^Bu_3_ (10 mol %), B_2_pin_2_ (1.2 equiv), K_2_CO_3_ (10 mol %), THF, 30 °C, 16 h. Yields determined by NMR with naphthalene as the internal standard. Isolated yields in brackets.

In summary, we have disclosed a stereoselective C–B activation of β,β-diborylacrylates forming exclusively the (*Z*)-α-borylalkenyl copper(I) key intermediate for subsequent selective allylic alkylation reactions. The new borylated (*Z*)-skipped dienoates followed the iodo-lactonization sequence to deliver polysubstituted borylated lactone cores that might have potential in drug discovery. Subsequent Pd-catalyzed cross-coupling reactions and Cu-catalyzed β-borylation sequences illustrated the potential of the polyfunctionalization platforms.

## Data Availability

The data underlying this study are available in the published article and its Supporting Information.

## Supporting Information Available

Experimental procedures, product characterization, and spectra (PDF)
