# Supplementary material for: Stereoselective Control of the Cu Activation of β,β-Diboryl Acrylates for Allylic Coupling Protocols with Concomitant Lactonization
Source: Org Lett. 2023 Dec 15;26(14):2821–6. doi: 10.1021/acs.orglett.3c03640 (PMC11020160; doi:10.1021/acs.orglett.3c03640)
Supplement: Supplementary file 1 — ol3c03640_si_001.pdf [file ol3c03640_si_001.pdf]

Stereoselective control on Cu activation of β,β-diborylacrylates for allylic coupling protocols with concomitant lactonization

Mireia Pujol,[a] María Méndez,[b] Elena Fernández[a*]*

[a]Faculty of Chemistry, University Rovira i Virgili, 43007 Tarragona, Spain.
[b]Integrated Drug discovery, R&D, Sanofi Aventis Deutschland GmbH, Industriepark Höchst, 65926 Frankfurt am Main, Germany.

Contents:

General information
General procedure for the synthesis of β,β -diborylacrylates
General procedure for stereoselective Cu activation of β,β-diborylacrylates and nucleophilic allylic coupling
General procedure for iodolactonization reaction of borylated (*Z*)-skipped dienoates
General procedure for the Suzuki-Miyaura cross coupling
General procedure for the Cu-catalyzed β-borylation
Characterization data for borylated (*Z*)-skipped dienoates
Characterization data for borylated lactones
Characterization data for coupled products
Characterization data for α–pyrone **27**
^{1}H, ^{13}C, ^{11}B Spectra for borylated (*Z*)-skipped dienoates
^{1}H, ^{13}C, ^{11}B Spectra for borylated lactones
^{1}H, ^{13}C, ^{11}B Spectra for coupled products
^{1}H, ^{13}C, ^{11}B Spectra for α-pyrone **27**
X-ray single-crystal diffraction analysis for product **21**
References

General Information

Solvents and reagents: Solvents and reagents were obtained from commercial suppliers and dried and/or purified (if needed) by standard procedures. Diboron reagents were purchased from Ally Chem and used without further purification. All reactions were conducted in oven and flame-dried glassware under an inert atmosphere of argon, using Schlenk-type techniques. *Flash chromatography* was performed on standard silica gel (Merck Kieselgel 60 F254 400-630 mesh). *Thin layer chromatography* was performed on Merck Kieselgel 60 F254 which was developed using standard visualizing agents: UV fluorescence (254 and 366 nm) or potassium permanganate/Δ. *NMR spectra* were recorded at a Varian Goku 400 or a Varian Mercury 400 spectrometer. ^{1}H NMR and $^{13}C\{^{1}H\}$ NMR chemical shifts (δ) are reported in ppm with the solvent resonance as the internal standard ($CDCl_3$: 7.26 ppm (^{1}H) and $CDCl_3$: 77.16 ppm (^{13}C)). $^{11}B\{^{1}H\}$ NMR chemical shifts (δ) are reported in ppm relative to $(CH_3)_2O\cdots BF_3$. Data are reported as follows: chemical shift, multiplicity (s = singlet, d = doublet, t = triplet, q = quartet, hept = heptuplet, br = broad, m = multiplet), coupling constants (Hz) and integration. *High resolution mass spectra (HRMS)* were recorded using a 6210 Time of Flight (TOF) mass spectrometer from Agilent Technologies (Waldbronn, Germany) with an ESI interface and it was performed at the Servei de Recursos Científics i Tècnics (Universitat Rovira i Virgili, Tarragona) or using a BIOTOF II Time of Flight (TOF) mass spectrometer from Bruker with an APCI interface or EI interface and it was performed at the Unidade de Espectrometría de Masas e Proteómica (Universidade de Santiago de Compostela, Santiago de Compostela). GC-MS analyses were performed on a HP6890 gas chromatograph and an Agilent Technologies 5973 Mass selective detector (Waldbronn, Germany) equipped with an achiral capillary column HP-5 (30m, 0.25mm i. d., 0.25µm thickness) using He as the carrier gas. Full sphere single crystal data collection for product **21** where performed at 100 K on a Bruker Kappa Apex II DUO diffractometer equiped with a Cryostream 700 plus low temperature device, a microsource anode with Mo *Kα* (λ= 0.71073 Å).

General procedure for the synthesis of β,β-diborylacrylates[1]

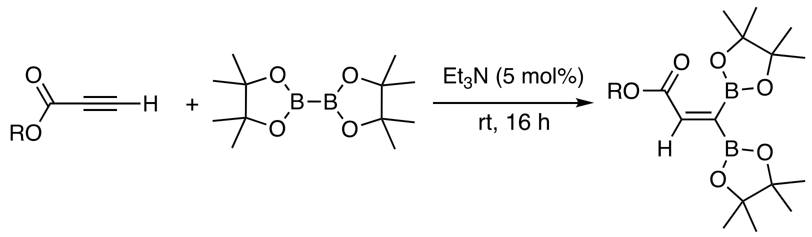

Bis(pinacolato)diboron (B_2pin_2, 3 mmol, 1 equiv) was placed in a vial containing a magnetic stirring bar. The vial was sealed with a Teflon®-coated silicon rubber septum and the vial was evacuated and filled with argon. Terminal alkyne (3 mmol, 1 equiv) and Et_3N (0.15 mmol, 5 mol%) were sequentially added to the vial. After 16 h stirring at rt, the mixture was filtered through a short plug of silica gel, which was then washed with ethyl acetate. The solvent was removed under reduced pressure to afford the desired product in quantitative yield.

General procedure for stereoselective Cu activation of β,β-diborylacrylates and nucleophilic allylic coupling

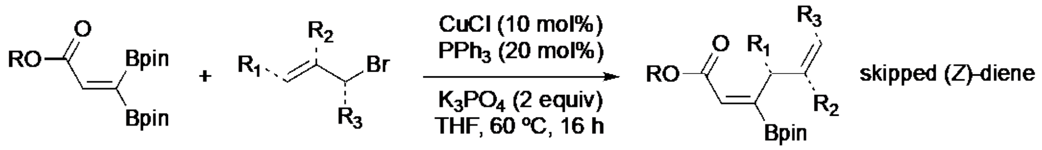

In a flamed Schlenk-tube equipped with a magnetic stir bar, β,β-diborylacrylate **1a** (1 mmol, 1 equiv, 352.04 mg), CuCl (0.1 mmol, 10 mmol %, 9.90 mg), PPh_3 (0.2 mmol, 20 mol%, 52.46 mg) and K_3PO_4 (2 mmol, 2 equiv, 424.53 mg) were added in THF (20 mL) under argon atmosphere. Next, allylbromide (1.5 mmol, 1.5 equiv, 0.130 mL) was introduced into the reaction mixture. After being stirred at 60 °C in an oil bath for 16 h, the reaction was concentrated under vacuum and the NMR yield was calculated by comparison to an internal standard (naphthalene). The crude residue was purified by silica gel flash chromatography to afford the desired product **2a** (78% NMR yield, 44% isolated yield, 118 mg).

General procedure for iodolactonization reaction of borylated (*Z*)-skipped dienoates

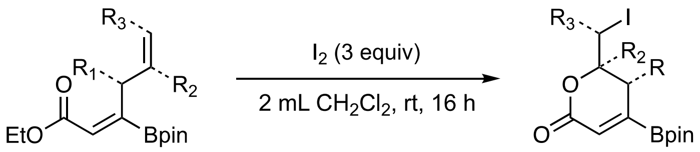

In a flamed Schlenk-tube equipped with a magnetic stir bar, the borylated (*Z*)-skipped dienoate (0.2 mmol) was mixed with CH_2Cl_2 (2 mL) under argon atmosphere, and I_2 (3 equiv) were added to the reaction mixture. After being stirred at rt for 16 h, the reaction was quenched with 15 mL of $Na_2S_2O_3$, extracted with Et_2O for three times and dried with $MgSO_4$ anhydrous. Finally, the crude of the reaction was concentrated under vacuum to afford the desired product in quantitative yields.

General procedure for Suzuki-Miyaura cross-coupling reaction

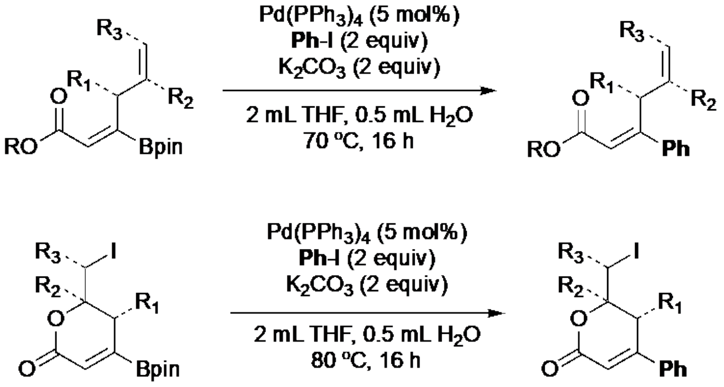

In a flamed Schlenk-tube equipped with a magnetic stir bar, $Pd(PPh_3)_4$ (0.10 mmol, 5 mol%), the substrate (skipped (*Z*)-dienoate or borylated lactone) (0.2 mmol, 1 equiv), PhI (0.4 mmol, 2 equiv) and K_2CO_3 (0.4 mmol, 2 equiv) were added in THF (2 mL) and water (0.5 mL) under argon atmosphere. The reaction mixture was stirred at 70 °C or 80 ºC in an oil bath for 16 h. After that, the reaction was concentrated under vacuum and the crude was purified by flash chromatography to afford the desired product.

General procedure for the Cu-catalyzed β-borylation

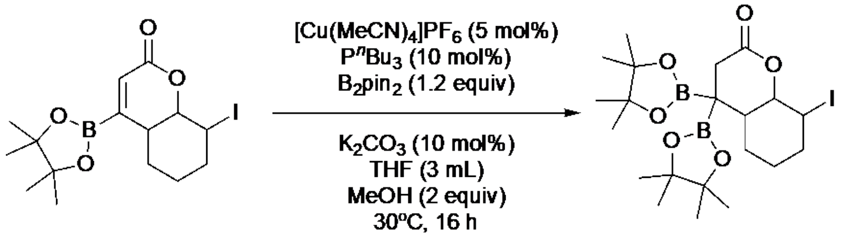

In a flamed Schlenk-tube, equipped with a magnetic stir bar, $[Cu(MeCN)_4]PF_6$ (5 mol%), bis(pinacolato)diboron (1.2 equiv) and P^nBu_3 (10 mol%) were added in THF (1 mL). Next, K_2CO_3 (10 mol%) in THF (1 mL) was added in the reaction mixture. Then borylated lactone **21** (1 equiv, 0.2 mmol) in THF (1 mL) was added dropwise. After 16 h stirring at 30 °C in an oil bath, the reaction mixture was filtered over Celite. The organic extracts were then concentrated in vacuo. The crude product was purified by flash chromatography.

Characterization data for borylated (*Z*)-skipped dienoates

Ethyl(*Z*)-3-(4,4,5,5-tetramethyl-1,3,2-dioxaborolan-2-yl)hexa-2,5-dienoate (2a)

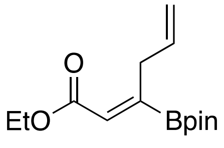

2a

The product was purified by flash chromatography using as eluent a mixture of petroleum ether/ethyl acetate (100:1). The product was isolated as a pail yellowish oil (47 mg, 0.177 mmol, 52%).

^{1}H NMR (400 MHz, CDCl$_3$) δ= 6.44 (s, 1H), 5.88 (ddt, *J* = 16.7, 10.1, 6.5 Hz, 1H), 5.05 (dd, *J* = 17.2, 1.8 Hz, 1H), 4.96 (dd, *J* = 10.1, 1.5 Hz, 1H), 4.17 (q, *J* = 7.1 Hz, 2H), 3.44 (dd, *J* = 6.5, 1.4 Hz, 2H), 1.27 (t, *J* = 7.2, 3H), 1.25 (s, 12H).

^{13}C NMR (100 MHz, CDCl$_3$) δ= 166.0, 135.9, 130.4, 115.6, 84.2, 60.0, 34.1, 24.8, 14.3.

^{11}B NMR (129 MHz, CDCl$_3$) δ= 30.33.

HRMS-(ESI+) for $C_{14}H_{24}BO_4$ [M+H]$^+$: calculated 267.1765; found: 267.1755.

Methyl (*Z*)-3-(4,4,5,5-tetramethyl-1,3,2-dioxaborolan-2-yl)hexa-2,5-dienoate (2b)

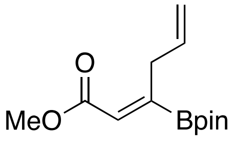

2b

The product was purified by flash chromatography using as eluent a mixture of petroleum ether/ethyl acetate (100:1). The product was isolated as a pail yellowish oil (76 mg, 0.301 mmol, 46%).

^{1}H NMR (400 MHz, CDCl$_3$) δ= 6.43 (s, 1H), 5.86 (ddt, *J* = 16.7, 10.1, 6.5 Hz, 1H), 5.03 (dd, *J* = 17.2, 1.7 Hz, 1H), 4.95 (dd, *J* = 10.1, 1.5 Hz, 1H), 3.69 (s, 3H), 3.43 (dd, *J* = 6.4, 1.4 Hz, 2H), 1.24 (s, 12H).

^{13}C NMR (100 MHz, CDCl$_3$) δ= 166.4, 135.8, 129.8, 115.6, 84.2, 51.2, 34.1, 24.8.

^{11}B NMR (129 MHz, CDCl$_3$) δ= 30.09.

HRMS-(ESI+) for $C_{13}H_{22}BO_4$ [M+H]$^+$: calculated 253.1606; found: 253.1610.

Ethyl (*Z*)-6,6-difluoro-3-(4,4,5,5-tetramethyl-1,3,2-dioxaborolan-2-yl)hexa-2,5-dienoate (4a)

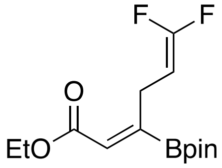

4a

The product was purified by flash chromatography using as eluent a mixture of petroleum ether/ethyl acetate (100:1). The product was isolated as a pail yellowish oil (39 mg, 0.12 mmol, 30%).

^{1}H NMR (400 MHz, CDCl$_3$) δ= 6.43 (s, 1H), 4.28 (ddd, *J* = 24.8, 8.1, 2.4 Hz, 1H), 4.18 (q, *J* = 7.1 Hz, 2H), 3.33 (d, *J* = 8.1 Hz, 2H), 1.29 (t, *J* = 7.1 Hz, 3H), 1.27 (s, 12H).

^{13}C NMR (100 MHz, CDCl$_3$) δ= 165.8, 156.6 (dd, $^1J_{C\text{-}F}$ = 288.8, 284.6 Hz), 130.9, 84.4, 76.5 (dd, $^2J_{C\text{-}F}$ = 24.1, 19.7 Hz), 60.2, 24.8, 22.8 (d, $^3J_{C\text{-}F}$ = 4.8 Hz), 14.3.

^{11}B NMR (129 MHz, CDCl$_3$) δ= 30.22.

^{19}F NMR (377 MHz, CDCl$_3$) δ= -89.09 (d, *J* = 45.1 Hz), -90.19 (dd, *J* = 45.1, 24.8 Hz).

HRMS-(ESI+) for $C_{14}H_{22}BF_2O_4$ [M+H]$^+$: calculated 303.1574; found: 303.1565.

Methyl (*Z*)-6,6-difluoro-3-(4,4,5,5-tetramethyl-1,3,2-dioxaborolan-2-yl)hexa-2,5-dienoate (4b)

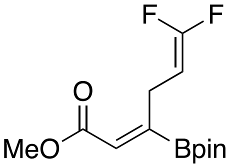

4b

The product was purified by flash chromatography using as eluent a mixture of petroleum ether/ethyl acetate (100:1). The product was isolated as a pail yellowish oil (60 mg, 0.208 mmol, 28%).

^{1}H NMR (400 MHz, CDCl$_3$) δ= 6.43 (s, 1H), 4.28 (ddd, *J* = 25.2, 8.0, 2.6 Hz, 1H), 3.73 (s, 3H), 3.33 (dd, *J* = 8.0, 1.5 Hz, 2H), 1.27 (s, 12H).

^{13}C NMR (100 MHz, CDCl$_3$) δ= 166.2, 156.7 (dd, $^1J_{C\text{-}F}$= 288.5, 284.8 Hz), 130.4, 84.5, 76.5 (dd, $^2J_{C\text{-}F}$ = 24.2, 19.8 Hz), 51.4, 24.8, 22.9 (d, $^3J_{C\text{-}F}$ = 5.1 Hz).

^{11}B NMR (129 MHz, CDCl$_3$) δ= 29.85.

^{19}F NMR (377 MHz, CDCl$_3$) δ= -89.04 (d, *J* = 44.4 Hz), -90.18 (dd, *J* = 44.4, 25.0 Hz).

HRMS-(ESI+) for $C_{13}H_{20}BF_2O_4$ [M+H]$^+$: calculated 289.1417; found: 289.1418.

Ethyl (*Z*)-5-methyl-3-(4,4,5,5-tetramethyl-1,3,2-dioxaborolan-2-yl)hexa-2,5-dienoate (5a)

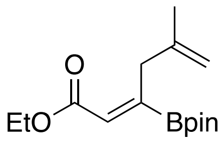

5a

The product was purified by flash chromatography using as eluent a mixture of petroleum ether/ethyl acetate (100:1). The product was isolated as a pail yellowish oil (53 mg, 0.189 mmol, 60%).

^{1}H NMR (400 MHz, CDCl$_3$) δ= 6.47 (s, 1H), 4.71 (d, *J* = 2.2, Hz 1H), 4.61 (d, *J* = 2.2 Hz, 1H), 4.16 (q, *J* = 7.1 Hz, 2H), 3.42 (s, 2H), 1.75 (s, 3H), 1.27 (t, *J* = 7.1 Hz, 3H), 1.24 (s, 12H).

^{13}C NMR (100 MHz, CDCl$_3$) δ= 166.0, 144.3, 130.5, 110.8, 84.2, 60.0, 37.2, 24.7, 23.4, 14.3.

^{11}B NMR (129 MHz, CDCl$_3$) δ= 30.49.

HRMS-(ESI+) for $C_{15}H_{26}BO_4$ [M+H]$^+$: calculated 281.1919; found: 281.1920.

Methyl (*Z*)-5-methyl-3-(4,4,5,5-tetramethyl-1,3,2-dioxaborolan-2-yl)hexa-2,5-dienoate (5b)

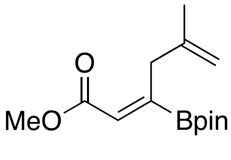

5b

The product was purified by flash chromatography using as eluent a mixture of petroleum ether/ethyl acetate (100:1). The product was isolated as a pail yellowish oil (102 mg, 0.383 mmol, 54%).

^{1}H NMR (400 MHz, CDCl$_3$) δ= 6.47 (s, 1H), 4.71 (d, *J* = 2.1 Hz, 1H), 4.61 (d, *J* = 2.1 Hz, 1H), 3.70 (s, 3H), 3.42 (s, 2H), 1.75 (s, 3H), 1.25 (s, 12H).

^{13}C NMR (100 MHz, CDCl$_3$) δ= 166.5, 144.3, 130.0, 110.9, 84.2, 51.2, 37.2, 24.7, 23.4.

^{11}B NMR (129 MHz, CDCl$_3$) δ= 29.67.

HRMS-(ESI+) for $C_{14}H_{24}BO_4$ [M+H]$^+$: calculated 267.1762; found: 267.1766.

Ethyl (*Z*)-5-cyclopentyl-3-(4,4,5,5-tetramethyl-1,3,2-dioxaborolan-2-yl)hexa-2,5-dienoate (6)

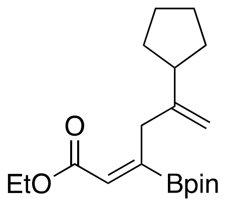

6

The product was purified by flash chromatography using as eluent a mixture of petroleum ether/ethyl acetate (100:1). The product was isolated as a pail yellowish oil (57 mg, 0.171 mmol, 52%).

^{1}H NMR (400 MHz, $CDCl_3$) δ= 6.47 (s, 1H), 4.75 (bs, 1H), 4.51 (d, *J* = 2.1, Hz, 1H), 4.16 (q, *J* = 7.1 Hz, 2H), 3.46 (s, 2H), 2.46 (bs, *J* = 1H), 1.86 – 1.75 (m, 2H), 1.71 – 1.61 (m, 2H), 1.60 – 1.53 (m, 2H), 1.51 – 1.42 (m, 2H), 1.26 (t, *J* = 7.1 Hz, 3H), 1.23 (s, 12H).

^{13}C NMR (100 MHz, $CDCl_3$) δ= 166.1, 151.6, 130.1, 107.6, 84.1, 59.9, 47.1, 34.9, 31.4, 25.2, 24.7, 14.3.

^{11}B NMR (129 MHz, $CDCl_3$) δ= 30.56.

HRMS-(ESI+) for $C_{19}H_{32}BO_4$ [M+H]$^+$: calculated 335.2388; found: 335.2403.

Ethyl (*Z*)-5-mesityl-3-(4,4,5,5-tetramethyl-1,3,2-dioxaborolan-2-yl)hexa-2,5-dienoate (7)

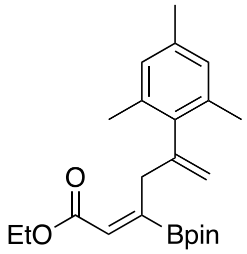

7

The product was purified by flash chromatography using as eluent a mixture of petroleum ether/ethyl acetate (100:1). The product was isolated as a pail yellowish oil (48 mg, 0.125 mmol, 41%).

^{1}H NMR (400 MHz, $CDCl_3$) δ= 6.85 (s, 2H), 6.59 (s, 1H), 5.02 (d, *J* = 1.8 Hz, 1H), 4.76 (d, *J* = 1.7 Hz, 1H), 4.14 (q, *J* = 7.1 Hz, 2H), 3.59 (s, 2H), 2.29 (s, 6H), 2.27 (bs, 3H), 1.27 (t, *J* = 7.1 Hz, 3H), 1.25 (s, 12H).

^{13}C NMR (100 MHz, $CDCl_3$) δ= 165.8, 147.1, 140.0, 136.0, 135.6, 131.9, 128.07, 113.5, 84.2, 60.0, 36.4, 24.8, 21.0, 19.8, 14.8.

^{11}B NMR (129 MHz, $CDCl_3$) δ= 30.68.

HRMS-(ESI+) for $C_{23}H_{34}BO_4$ [M+H]$^+$: calculated 385.2545; found: 385.2554.

Ethyl (Z)-5-benzyl-3-(4,4,5,5-tetramethyl-1,3,2-dioxaborolan-2-yl)hexa-2,5-dienoate (8)

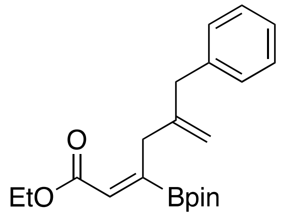

8

The product was purified by flash chromatography using as eluent a mixture of petroleum ether/ethyl acetate (100:1). The product was isolated as a pail yellowish oil (53 mg, 0.149 mmol, 50%).

^{1}H NMR (400 MHz, $CDCl_3$) δ= 7.31 – 7.26 (m, 2H), 7.24 – 7.17 (m, 3H), 6.50 (s, 1H), 4.75 (d, J = 1.7 Hz, 1H), 4.69 (d, J = 1.4 Hz, 1H), 4.14 (q, J = 7.1 Hz, 2H), 3.44 (s, 2H), 3.41 (s, 2H), 1.25 (s, 12H), 1.24 (t, J = 7.1 Hz, 3H).

^{13}C NMR (100 MHz, $CDCl_3$) δ= 166.0, 147.4, 139.7, 130.7, 129.3, 128.2, 126.0, 112.1, 84.2, 60.0, 43.7, 35.6, 24.7, 14.3.

^{11}B NMR (129 MHz, $CDCl_3$) δ= 29.96.

HRMS-(ESI+) for $C_{21}H_{30}BO_4$ $[M+H]^+$: calculated 357.2232; found: 357.2242.

Ethyl (*Z*)-4-methyl-3-(4,4,5,5-tetramethyl-1,3,2-dioxaborolan-2-yl)hexa-2,5-dienoate (9)

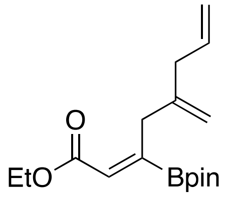

9

The product was purified by flash chromatography using as eluent a mixture of petroleum ether/ethyl acetate (100:1). The product was isolated as a pail yellowish oil (25 mg, 0.082 mmol, 31%).

^{1}H NMR (400 MHz, $CDCl_3$) δ= 6.48 (s, 1H), 5.93 – 5.76 (m, 1H), 5.12 – 4.98 (m, 2H), 4.77 (bs, 1H), 4.66 (bs, 1H), 4.16 (q, *J* = 7.1 Hz, 2H), 3.44 (s, 2H), 2.84 – 2.78 (m, 2H), 1.27 (t, *J* = 7.1 Hz, 3H), 1.24 (s, 12H).

^{13}C NMR (100 MHz, $CDCl_3$) δ= 166.0, 146.6, 136.5, 130.6, 116.1, 110.9, 84.2, 60.0, 41.5, 35.8, 24.7, 14.3.

^{11}B NMR (129 MHz, $CDCl_3$) δ= 30.62.

HRMS-(ESI+) for $C_{17}H_{28}BO_4$ $[M+H]^+$: calculated 307.2075; found: 307.2076.

Ethyl (*Z*)-6-methyl-5-methylene-3-(4,4,5,5-tetramethyl-1,3,2-dioxaborolan-2-yl)hepta-2,6-dienoate (10)

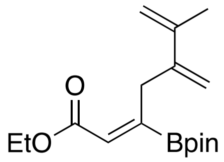

10

The product was purified by flash chromatography using as eluent a mixture of petroleum ether/ethyl acetate (100:1). The product was isolated as a pail yellowish oil (15 mg, 0.049 mmol, 16%).

^{1}H NMR (400 MHz, CDCl$_3$) δ= 6.49 (s, 1H), 5.15 (s, 1H), 5.08 (s, 1H), 4.97 (s, 1H), 4.79 (s, 1H), 4.15 (q, *J* = 7.1 Hz, 2H), 3.69 (s, 2H), 1.92 (s, 3H), 1.26 (t, *J* = 7.1 Hz, 3H), 1.23 (s, 12H).

^{13}C NMR (100 MHz, CDCl$_3$) δ= 166.0, 146.1, 143.1, 130.6, 113.0, 112.3, 84.2, 60.0, 33.2, 24.7, 21.3, 14.3.

^{11}B NMR (129 MHz, CDCl$_3$) δ= 30.20.

HRMS-(ESI+) for $C_{17}H_{28}BO_4$ [M+H]$^+$: calculated 307.2075; found: 307.2080.

Ethyl (*Z*)-5-bromo-3-(4,4,5,5-tetramethyl-1,3,2-dioxaborolan-2-yl)hexa-2,5-dienoate (11)

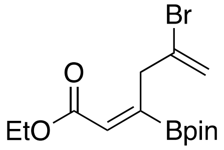

11

The product was purified by flash chromatography using as eluent a mixture of petroleum ether/ethyl acetate (100:1). The product was isolated as a pail yellowish oil (20 mg, 0.108 mmol, 36%).

^{1}H NMR (400 MHz, CDCl$_3$) δ= 6.57 (s, 1H), 5.56 (d, *J* = 1.5 Hz, 1H), 5.40 (d, *J* = 1.8Hz, 1H), 4.18 (q, *J* = 7.1 Hz, 2H), 3.89 (bs, 2H), 1.29 (t, *J* = 7.1 Hz, 3H), 1.26 (s, 12H).

^{13}C NMR (100 MHz, CDCl$_3$) δ= 165.6, 132.6, 131.2, 117.3, 84.5, 60.3, 40.5, 24.8, 14.3.

^{11}B NMR (129 MHz, CDCl$_3$) δ= 30.33.

HRMS-(ESI+) for $C_{14}H_{23}BBrO_4$ [M+H]$^+$: calculated 345.0867; found: 345.0867.

Ethyl (*Z*)-5-(bromomethyl)-3-(4,4,5,5-tetramethyl-1,3,2-dioxaborolan-2-yl)hexa-2,5-dienoate (12)

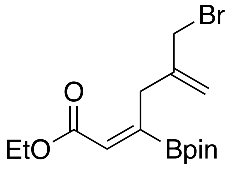

12

The product was purified by flash chromatography using as eluent a mixture of petroleum ether/ethyl acetate (100:1). The product was isolated as a pail yellowish oil (24 mg, 0.067 mmol, 23%).

^{1}H NMR (400 MHz, CDCl$_3$) δ= 6.52 (s, 1H), 5.17 (d, *J* = 1.0 Hz, 1H), 4.92 (d, *J* = 1.4 Hz, 1H), 4.18 (q, *J* = 7.1 Hz, 2H), 4.01 (s, 2H), 3.58 (s, 2H), 1.27 (t, *J* = 7.2 Hz, 3H), 1.24 (s, 12H).

^{13}C NMR (100 MHz, CDCl$_3$) δ= 165.9, 143.9, 131.6, 116.2, 84.4, 60.2, 37.2, 33.6, 24.8, 14.3.

^{11}B NMR (129 MHz, CDCl$_3$) δ= 30.63.

HRMS-(ESI+) for $C_{15}H_{25}BBrO_4$ [M+H]$^+$: calculated 359.1024; found: 359.1030.

Ethyl (*Z*)-5-(chloromethyl)-3-(4,4,5,5-tetramethyl-1,3,2-dioxaborolan-2-yl)hexa-2,5-dienoate (13)

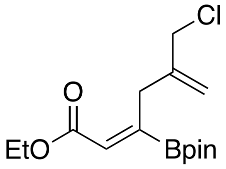

13

The product was purified by flash chromatography using as eluent a mixture of petroleum ether/ethyl acetate (100:1). The product was isolated as a pail yellowish oil (47 mg, 0.149 mmol, 28%).

^{1}H NMR (400 MHz, CDCl$_3$) δ= 6.51 (s, 1H), 5.14 (d, *J* = 1.1 Hz, 1H), 4.92 (d, *J* = 1.4 Hz, 1H), 4.17 (q, *J* = 7.1 Hz, 2H), 4.07 (s, 2H), 3.54 (s, 2H), 1.27 (t, *J* = 7.2 Hz, 3H), 1.24 (s, 12H).

^{13}C NMR (100 MHz, CDCl$_3$) δ= 165.9, 143.6, 131.5, 115.2, 84.3, 60.2, 48.6, 33.3, 24.7, 14.3.

^{11}B NMR (129 MHz, CDCl$_3$) δ= 29.89.

HRMS-(ESI+) for $C_{15}H_{25}BClO_4$ [M+H]$^+$: calculated 315.1529; found: 315.1538.

(*Z*)-4-Methyl-3-(4,4,5,5-tetramethyl-1,3,2-dioxaborolan-2-yl)hexa-2,5-dienoate (14)

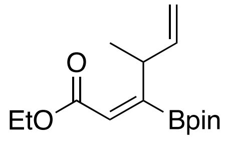

14

The product was purified by flash chromatography using as eluent a mixture of petroleum ether/ethyl acetate (100:1). The product was isolated as a pail yellowish oil (23 mg, 0.082 mmol, 27%).

^{1}H NMR (400 MHz, CDCl$_3$) δ= 6.32 (s, 1H), 6.01 (ddd, *J* = 17.3, 10.2, 7.3 Hz, 1H), 5.03 (dd, *J* = 17.3, 1.6 Hz, 1H), 4.93 (dd, *J* = 10.1, 1.5 Hz, 1H), 4.38 (bs, 1H), 4.17 (q, *J* = 7.1 Hz, 2H), 1.28 (t, *J* = 7.1 Hz, 3H), 1.25 (s, 12H), 1.21 (d, *J* = 7.0 Hz, 3H).

^{13}C NMR (100 MHz, CDCl$_3$) δ= 166.1, 142.2, 128.5, 114.0, 84.0, 60.0, 38.7, 24.8, 24.7, 19.8, 14.3.

^{11}B NMR (129 MHz, CDCl$_3$) δ= 30.01.

HRMS-(ESI+) for $C_{15}H_{26}BO_4$ [M+H]$^+$: calculated 281.1919; found: 281.1920.

Ethyl (*Z*)-4-(bromomethyl)-3-(4,4,5,5-tetramethyl-1,3,2-dioxaborolan-2-yl)hexa-2,5-dienoate (15)

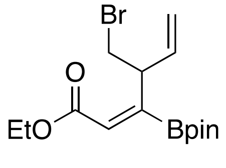

15

The product was purified by flash chromatography using as eluent a mixture of petroleum ether/ethyl acetate (100:1). The product was isolated as a pail yellowish oil (31 mg, 0.086 mmol, 28%).

^{1}H NMR (400 MHz, CDCl$_3$) δ= 6.53 (s, 1H), 5.92 (ddd, *J* = 17.2, 10.2, 7.9 Hz, 1H), 5.19 (dd, *J* = 17.2, 1.4 Hz, 1H), 5.10 (dd, *J* = 10.2, 1.2 Hz, 1H), 4.70 (bs, 1H), 4.19 (q, *J* = 7.1 Hz, 2H), 3.72 (t, *J* = 9.3 Hz, 1H), 3.52 (dd, *J* = 9.5, 7.2 Hz, 1H), 1.30 (t, *J* = 7.1 Hz, 3H), 1.27 (s, 12H).

^{13}C NMR (100 MHz, CDCl$_3$) δ= 165.7, 137.6, 132.3, 117.6, 84.3, 60.3, 46.5, 35.3, 24.8, 14.3.

^{11}B NMR (129 MHz, CDCl$_3$) δ= 29.86.

HRMS-(ESI+) for $C_{15}H_{25}BBrO_4$ [M+H]$^+$: calculated 359.1024; found: 359.1040.

Ethyl (*Z*)-4-(chloromethyl)-3-(4,4,5,5-tetramethyl-1,3,2-dioxaborolan-2-yl)hexa-2,5-dienoate (16)

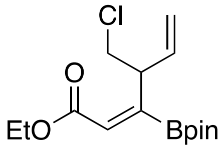

16

The product was purified by flash chromatography using as eluent a mixture of petroleum ether/ethyl acetate (100:1). The product was isolated as a pail yellowish oil (15 mg, 0.048 mmol, 15%).

^{1}H NMR (400 MHz, CDCl$_3$) δ= 6.54 (s, 1H), 5.93 (ddd, *J* = 17.8, 10.2, 7.8 Hz, 1H), 5.19 (dd, *J* = 17.1, 1.4 Hz, 1H), 5.10 (dd, *J* = 10.3, 1.2 Hz, 1H), 4.67 (bs, 1H), 4.19 (q, *J* = 7.1 Hz, 2H), 3.83 (dd, *J* = 10.4, 8.8 Hz, 1H), 3.67 (dd, *J* = 10.4, 7.2 Hz, 1H), 1.30 (t, *J* = 7.1 Hz, 3H), 1.27 (s, 12H).

^{13}C NMR (100 MHz, CDCl$_3$) δ= 165.7, 137.0, 132.5, 117.7, 84.3, 60.3, 46.6, 46.4, 24.8, 14.3.

^{11}B NMR (129 MHz, CDCl$_3$) δ= 30.17.

HRMS-(ESI+) for $C_{15}H_{25}BClO_4$ [M+H]$^+$: calculated 315.1529; found: 315.1529.

Ethyl (*Z*)-3-(cyclohex-2-en-1-yl)-3-(4,4,5,5-tetramethyl-1,3,2-dioxaborolan-2-yl)acrylate (17a)

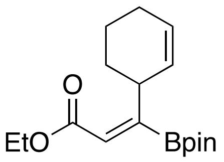

17a

The product was purified by flash chromatography using as eluent a mixture of petroleum ether/ethyl acetate (100:1). The product was isolated as a pail yellowish oil (58 mg, 0.189 mmol, 61%).

^{1}H NMR (400 MHz, CDCl$_3$) δ= 6.25 (s, 1H), 5.77 – 5.67 (m, 1H), 5.45 – 5.38 (m, 1H), 4.19 (bs, 1H), 4.15 (q, *J* = 7.1 Hz, 2H), 2.06 – 1.96 (m, 2H), 1.84 – 1.75 (m, 2H), 1.70 – 1.55 (m, 2H), 1.27 (d, *J* = 7.1 Hz, 3H), 1.25 (s, 6H), 1.23 (s, 6H).

^{13}C NMR (100 MHz, CDCl$_3$) δ= 166.1, 129.2, 128.0, 127.9, 83.9, 59.9, 37.4, 28.2, 24.9, 24.9, 24.6, 22.2, 14.3.

^{11}B NMR (129 MHz, CDCl$_3$) δ= 30.29.

HRMS-(ESI+) for $C_{17}H_{28}BO_4$ [M+H]$^+$: calculated 307.2075; found: 307.2075.

Methyl (*Z*)-3-(cyclohex-2-en-1-yl)-3-(4,4,5,5-tetramethyl-1,3,2-dioxaborolan-2-yl)acrylate (17b)

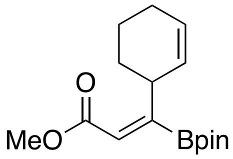

17b

The product was purified by flash chromatography using as eluent a mixture of petroleum ether/ethyl acetate (100:1). The product was isolated as a pail yellowish oil (140 mg, 0.423 mmol, 56%).

^{1}H NMR (400 MHz, CDCl$_3$) δ= 6.25 (s, 1H), 5.78 – 5.68 (m, 1H), 5.45 – 5.38 (m, 1H), 4.22 – 4.15 (bs, 1H), 3.69 (s, 3H), 2.11 – 1.94 (m, 2H), 1.84 – 1.74 (m, 2H), 1.71 – 1.56 (m, 2H), 1.25 (s, 6H), 1.24 (s, 6H).

^{13}C NMR (100 MHz, CDCl$_3$) δ= 166.5, 129.2, 127.9, 127.5, 84.0, 51.2, 37.5, 28.2, 24.9, 24.9, 24.6, 22.2.

^{11}B NMR (129 MHz, CDCl$_3$) δ= 30.13.

HRMS-(ESI+) for $C_{16}H_{26}BO_4$ [M+H]$^+$: calculated 293.1919; found: 293.1927.

Characterization data for lactones

6-(Iodomethyl)-4-(4,4,5,5-tetramethyl-1,3,2-dioxaborolan-2-yl)-5,6-dihydro-2H-pyran-2-one (18)

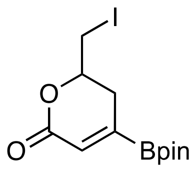

18

The product was purified by quenching with 15 mL of $Na_2S_2O_3$ and extraction (3 x Et_2O). The product was isolated as a yellow oil (86 mg, 0.236 mmol, 90%).

^{1}H NMR (400 MHz, $CDCl_3$) δ= 6.48 (s, 1H), 4.33 (m, 1H), 3.43 – 3.31 (m, 2H), 2.71 (ddd, *J* = 18.3, 3.7, 1.0 Hz, 1H), 2.42 (ddd, *J* = 18.2, 11.8, 2.9 Hz, 1H), 1.28 (s, 12H).

^{13}C NMR (100 MHz, $CDCl_3$) δ= 162.9, 130.2, 125.6, 85.0, 76.8, 31.1, 24.9, 24.9, 5.9.

^{11}B NMR (129 MHz, $CDCl_3$) δ= 29.03.

HRMS-(ESI+) for $C_{12}H_{19}BIO_4$ [M+H]$^+$: calculated 365.0345; found: 365.0355.

6-(Iodomethyl)-5-methyl-4-(4,4,5,5-tetramethyl-1,3,2-dioxaborolan-2-yl)-5,6-dihydro-2*H*-pyran-2-one (19)

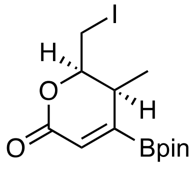

19

The product was purified by quenching with 15 mL of $Na_2S_2O_3$ and extraction (3 x Et_2O). The product was isolated as a yellow oil (67mg, 0.177 mmol, 98%) as a mixture of two diasteroisomers (63:37; *cis:trans).*

^{1}H NMR (400 MHz, $CDCl_3$) δ= *Major product:* 6.42 (s, 1H), 4.54 (ddd, *J* = 9.1, 6.4, 3.1 Hz, 1H), 3.37 (m, 1H), 3.19 (dd, *J* = 10.4, 8.6 Hz, 1H), 2.91 (m, 1H), 1.28 (s, 12H), 0.97 (d, *J* = 7.0 Hz, 3H). *Minor product:* 6.43 (s, 1H), 4.19 (dt, *J* = 6.6, 5.0 Hz, 1H), 3.42 – 3.35 (m, 2H), 2.95 – 2.86 (m, 1H), 1.29 (s, 12H), 1.20 (d, *J* = 7.1 Hz, 3H).

^{13}C NMR (100 MHz, $CDCl_3$) δ= *Major product:* 163.2, 128.9, 84.9, 79.8, 32.7, 25.0, 24.6, 10.6, 1.3. *Minor product:* 161.4, 129.1, 84.9, 82.1, 33.9, 24.9, 24.6, 17.7, 5.6.

^{11}B NMR (129 MHz, $CDCl_3$) δ= 29.29.

HRMS-(ESI+) for $C_{13}H_{21}BIO_4$ [M+H]$^+$: calculated 379.0572; found: 379.0582.

5-(Bromomethyl)-6-(iodomethyl)-4-(4,4,5,5-tetramethyl-1,3,2-dioxaborolan-2-yl)-5,6-dihydro-2*H*-pyran-2-one (20)

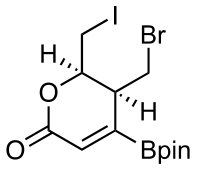

20

The product was purified by quenching with 15 mL of $Na_2S_2O_3$ and extraction (3 x Et_2O). The product was isolated as a yellow oil (106 mg, 0.232 mmol, 97%) as a mixture of two diasteroisomers (77:23; *cis:trans*).

^{1}H NMR (400 MHz, $CDCl_3$) δ= *Major product:* 6.58 (s, 1H), 4.53 (q, *J* = 5.4 Hz, 1H), 3.86 (dd, *J* = 10.8, 6.4 Hz, 1H), 3.47 (m, 1H), 3.44 (d, *J* = 5.1 Hz, 2H), 3.27 (tdd, *J* = 6.3, 3.3, 1.9 Hz, 1H), 1.31 (s, 12H). *Minor product:* 6.60 (s, 1H), 4.66 (ddd, *J* = 8.5, 6.7, 3.4 Hz, 1H), 3.47 (d, *J* = 3.3 Hz, 2H), 3.42 – 3.33 (m, 3H), 1.31 (s, 12H).

^{13}C NMR (100 MHz, $CDCl_3$) δ= *Major product:* 161.0, 131.7, 85.2, 78.0, 40.8, 31.8, 24.9, 24.6, 5.2. *Minor product:* 162.3, 131.2, 85.2, 79.5, 38.1, 27.4, 25.0, 24.6, 0.6.

^{11}B NMR (129 MHz, $CDCl_3$) δ= 29.95.

8-Iodo-4-(4,4,5,5-tetramethyl-1,3,2-dioxaborolan-2-yl)-4a,5,6,7,8,8a-hexahydro-2*H*-chromen-2-one (21)

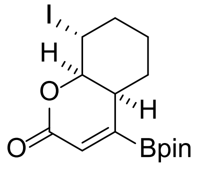

21

The product was purified by quenching with 15 mL of $Na_2S_2O_3$ and extraction (3 x Et_2O). The product was isolated as a yellow solid (88 mg, 0.218 mmol, 96%).

^{1}H NMR (400 MHz, $CDCl_3$) δ= 6.44 (s, 1H), 4.67 (dd, *J* = 3.0, 1.1 Hz, 1H), 4.58 (bs, 1H), 3.07 (ddd, *J* = 12.5, 4.2, 2.7 Hz, 1H), 1.97 – 1.90 (m, 2H), 1.84 – 1.75 (m, 2H), 1.67 – 1.60 (m, 1H), 1.30 (s, 6H), 1.28 (s, 6H), 1.19 – 1.11 (m, 1H).

^{13}C NMR (100 MHz, $CDCl_3$) δ= 163.2, 129.3, 84.8, 79.4, 32.1, 29.4, 28.7, 26.9, 25.0, 24.6, 20.9.

^{11}B NMR (129 MHz, CDCl$_3$) δ= 29.34. **Melting Point** = 124-124.4 ºC

HRMS-(ESI+) for $C_{15}H_{23}BIO_4$ [M+H]$^+$: calculated 405.0729; found: 405.0727.

8-Iodo-4,4-bis(4,4,5,5-tetramethyl-1,3,2-dioxaborolan-2-yl)octahydro-2*H*-chromen-2-one (29)

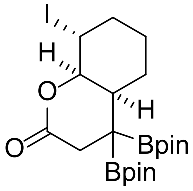

29

The product was purified by flash chromatography using as eluent a mixture of petroleum ether/ethyl acetate (100:1). The product was isolated as a pail yellowish oil (101 mg, 0.190 mmol, 58%).

^{1}H NMR (400 MHz, CDCl$_3$) δ = 4.56 (s, 2H), 2.93 (ddd, *J* = 13.0, 4.3, 2.2 Hz, 1H), 2.80 (dd, *J* = 19.5, 1.6 Hz, 1H), 2.69 (dd, *J* = 19.4, 1.6 Hz, 1H), 1.88 – 1.77 (m, 3H), 1.65 – 1.54 (m, 3H), 1.22 (s, 24H).

^{13}C NMR (100 MHz, CDCl$_3$) δ = 171.3, 84.3, 84.1, 82.8, 31.2, 30.1, 28.8, 28.3, 25.1, 24.8, 24.7, 24.5, 23.7, 21.5.

^{11}B NMR (129 MHz, CDCl$_3$) δ = 33.03.

HRMS-(ESI+) for $C_{21}H_{36}B_2IO_6$ [M+H]$^+$: calculated 533.1737; found: 533.1734.

Characterization data for coupled products

Ethyl (*E*)-6,6-difluoro-3-phenylhexa-2,5-dienoate (22)

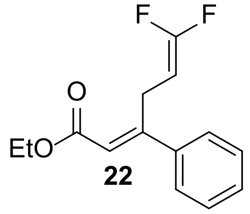

22

The product was purified by flash chromatography using as eluent a mixture of petroleum ether/ethyl acetate (100:1). The product was isolated as a pail yellowish oil (84 mg, 0.333 mmol, 87%).

^{1}H NMR (400 MHz, CDCl$_3$) δ= 7.45 – 7.40 (m, 2H), 7.40 – 7.36 (m, 3H), 6.11 (s, 1H), 4.32 – 4.14 (m, 3H), 3.84 – 3.76 (m, 2H), 1.32 (t, *J* = 7.1 Hz, 3H).

^{13}C NMR (100 MHz, CDCl$_3$) δ= 166.3, 156.6 (dd, $^1J_{C-F}$ = 288.9, 284.9 Hz), 140.4, 129.4, 128.8, 126.8, 118.5, 76.4 (dd, $^2J_{C-F}$ = 24.7, 19.6 Hz), 60.3, 24.1 (d, $^3J_{C-F}$ = 4.9 Hz), 14.42.

^{19}F NMR (377 MHz, CDCl$_3$) δ= -88.41 (d, J = 44.4 Hz), -89.70 (dd, J = 44.4, 25.0 Hz).

HRMS-(ESI+) for $C_{14}H_{15}F_2O_2$ [M+H]$^+$: calculated 253.1035; found: 253.1025.

6-(Difluoroiodomethyl)-4-phenyl-5,6-dihydro-2*H*-pyran-2-one (23)

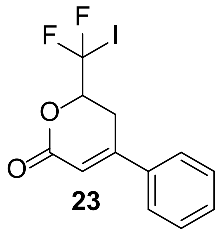

23

The product was purified by flash chromatography using as eluent a mixture of petroleum ether/ethyl acetate (100:3). The product was isolated as a pail yellowish oil (24 mg, 0.069 mmol, 24%).

^{1}H NMR (400 MHz, CDCl$_3$) δ = 7.51 – 7.39 (m, 5H), 6.43 (d, J = 1.9 Hz, 1H), 4.90 (dddd, J = 10.8, 5.9, 4.8, 1.0 Hz, 1H), 3.72 (ddt, J = 19.4, 4.9, 1.8 Hz, 1H), 3.39 (ddd, J = 19.4, 5.9, 2.4 Hz, 1H).

^{13}C NMR (100 MHz, CDCl$_3$) δ = 157.9, 154.3, 140.3, 130.6, 129.2, 126.6, 120.3 (dd, $^1J_{C\text{-}F}$ = 263.9, 259.2 Hz), 39.6 (t, $^3J_{C\text{-}F}$ = 4.9 Hz), 18.3 (t, $^2J_{C\text{-}F}$ = 34.7 Hz).

^{19}F NMR (377 MHz, CDCl3) δ = -64.71 (d, J = 159.2 Hz), -67.36 (dd, J = 159.2, 10.6 Hz).

HRMS-(ESI+) for $C_{12}H_{10}F_2IO_2$ [M+H]$^+$: calculated 350.9688; found: 350.9691.

Ethyl (*E*)-5-methyl-3-phenylhexa-2,5-dienoate (24)

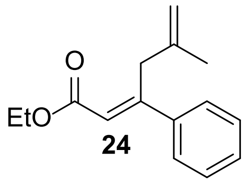

24

The product was purified by flash chromatography using as eluent a mixture of petroleum ether/ethyl acetate (100:1). The product was isolated as a pail yellowish oil (61 mg, 0.265 mmol, 68%)

^{1}H NMR (400 MHz, CDCl$_3$) δ= 7.50 – 7.45 (m, 2H), 7.37 – 7.33 (m, 3H), 6.23 (s, 1H), 4.75 (s, 1H), 4.65 (s, 1H), 4.21 (q, J = 7.1 Hz, 2H), 3.84 (s, 2H), 1.76 (s, 3H), 1.31 (t, J = 7.1 Hz, 3H).

^{13}C NMR (100 MHz, CDCl$_3$) δ= 166.4, 156.8, 143.1, 141.3, 129.0, 128.5, 126.9, 118.9, 111.6, 60.0, 38.8, 23.1, 14.4.

HRMS-(ESI+) for $C_{15}H_{19}O_2$ [M+H]$^+$: calculated 231.1380; found: 231.1381

6-(Iodomethyl)-6-methyl-4-phenyl-5,6-dihydro-2*H*-pyran-2-one (25)

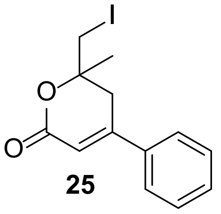

25

The product was purified by flash chromatography using as eluent a mixture of petroleum ether/ethyl acetate (100:3). The product was isolated as a pail yellowish oil (50 mg, 0.152 mmol, 61%).

^{1}H NMR (400 MHz, $CDCl_3$) δ= 7.58 – 7.54 (m, 2H), 7.49 – 7.44 (m, 3H), 6.36 (t, *J* = 1.5 Hz, 1H), 3.60 (d, *J* = 10.5 Hz, 1H), 3.45 (d, *J* = 10.4 Hz, 1H), 3.28 (dd, *J* = 17.8, 1.5 Hz, 1H), 2.86 (dd, *J* = 17.8, 1.4 Hz, 1H), 1.70 (s, 3H).

^{13}C NMR (100 MHz, $CDCl_3$) δ= 163.9, 153.2, 137.2, 131.0, 129.2, 126.6, 79.6, 35.8, 25.8, 12.9.

HRMS-(ESI+) for $C_{13}H_{14}IO_2$ [M+H]$^+$: calculated 329.0033; found: 329.0045.

6-(Iodomethyl)-5-methyl-4-phenyl-5,6-dihydro-2*H*-pyran-2-one (26)

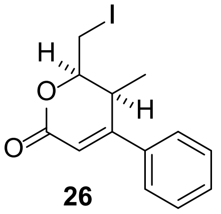

26

The product was purified by flash chromatography using as eluent a mixture of petroleum ether/ethyl acetate (100:3). The product was isolated as a pail yellowish oil (26 mg, 0.079 mmol, 48%).

^{1}H NMR (400 MHz, $CDCl_3$) δ= 7.61 – 7.54 (m, 2H), 7.49 – 7.44 (m, 3H), 6.30 (s, 1H), 4.71 (ddd, *J* = 9.6, 5.8, 2.9 Hz, 1H), 3.51 (dd, *J* = 10.3, 5.8 Hz, 1H), 3.40 (qd, *J* = 7.1, 2.9 Hz, 1H), 3.36 – 3.27 (m, 1H), 1.10 (d, *J* = 7.1 Hz, 3H).

^{13}C NMR (100 MHz, $CDCl_3$) δ= 164.8, 161.3, 134.8, 131.1, 129.3, 126.7, 113.6, 79.4, 33.3, 10.5, 1.2.

HRMS-(ESI+) for $C_{13}H_{14}IO_2$ [M+H]$^+$: calculated 329.0033; found: 329.0034

8-Iodo-4-phenyl-4a,5,6,7,8,8a-hexahydro-2*H*-chromen-2-one (28)

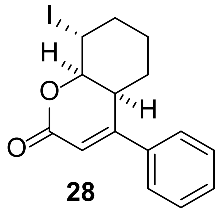

28

The product was purified by flash chromatography using as eluent a mixture of petroleum ether/ethyl acetate (100:3). The product was isolated as a pail yellow solid (55 mg, 0.155 mmol, 90%).

^{1}H NMR (400 MHz, $CDCl_3$) δ= 7.64 – 7.53 (m, 2H), 7.52 – 7.43 (m, 3H), 6.29 (s, 1H), 4.81 – 4.75 (m, 1H), 4.76 – 4.70 (m, 1H), 3.57 (ddd, *J* = 12.5, 4.4, 2.7 Hz, 1H), 2.07 – 1.95 (m, 2H), 1.92 – 1.77 (m, 2H), 1.71 – 1.63 (m, 1H), 1.50 – 1.37 (m, 1H).

^{13}C NMR (100 MHz, $CDCl_3$) δ= 164.8, 161.0, 134.8, 131.0, 129.3, 126.6, 113.7, 79.1, 33.2, 29.4, 28.9, 27.1, 20.9.

HRMS-(ESI+) for $C_{15}H_{16}IO_2$ [M+H]$^+$: calculated 355.0190; found: 355.0196

Characterization data for α–pyrone 27

5,6-Dimethyl-4-phenyl-2*H*-pyran-2-one (27)[2]

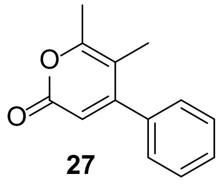

27

The product was purified by flash chromatography using as eluent a mixture of petroleum ether/ethyl acetate (100:3). The product was isolated as a pail yellowish oil (7 mg, 0.035 mmol, 25%).

^{1}H NMR (400 MHz, $CDCl_3$) δ= 7.47 – 7.41 (m, 3H), 7.27 – 7.21 (m, 2H), 6.08 (s, 1H), 2.31 (s, 3H), 1.85 (s, 3H).

^{13}C NMR (100 MHz, $CDCl_3$) δ= 162.8, 160.3, 158.4, 137.5, 129.0, 128.6, 127.7, 112.3, 110.3, 18.2, 14.0.

HRMS-(ESI+) for $C_{13}H_{13}O_2$ [M+H]$^+$: calculated 201.0910; found: 201.0916

^{1}H, ^{13}C, ^{11}B Spectra for borylated (*Z*)-skipped dienoates

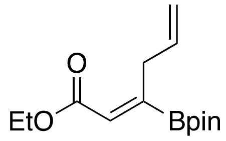

2a

^{1}H NMR (400 MHz, $CDCl_3$)

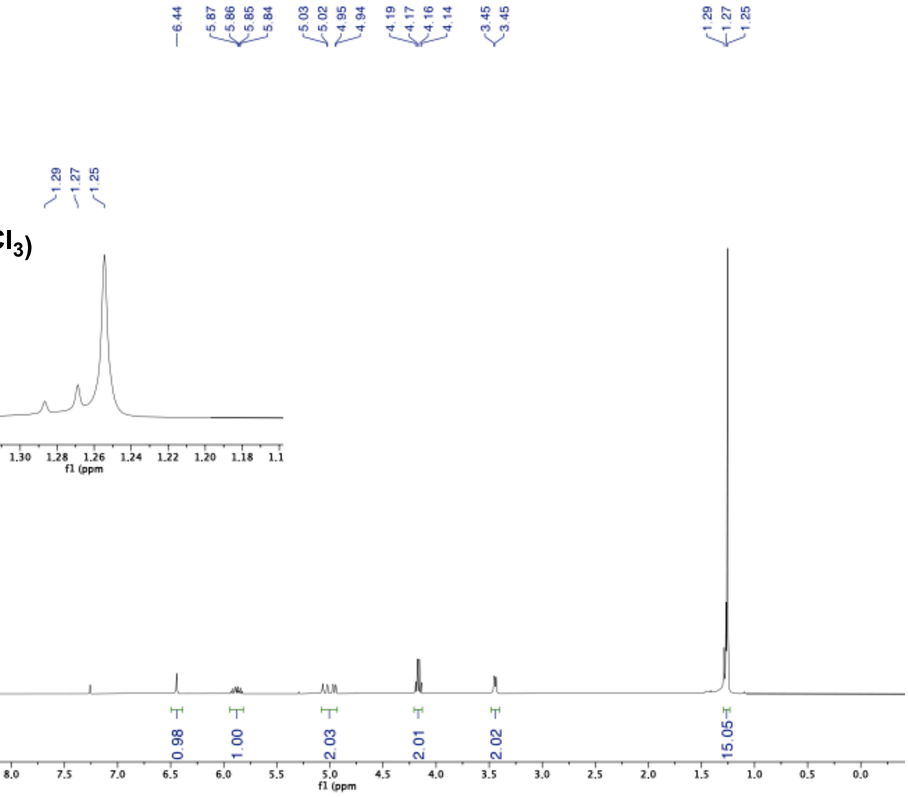

1-D NMR NOE EXPERIMENT

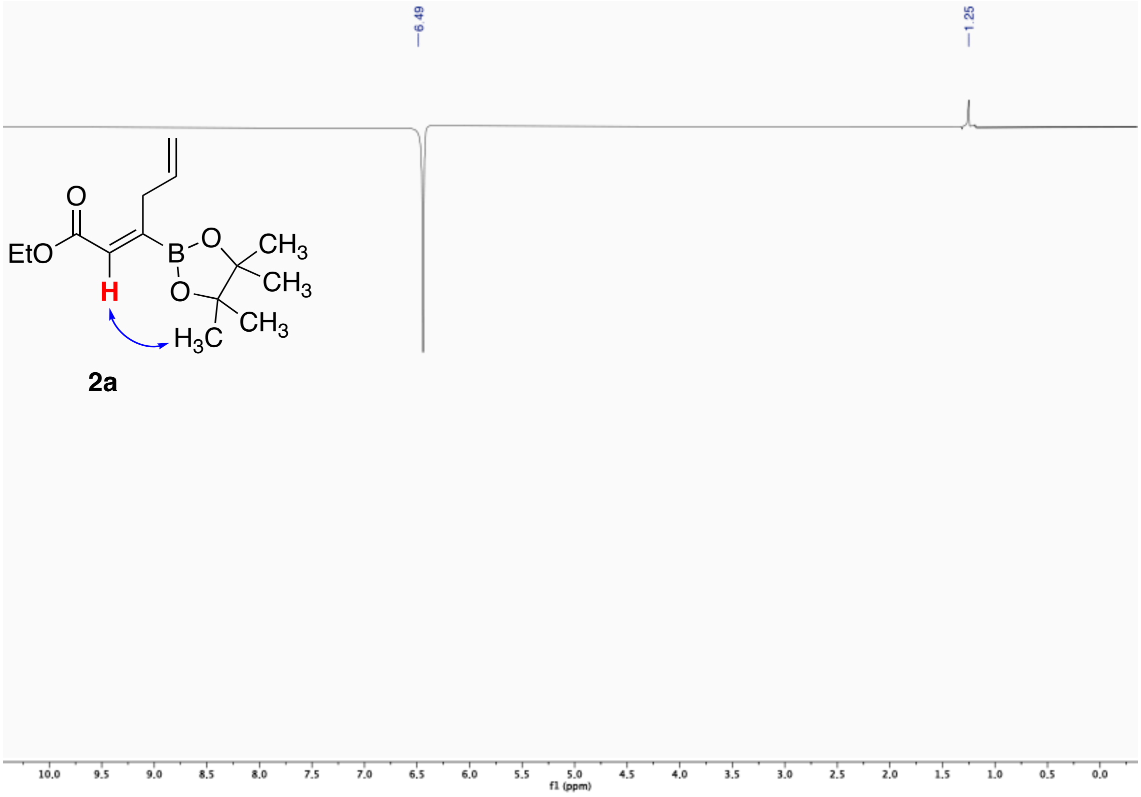

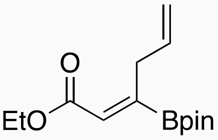

2a

^{13}C NMR (100 MHz, $CDCl_3$)

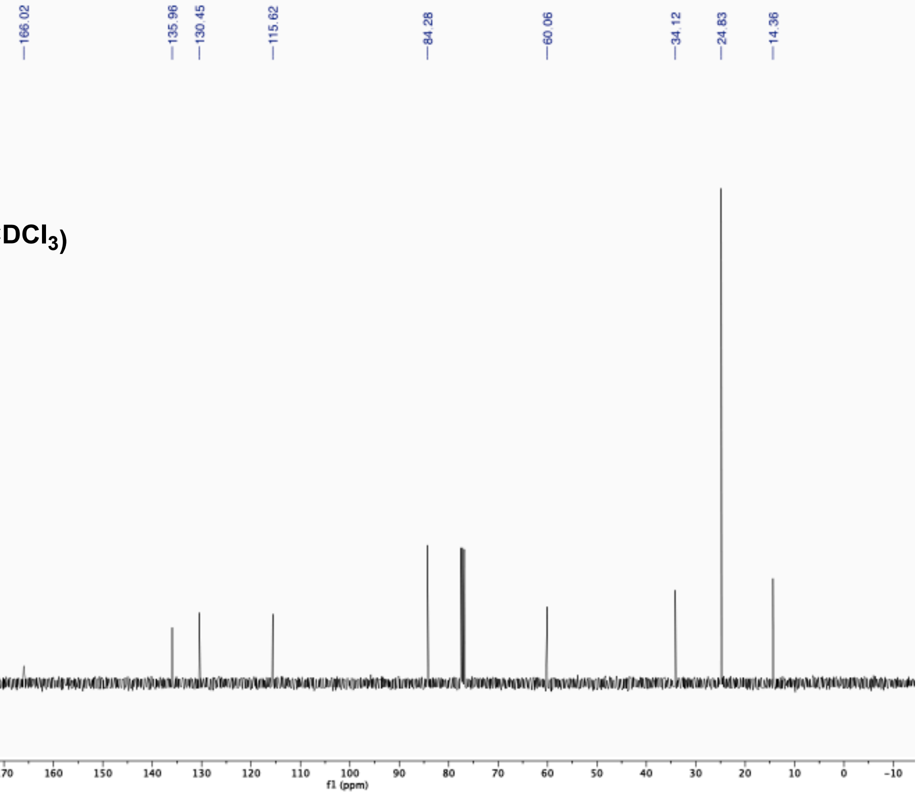

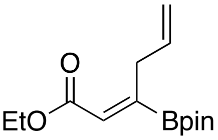

2a

^{11}B NMR (129 MHz, $CDCl_3$)

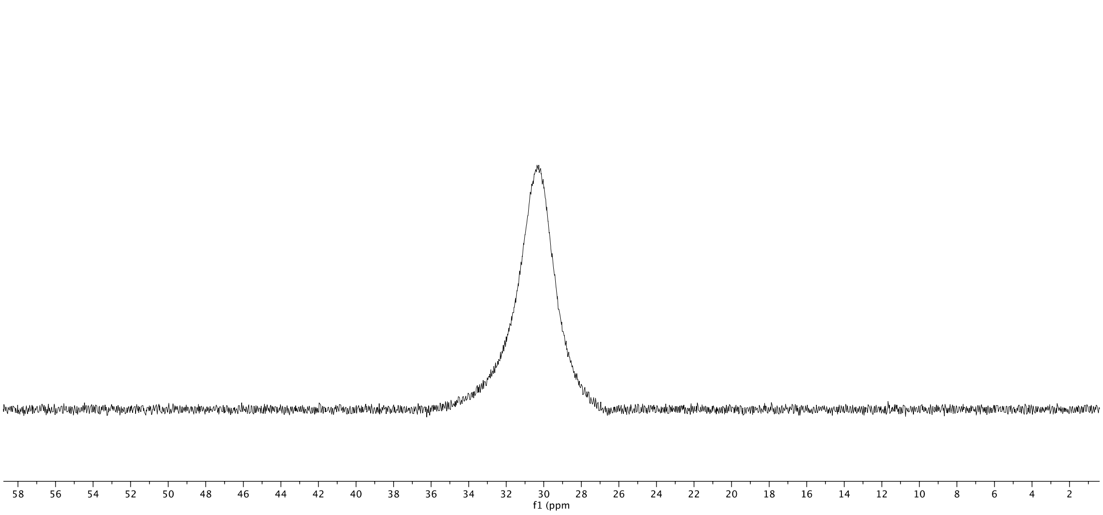

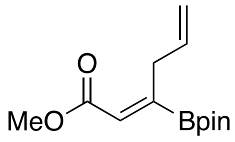

2b

^{1}H NMR (400 MHz, CDCl$_3$)

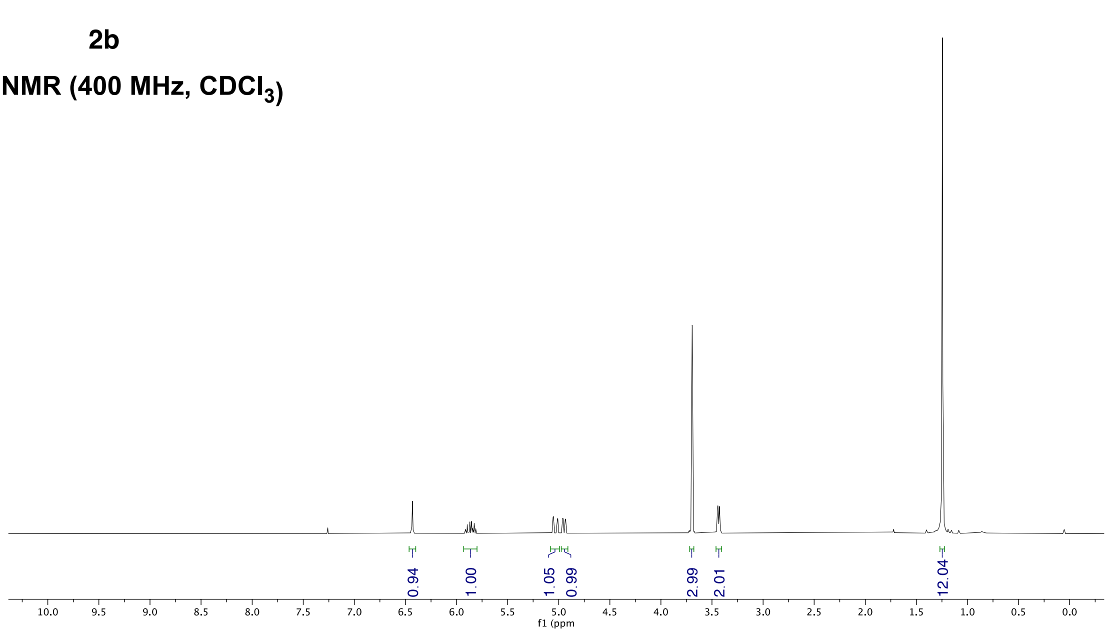

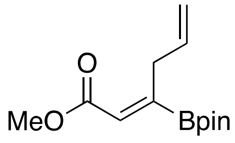

2b

^{13}C NMR (100 MHz, CDCl$_3$)

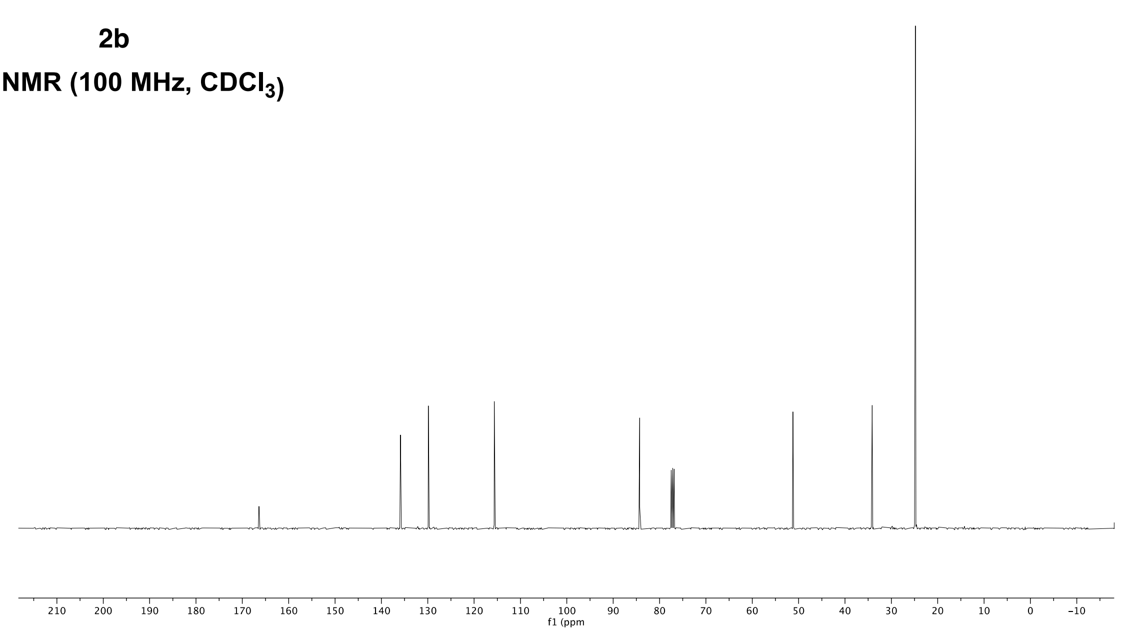

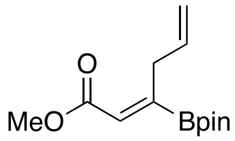

2b

^{11}B NMR (129 MHz, CDCl$_3$)

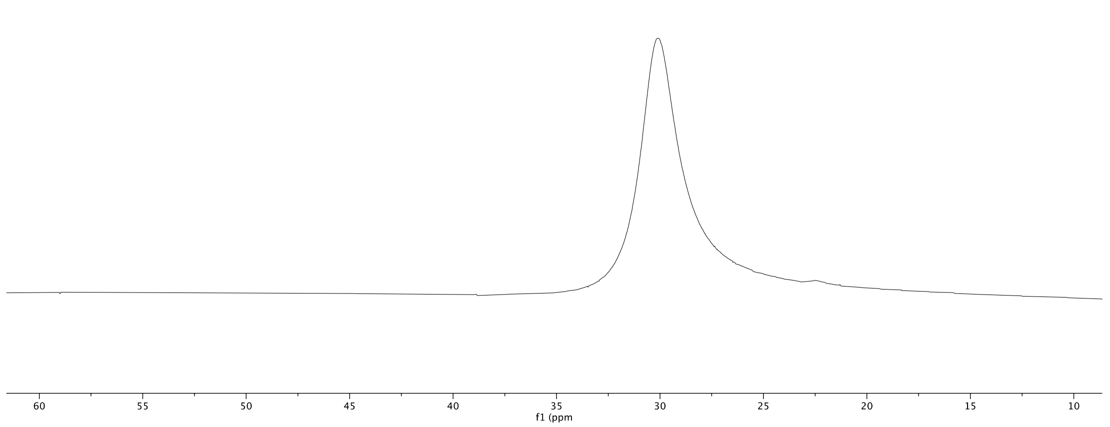

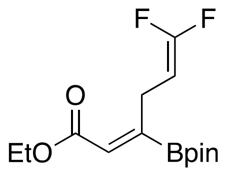

4a

^{1}H NMR (400 MHz, CDCl$_3$)

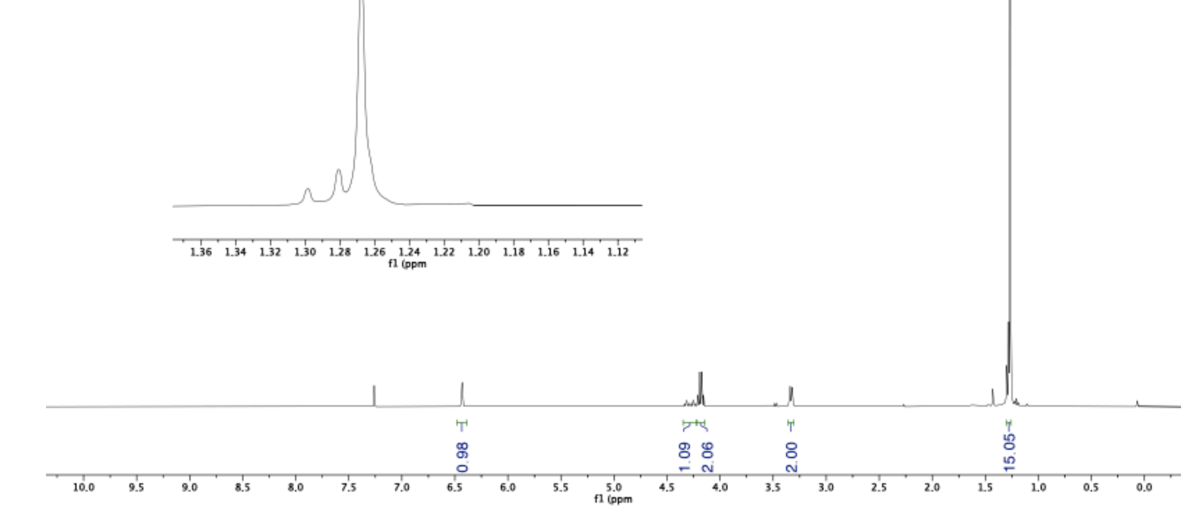

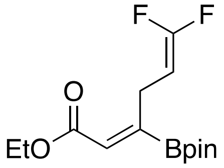

4a

^{13}C NMR (100 MHz, CDCl$_3$)

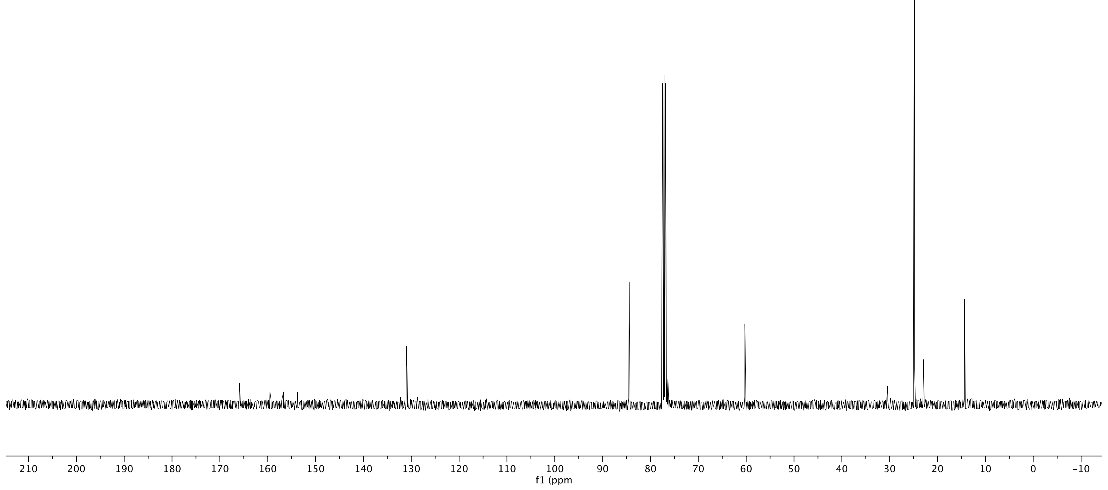

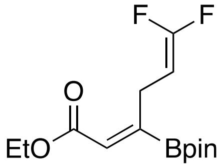

4a

^{11}B NMR (129 MHz, CDCl$_3$)

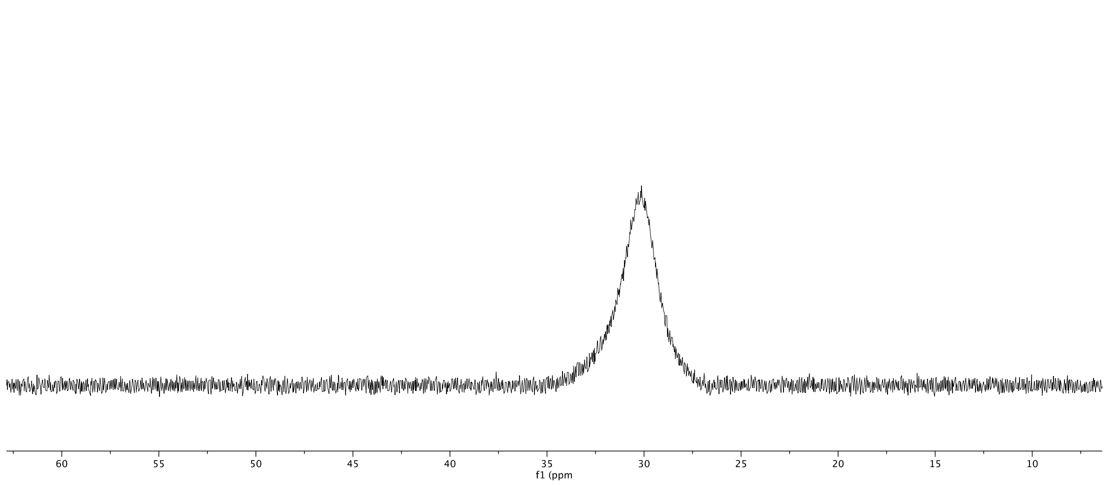

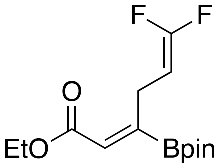

4a

^{19}F NMR (377 MHz, CDCl$_3$)

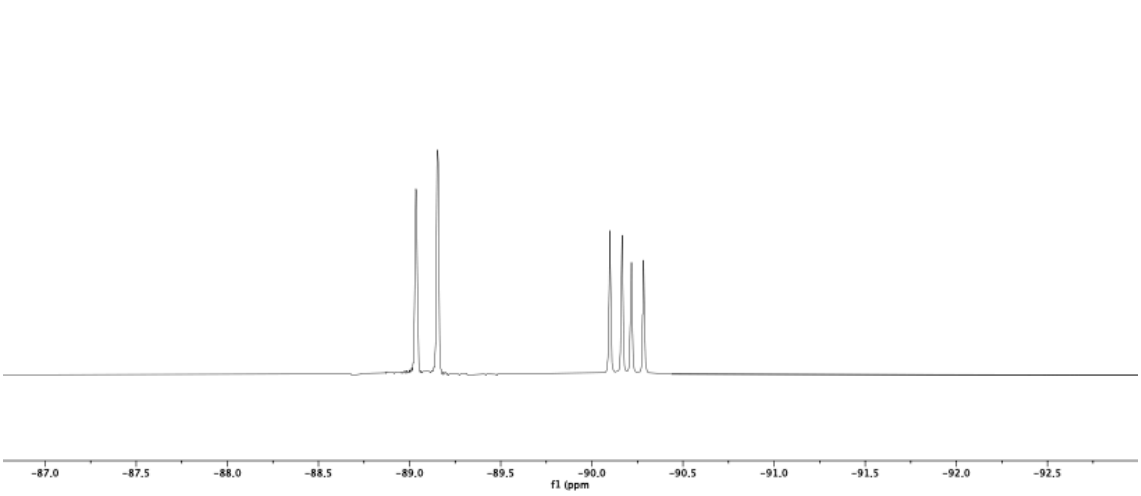

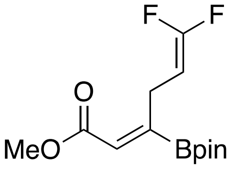

4b

^{1}H NMR (400 MHz, CDCl$_3$)

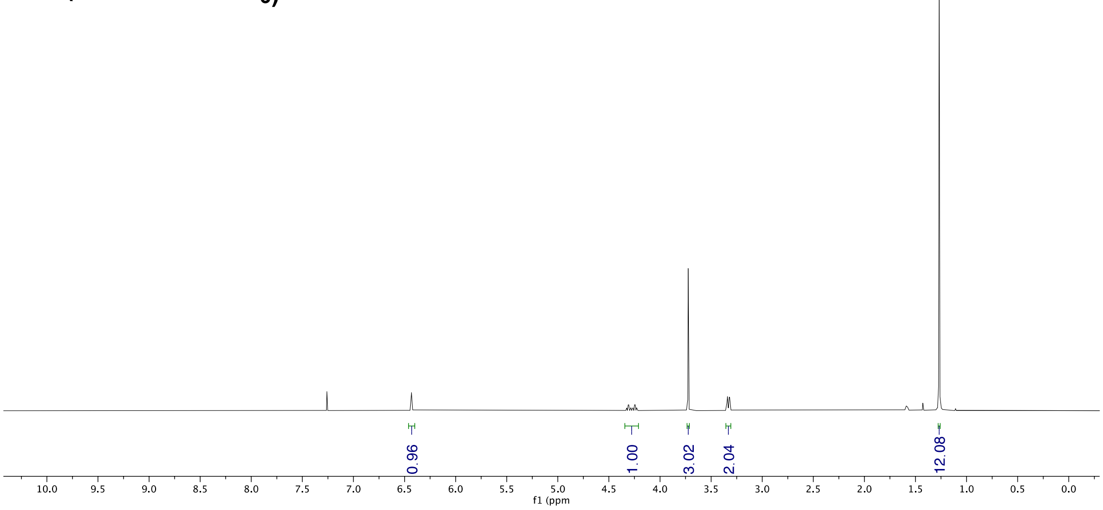

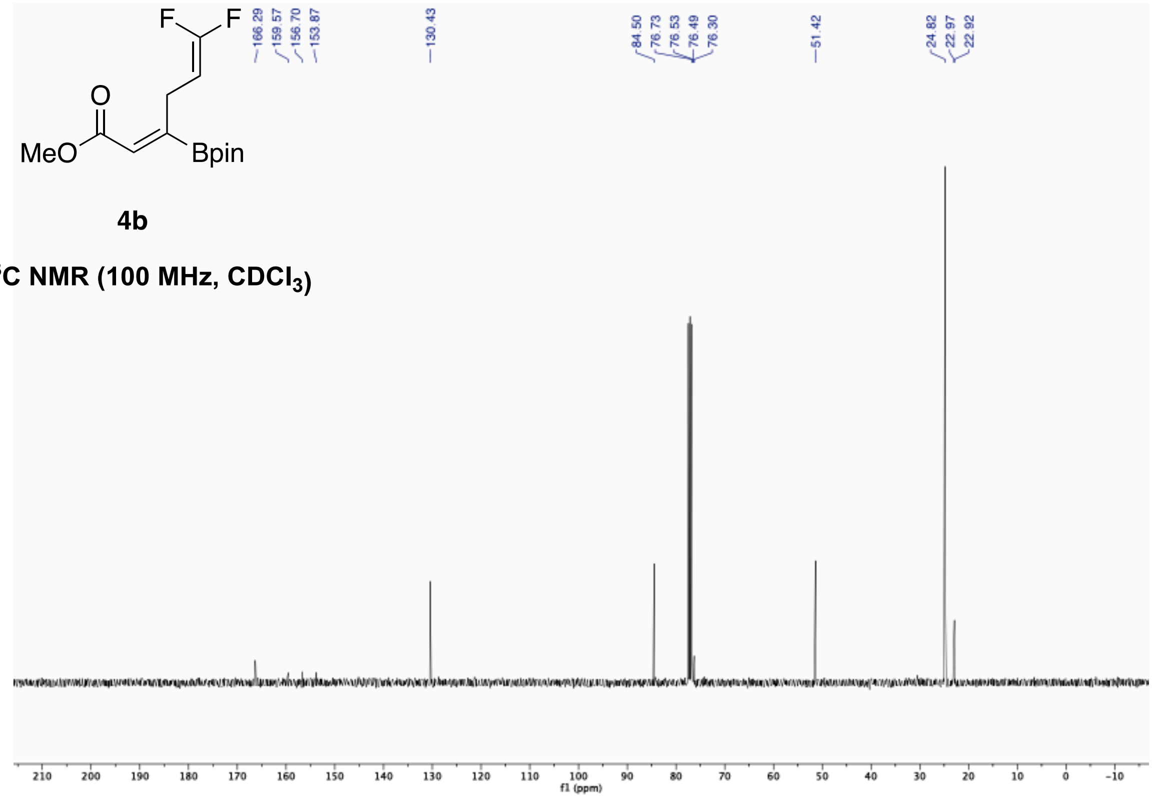

4b

^{13}C NMR (100 MHz, $CDCl_3$)

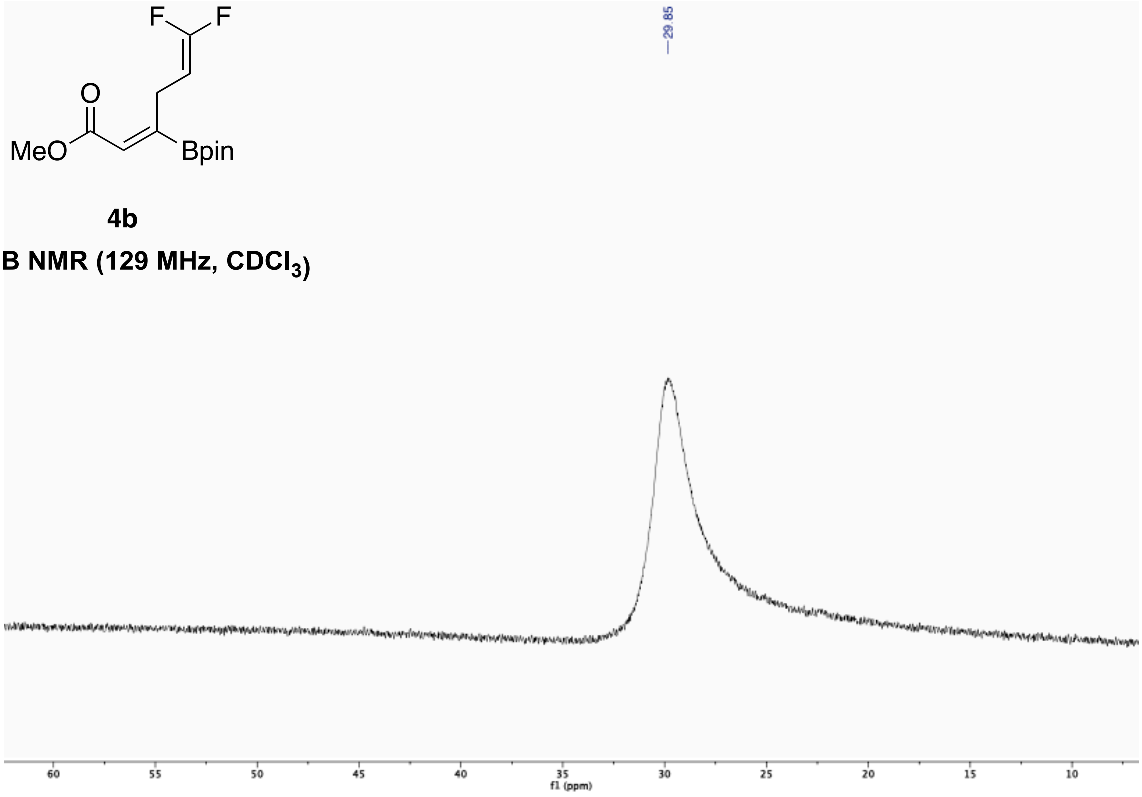

4b

^{11}B NMR (129 MHz, $CDCl_3$)

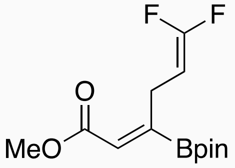

4b

^{19}F NMR (377 MHz, $CDCl_3$)

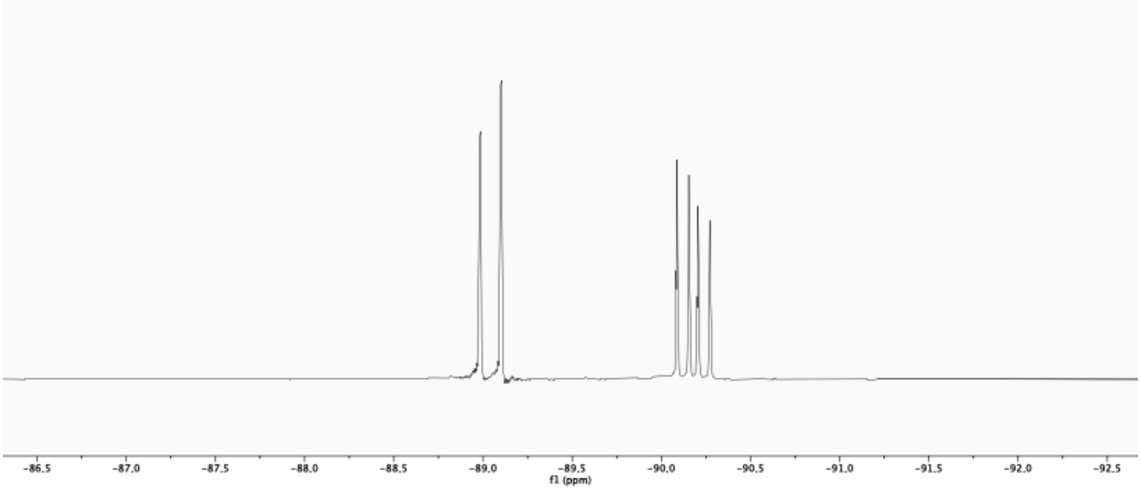

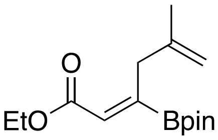

5a

^{1}H NMR (400 MHz, $CDCl_3$)

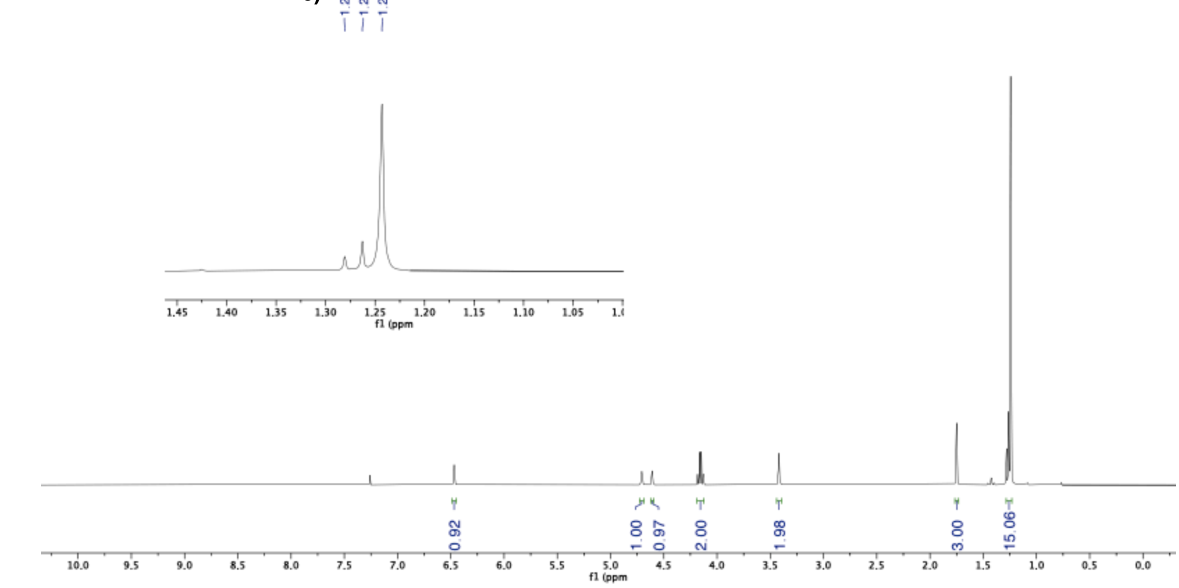

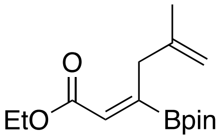

5a

^{13}C NMR (100 MHz, CDCl$_3$)

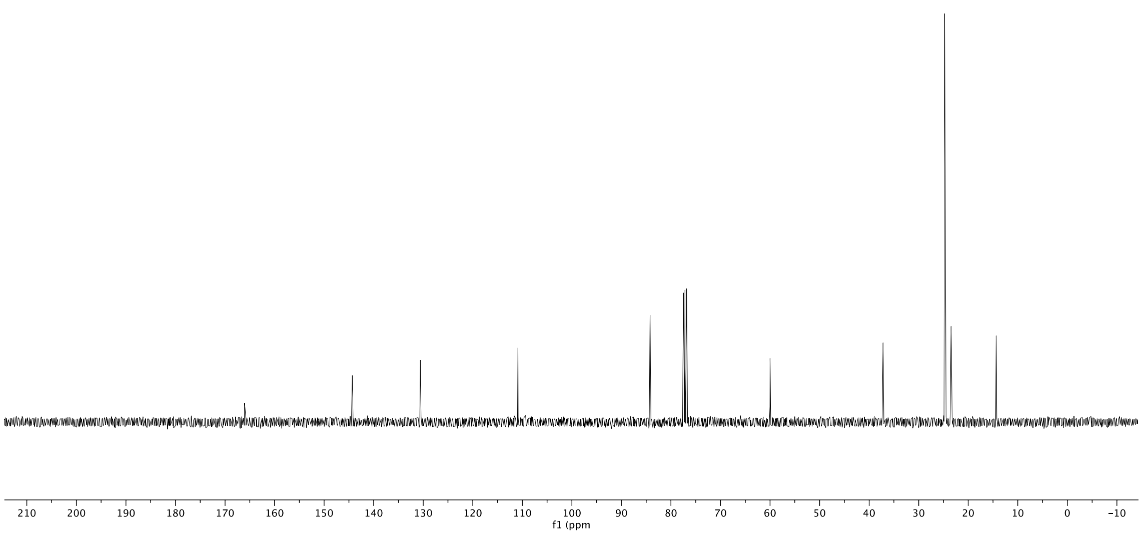

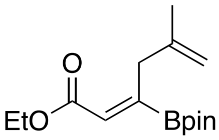

5a

^{11}B NMR (129 MHz, CDCl$_3$)

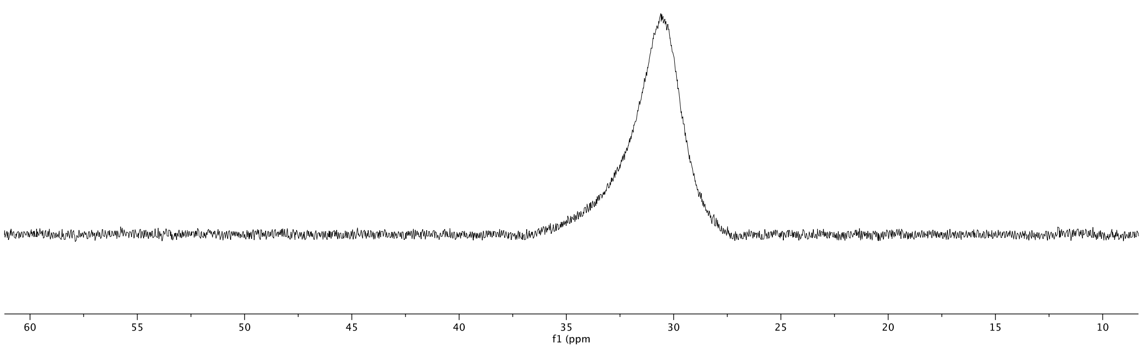

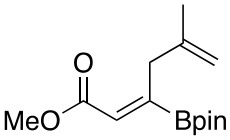

5b

^{1}H NMR (400 MHz, CDCl$_3$)

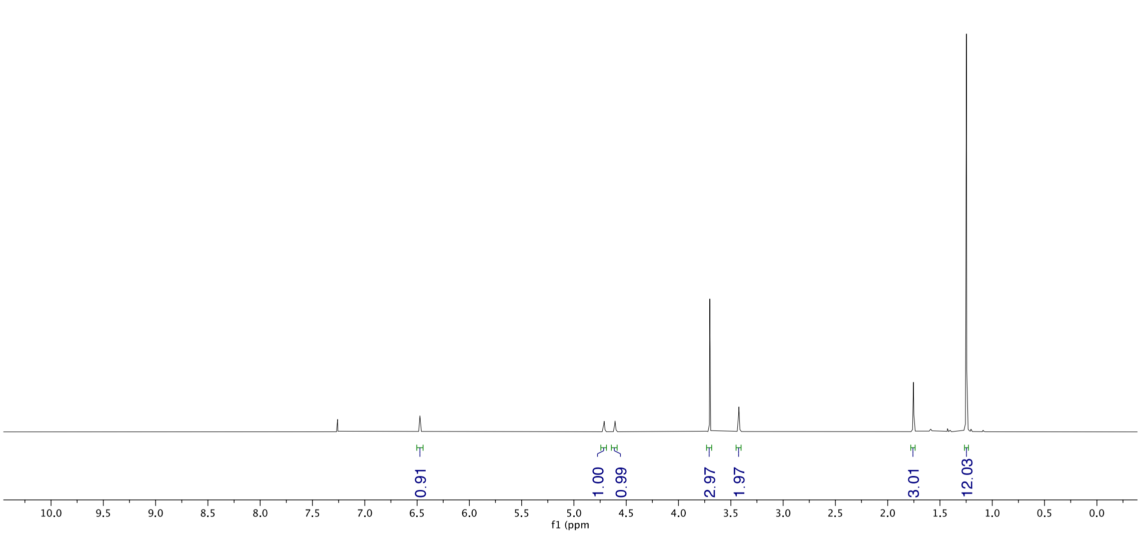

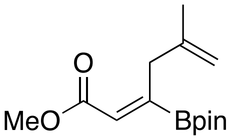

5b

^{13}C NMR (100 MHz, CDCl$_3$)

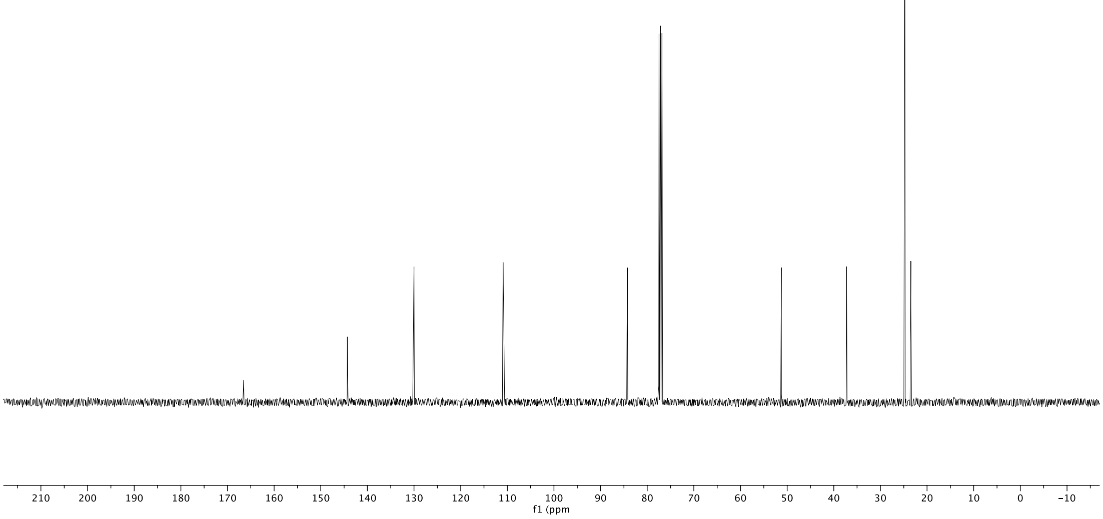

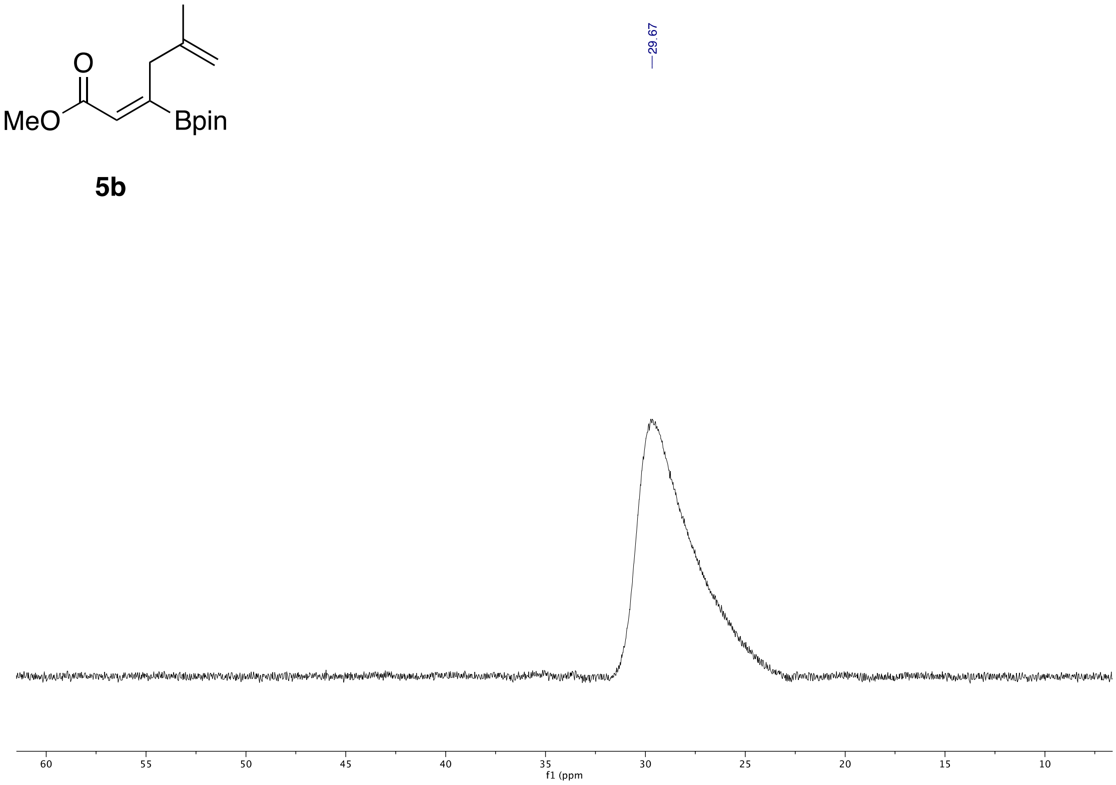

5b

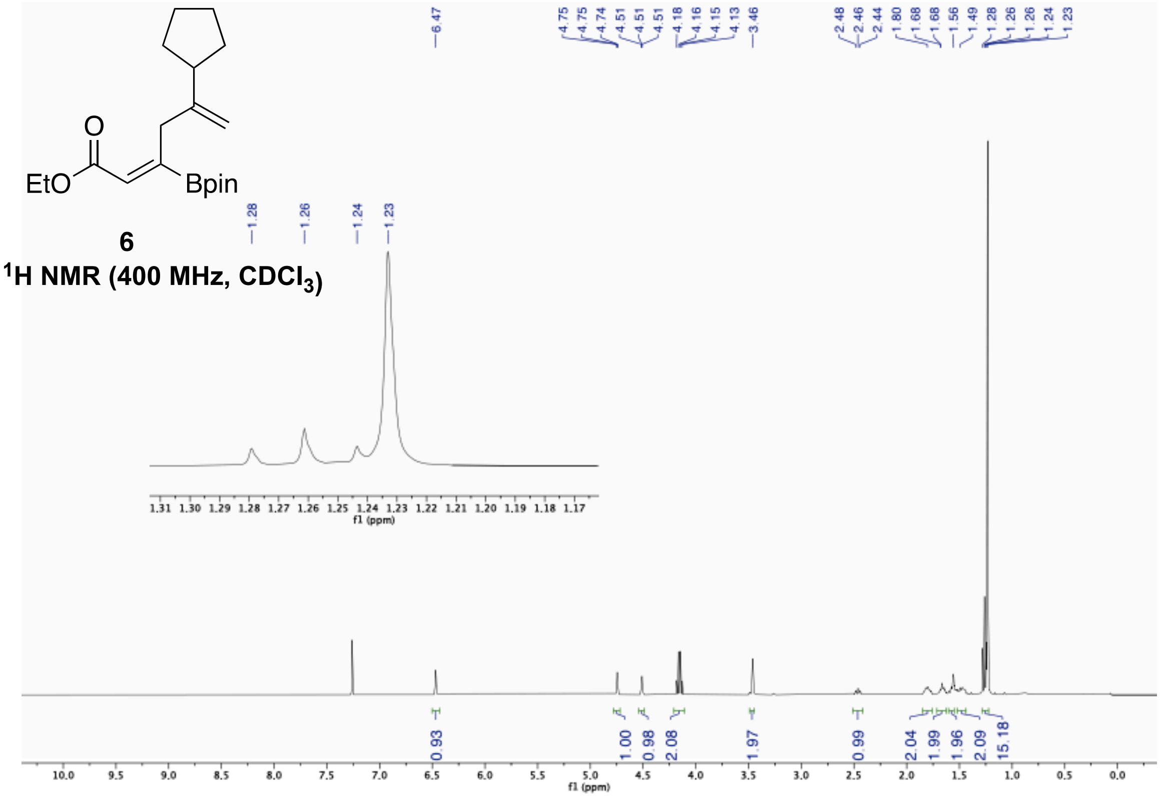

6

^{1}H NMR (400 MHz, $CDCl_3$)

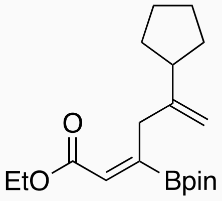

6

^{13}C NMR (100 MHz, CDCl$_3$)

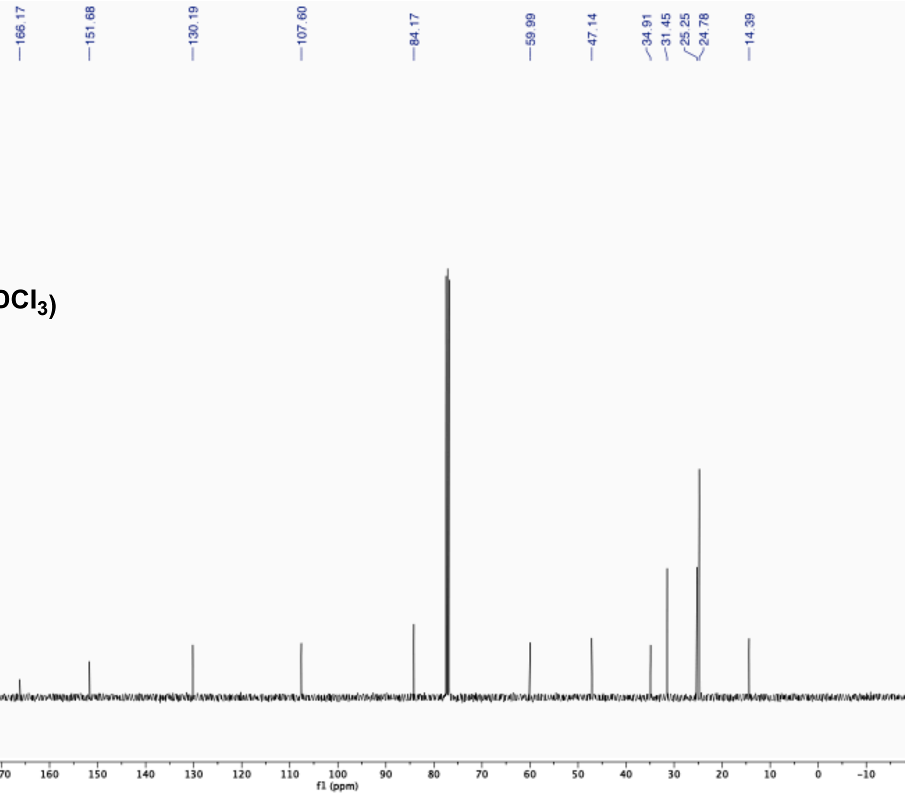

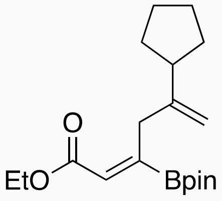

6

^{11}B NMR (129 MHz, CDCl$_3$)

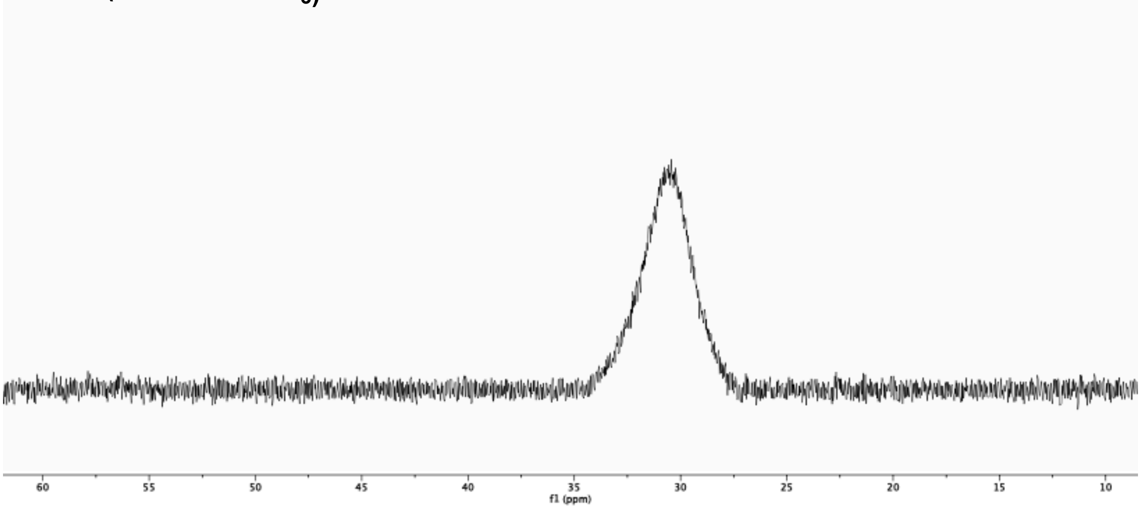

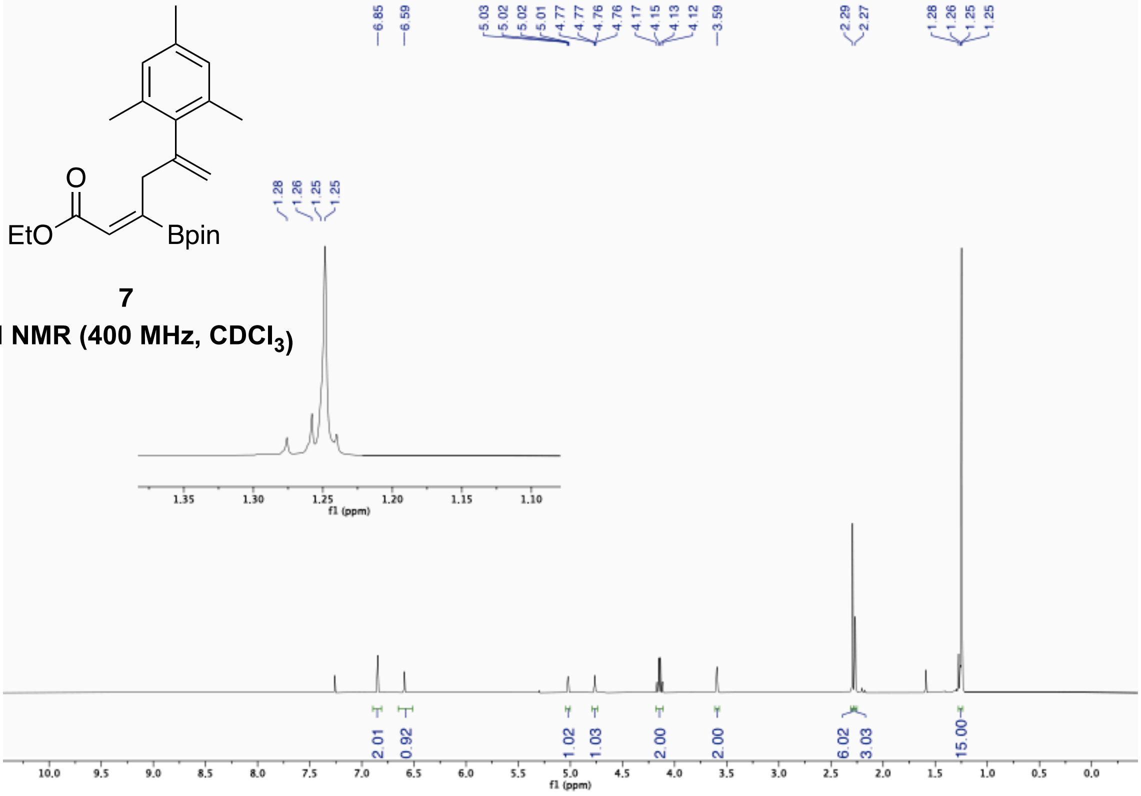

7

^{1}H NMR (400 MHz, $CDCl_3$)

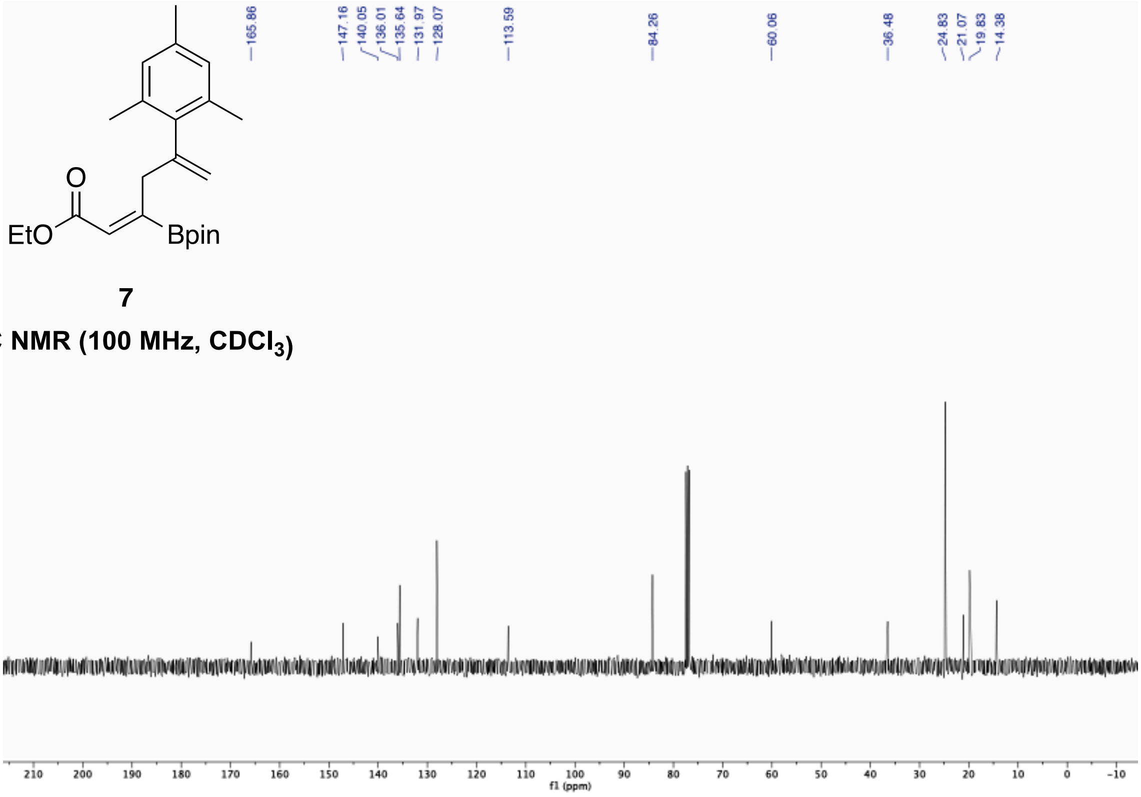

7

^{13}C NMR (100 MHz, $CDCl_3$)

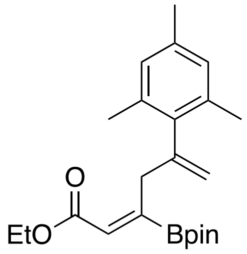

7

^{11}B NMR (129 MHz, $CDCl_3$)

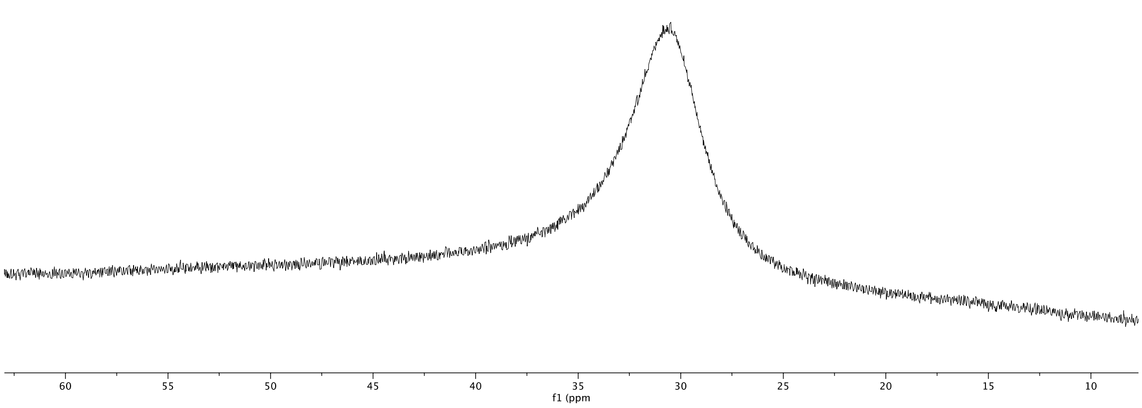

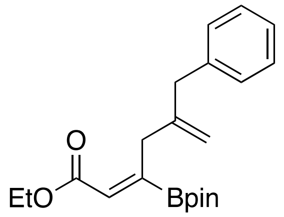

8

^{1}H NMR (400 MHz, $CDCl_3$)

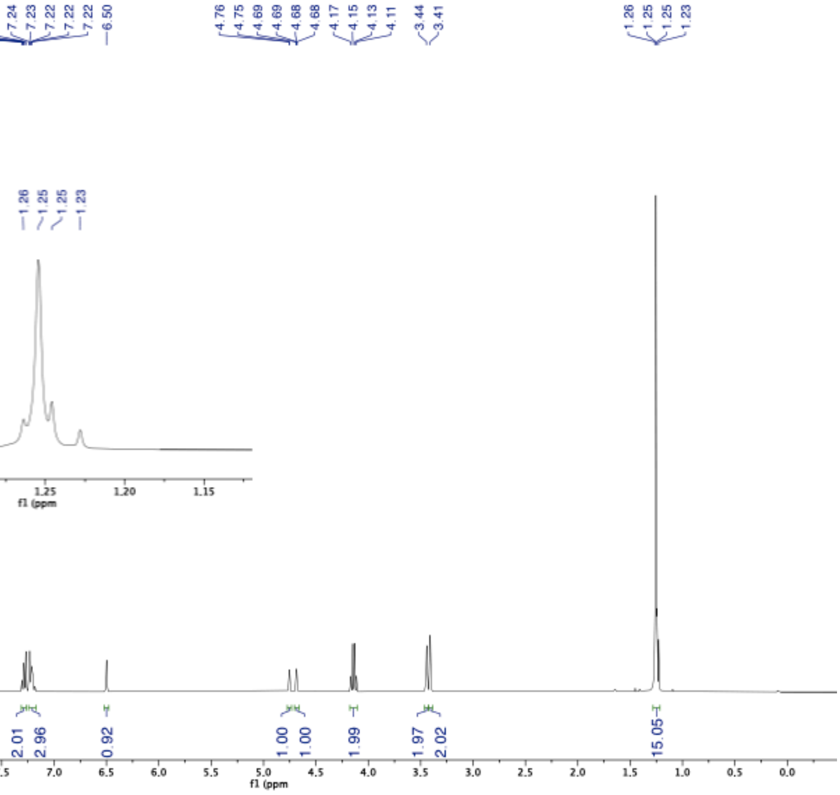

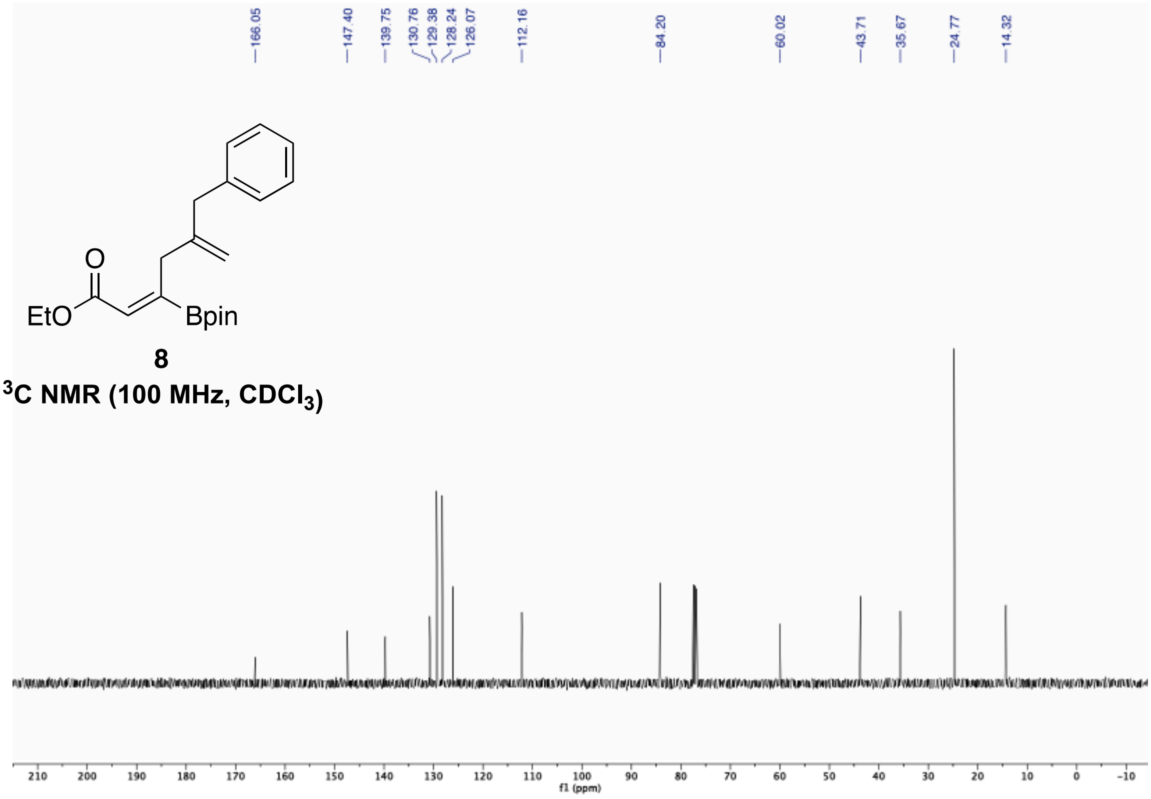

8

^{13}C NMR (100 MHz, $CDCl_3$)

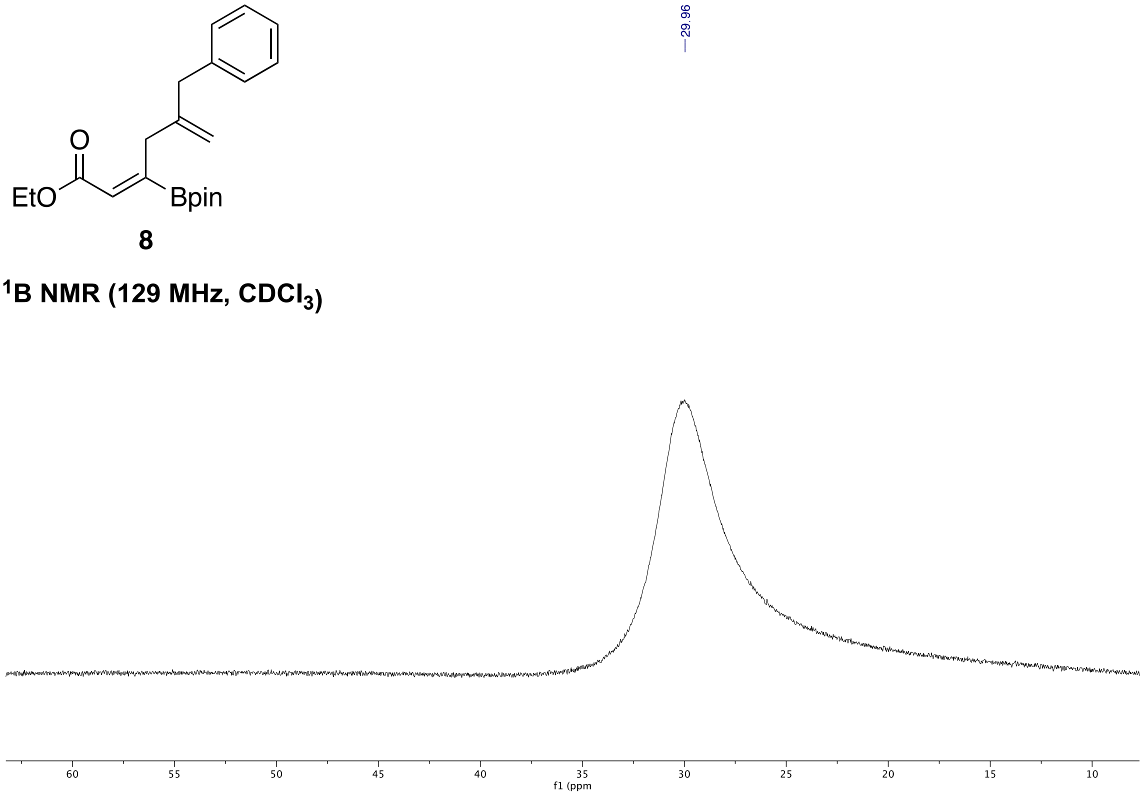

8

^{11}B NMR (129 MHz, $CDCl_3$)

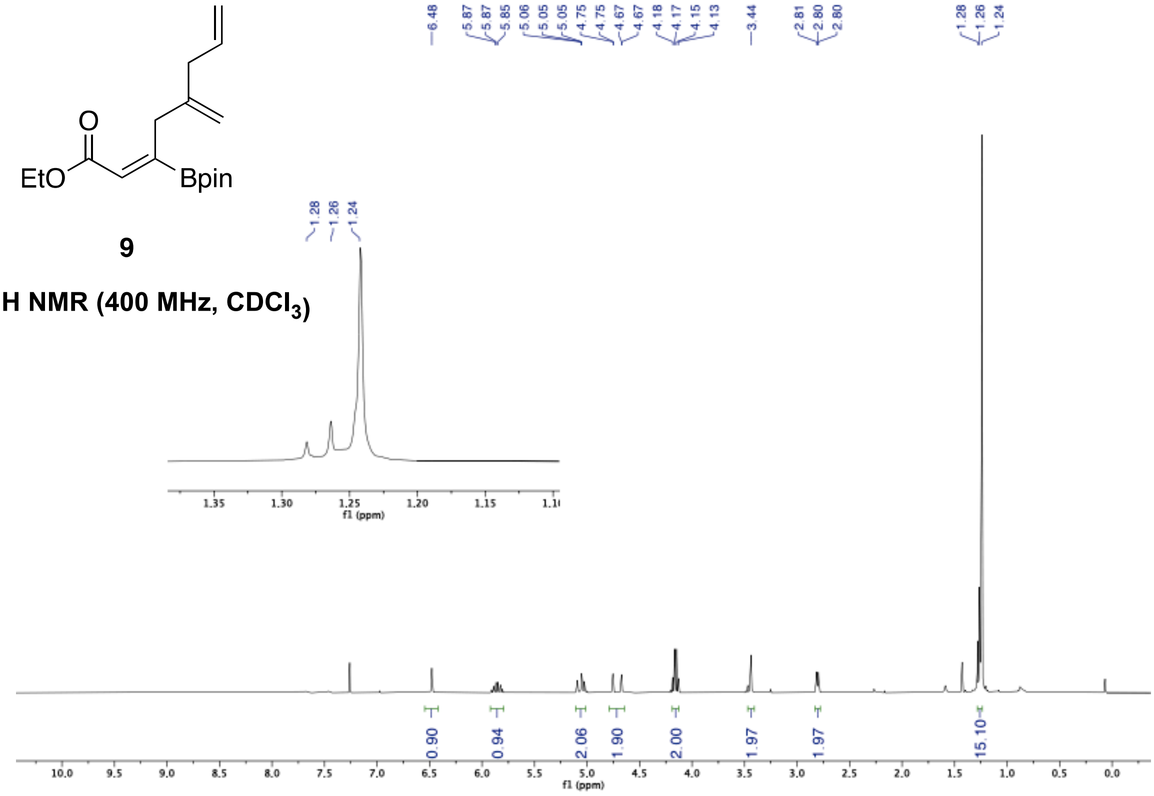

9

^{1}H NMR (400 MHz, $CDCl_3$)

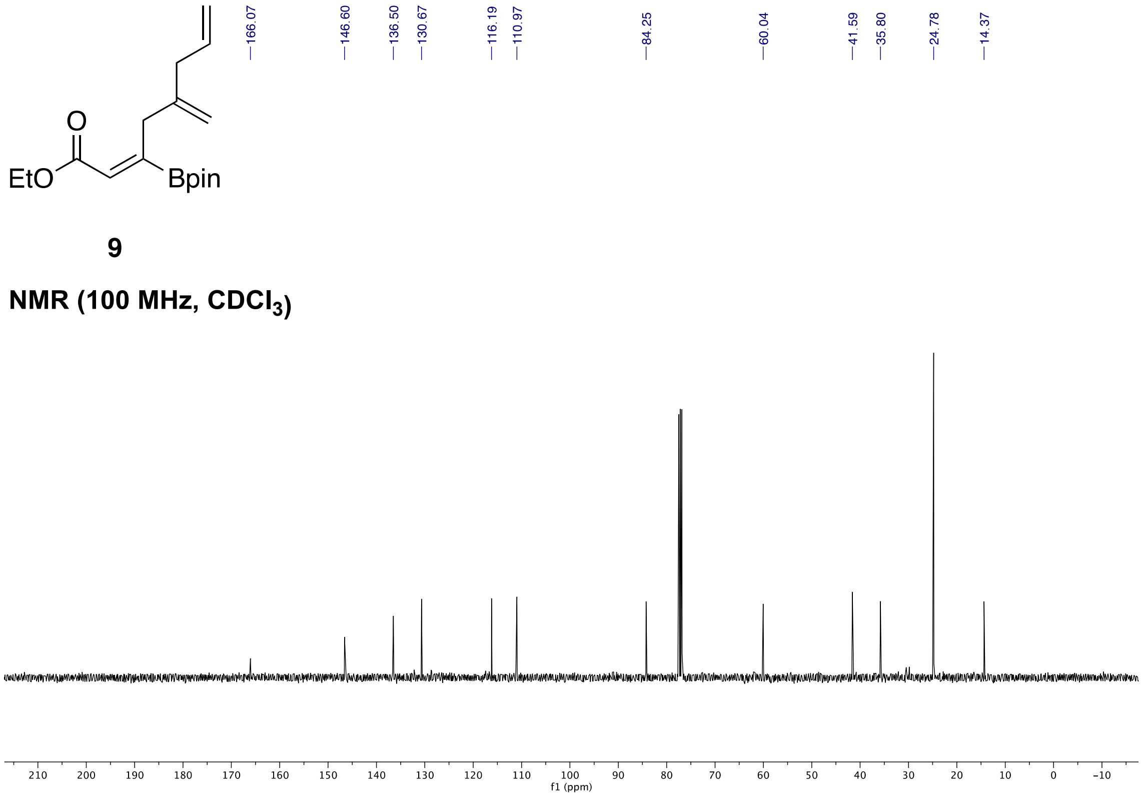

9

^{13}C NMR (100 MHz, $CDCl_3$)

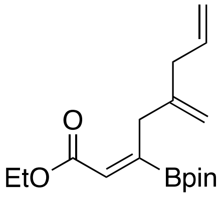

9

^{11}B NMR (129 MHz, CDCl$_3$)

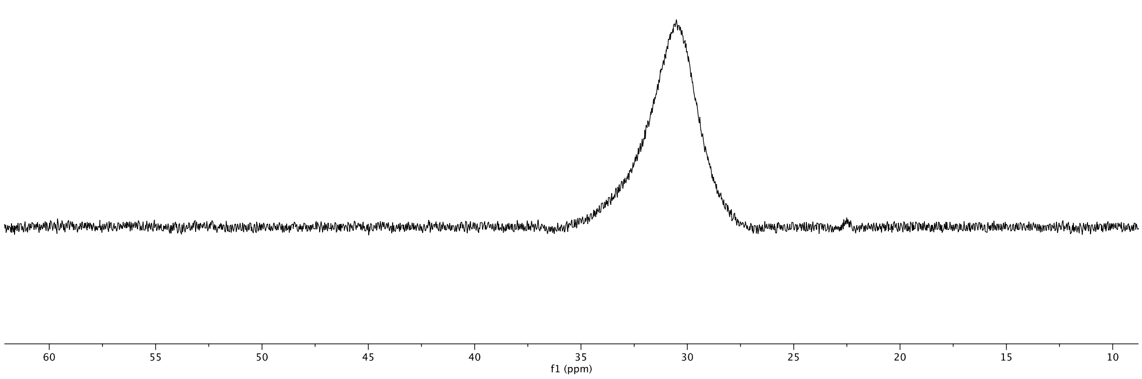

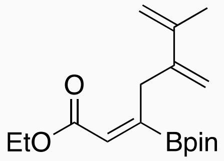

10

^{1}H NMR (400 MHz, CDCl$_3$)

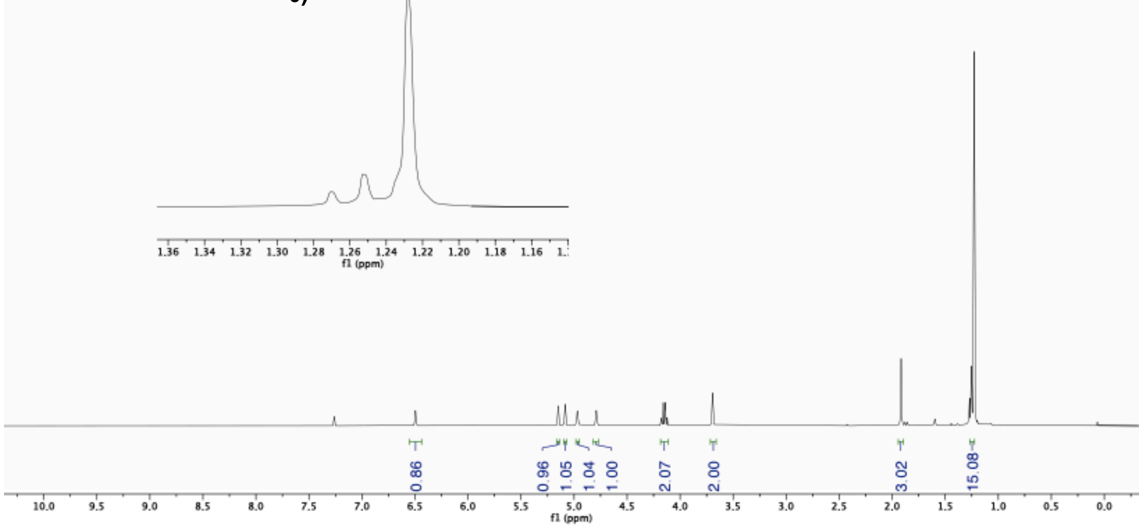

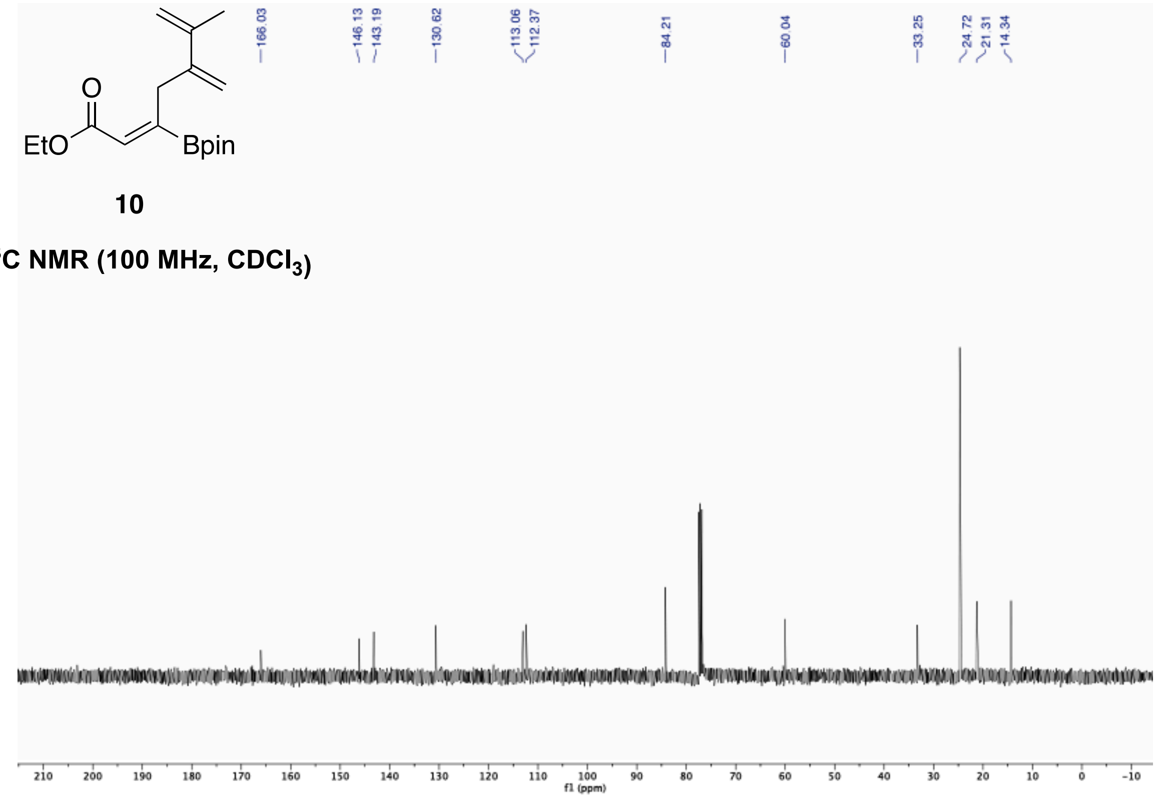

10

^{13}C NMR (100 MHz, $CDCl_3$)

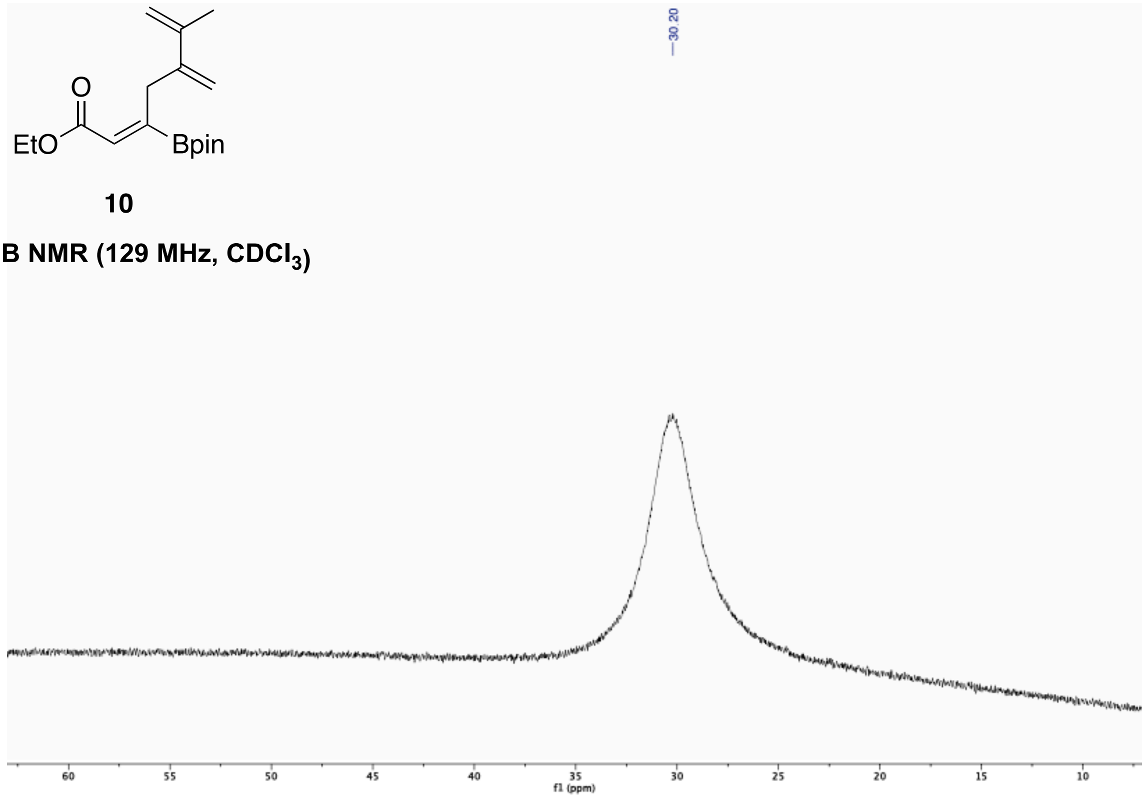

10

^{11}B NMR (129 MHz, $CDCl_3$)

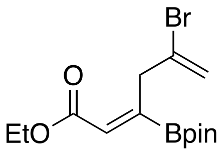

11

^{1}H NMR (400 MHz, $CDCl_3$)

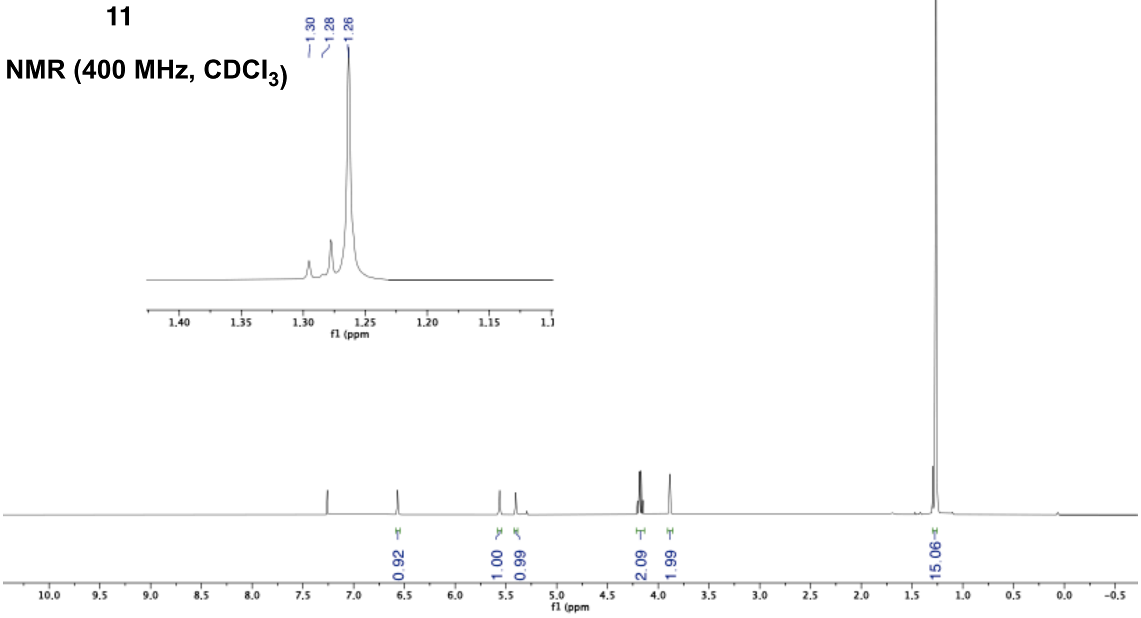

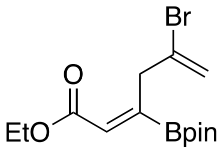

11

^{13}C NMR (100 MHz, $CDCl_3$)

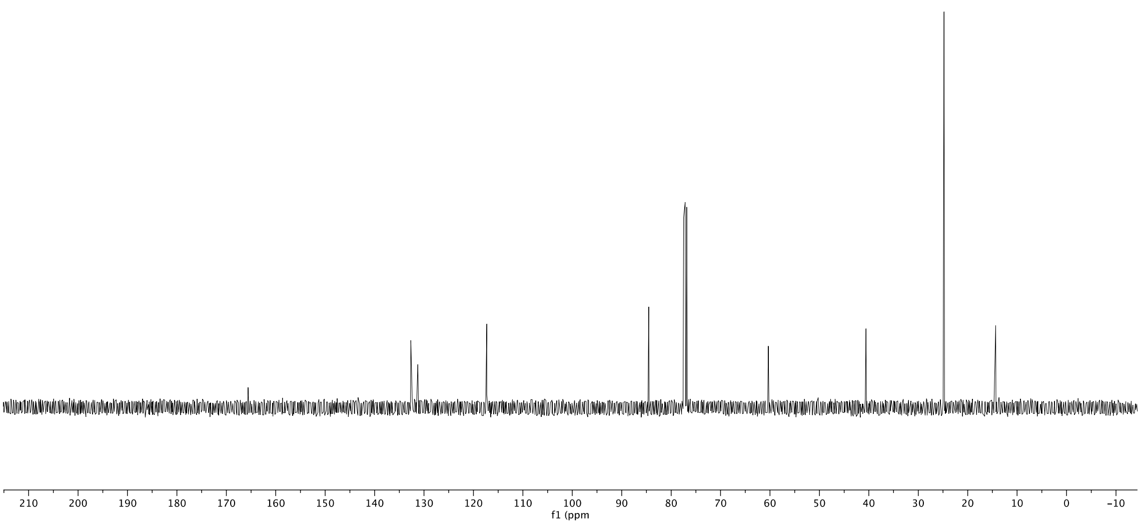

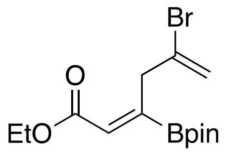

11

^{11}B NMR (129 MHz, CDCl$_3$)

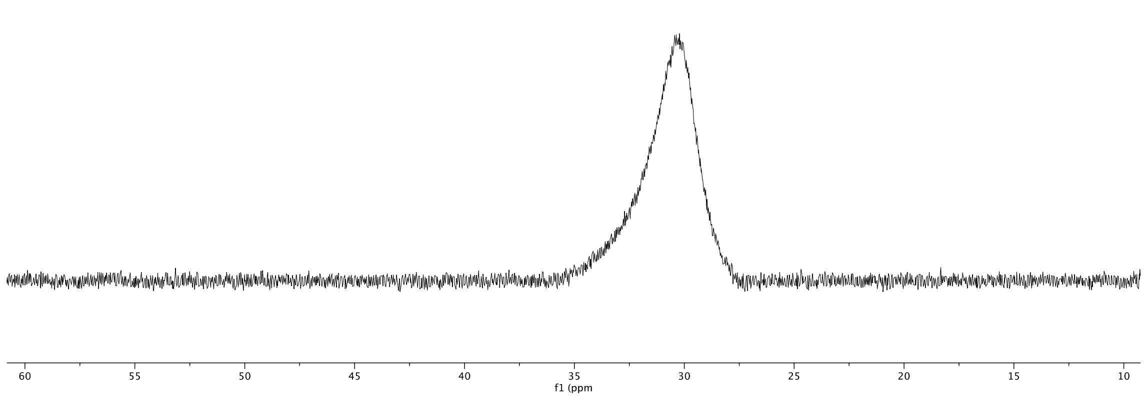

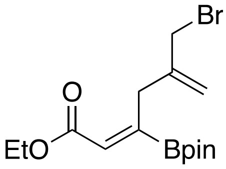

12

^{1}H NMR (400 MHz, CDCl$_3$)

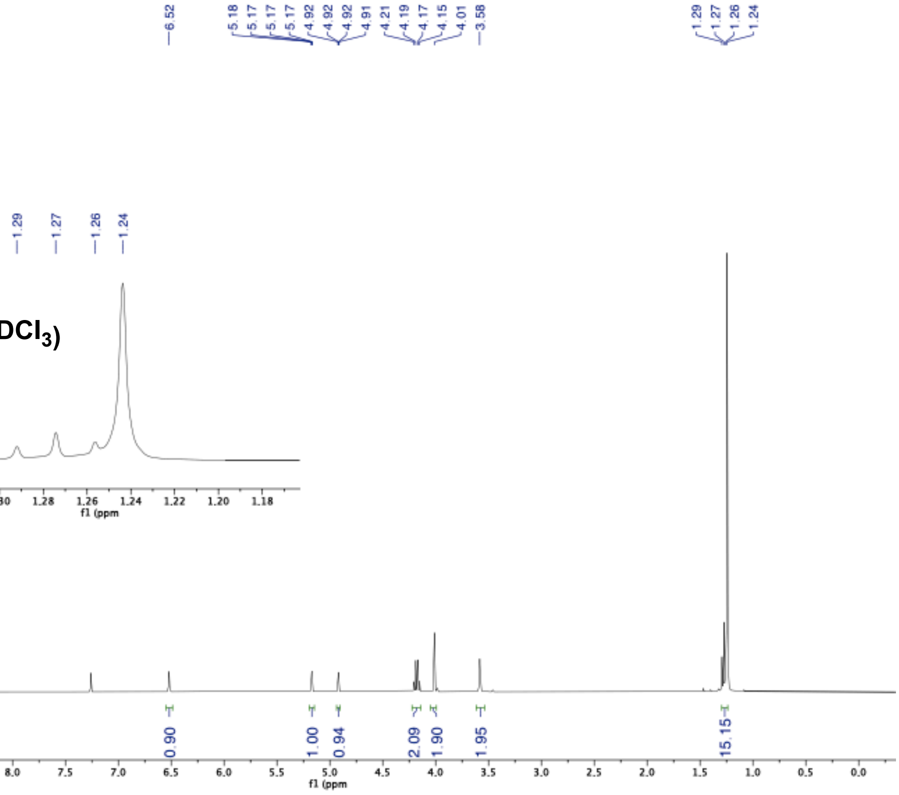

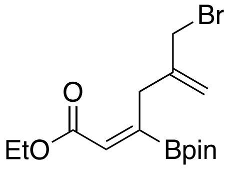

12

^{13}C NMR (100 MHz, CDCl$_3$)

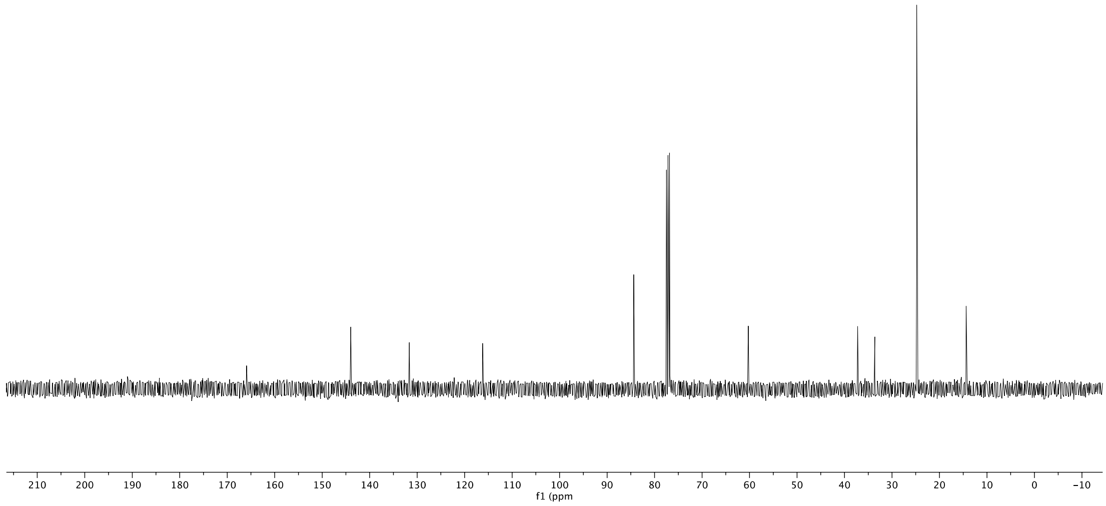

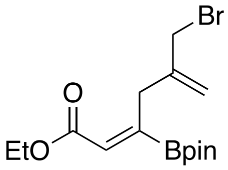

12

^{11}B NMR (129 MHz, CDCl$_3$)

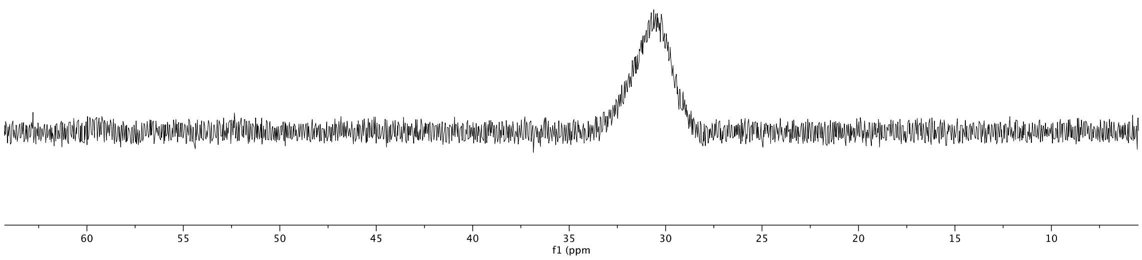

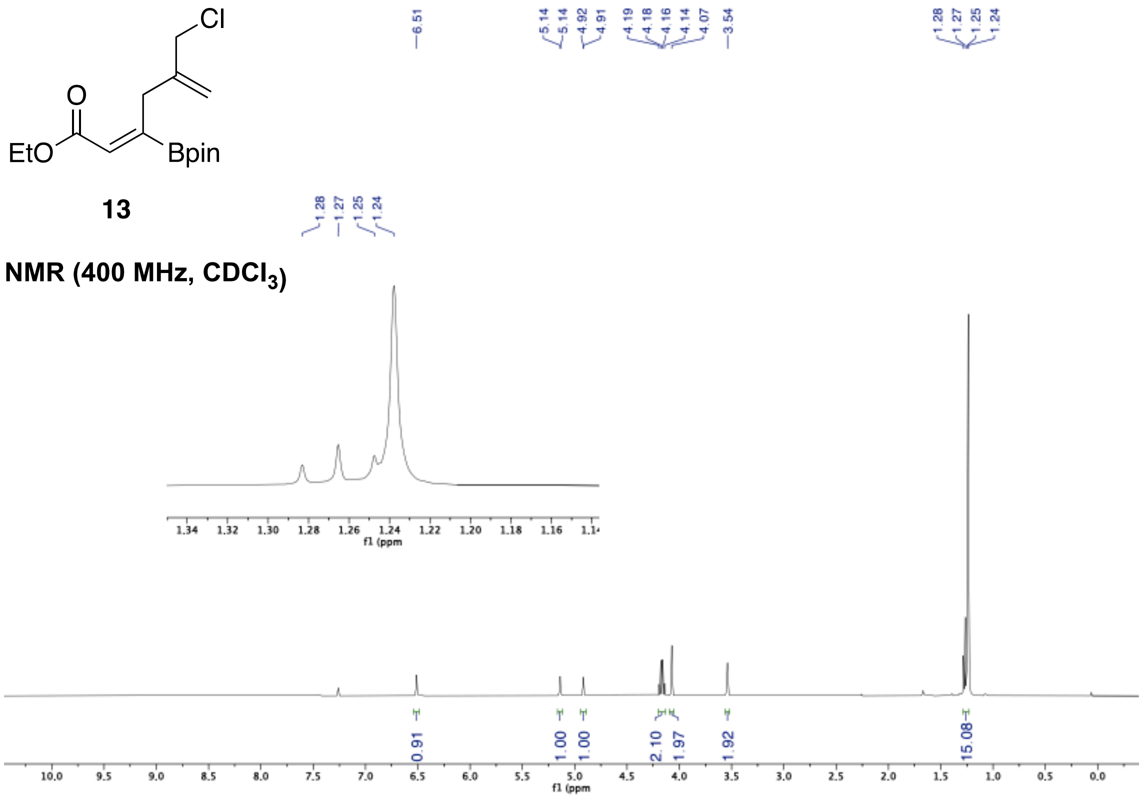

13

^{1}H NMR (400 MHz, $CDCl_3$)

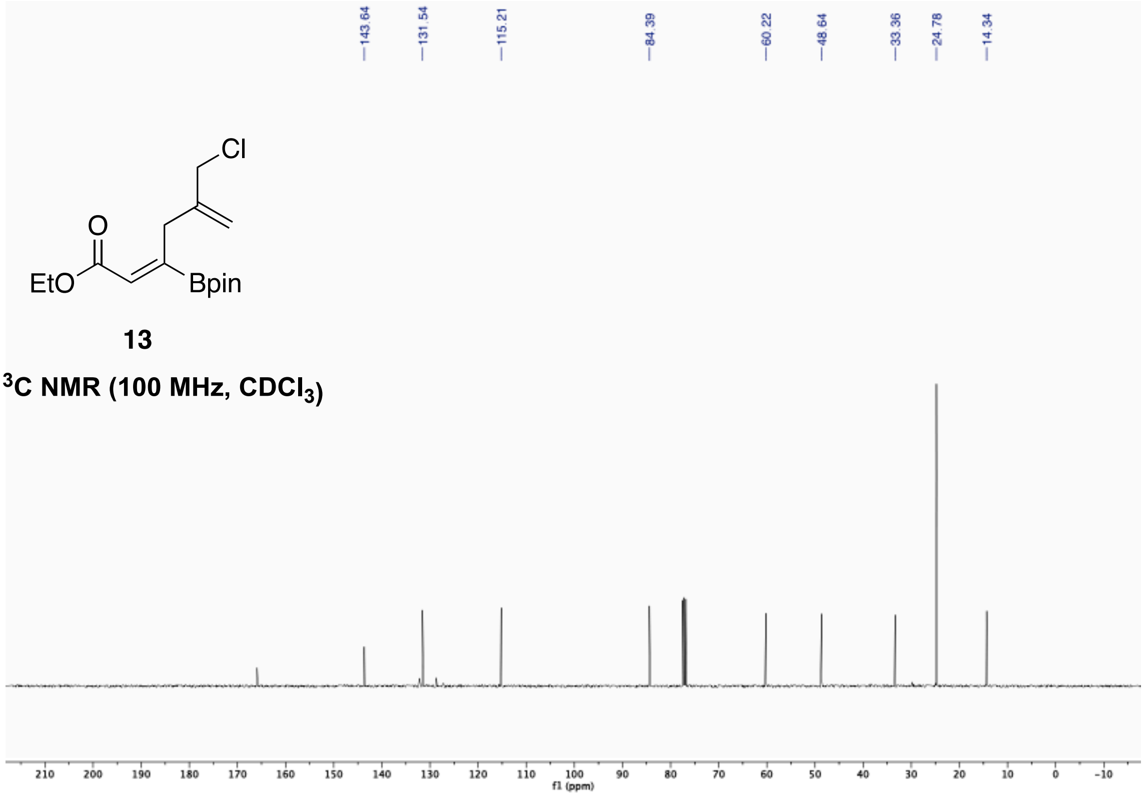

13

^{13}C NMR (100 MHz, $CDCl_3$)

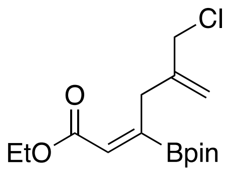

13

^{11}B NMR (129 MHz, $CDCl_3$)

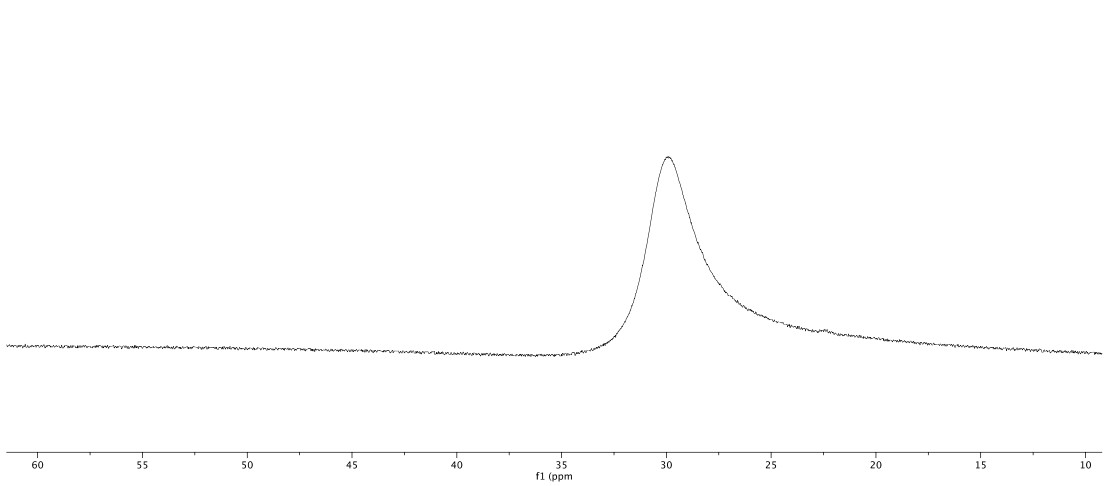

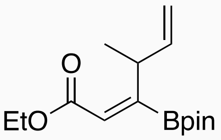

14

^{1}H NMR (400 MHz, $CDCl_3$)

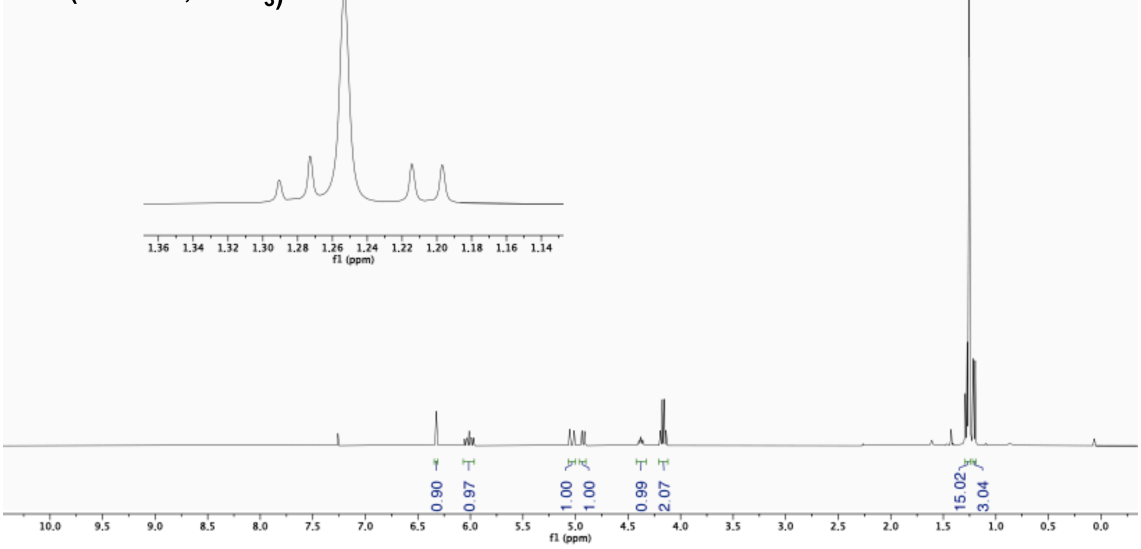

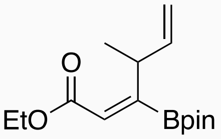

14

^{13}C NMR (100 MHz, $CDCl_3$)

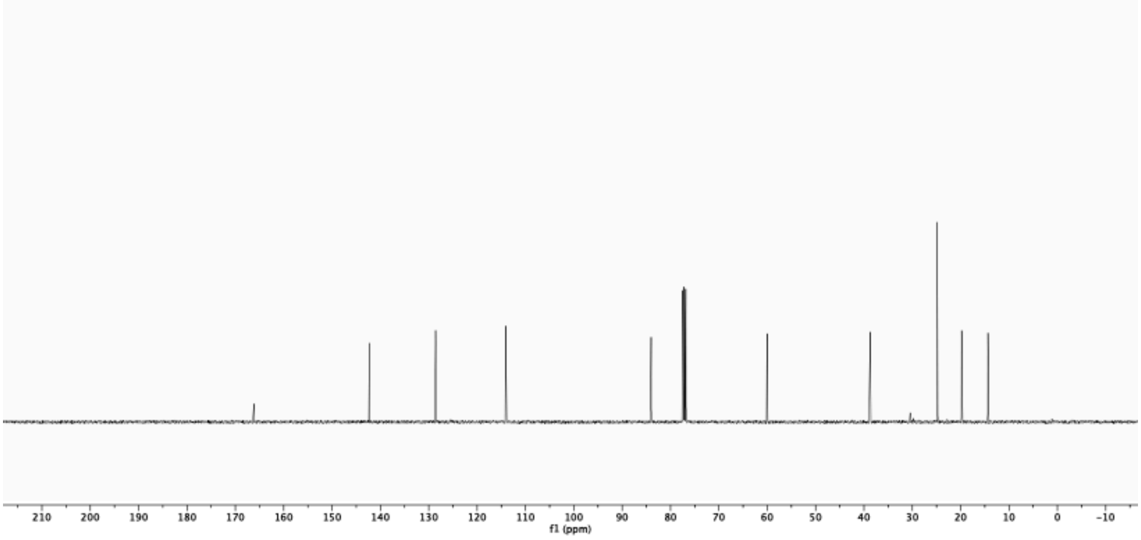

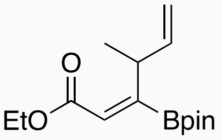

14

^{11}B NMR (129 MHz, $CDCl_3$)

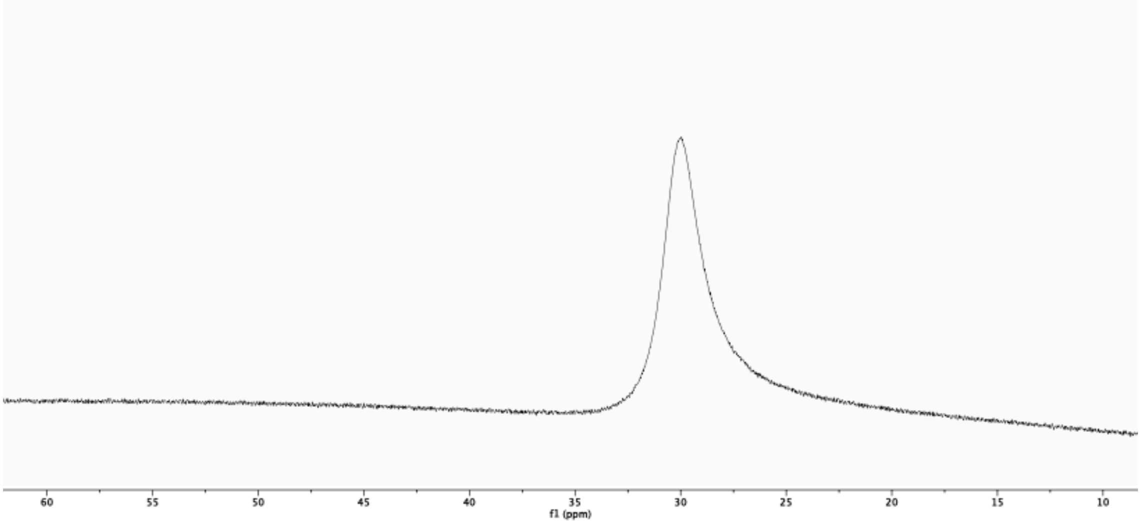

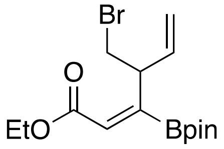

15

^{1}H NMR (400 MHz, CDCl$_3$)

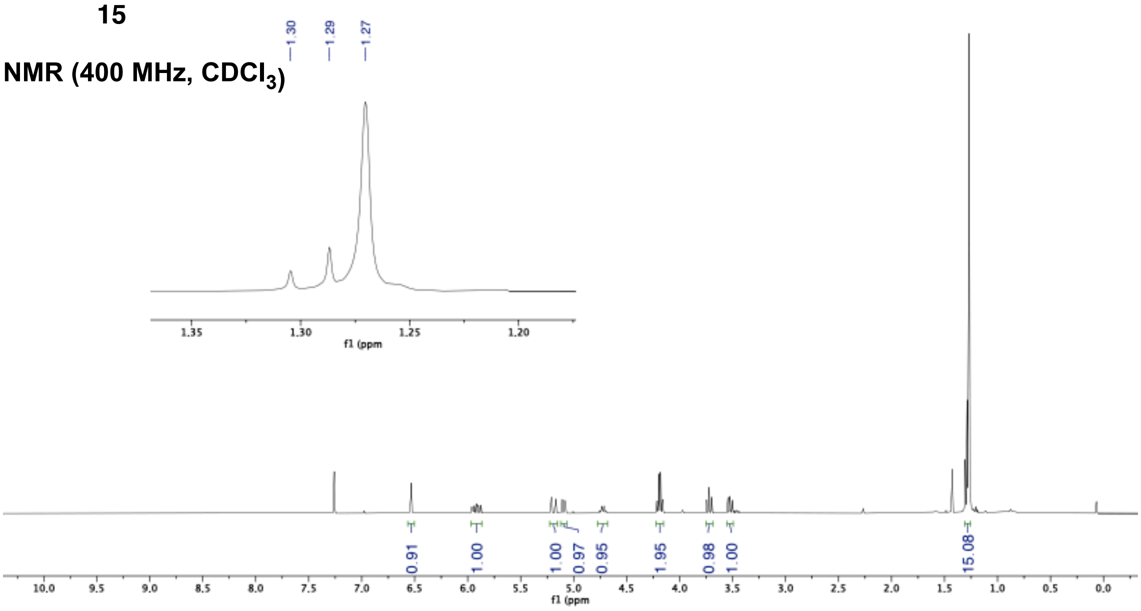

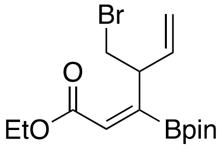

15

^{13}C NMR (100 MHz, CDCl$_3$)

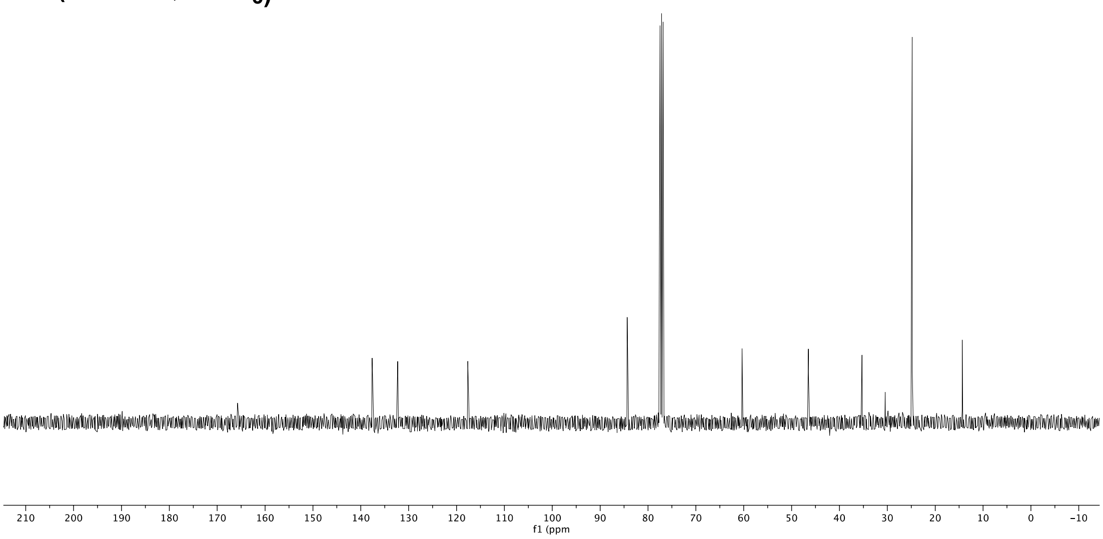

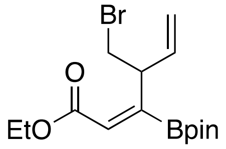

15

^{11}B NMR (129 MHz, $CDCl_3$)

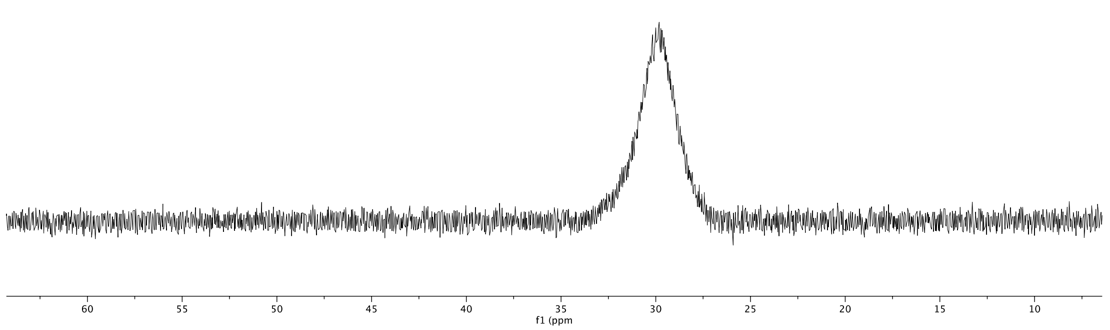

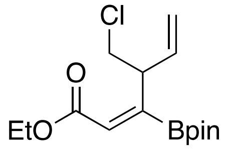

16

^{1}H NMR (400 MHz, $CDCl_3$)

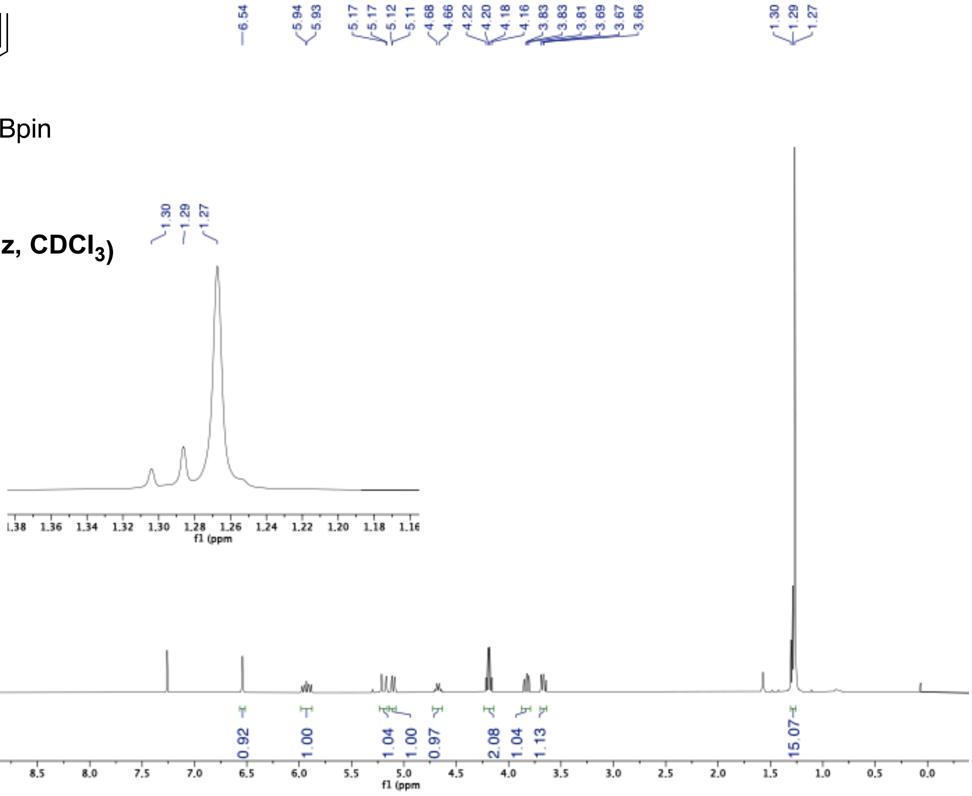

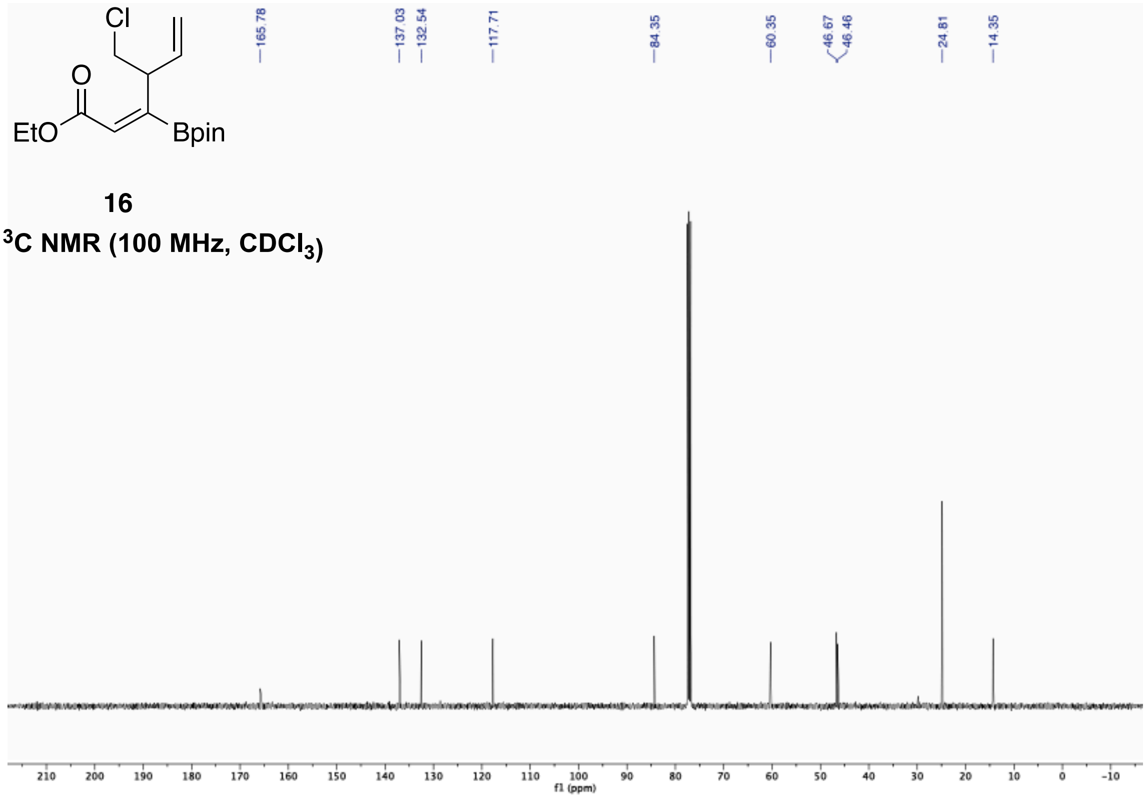

16

^{13}C NMR (100 MHz, $CDCl_3$)

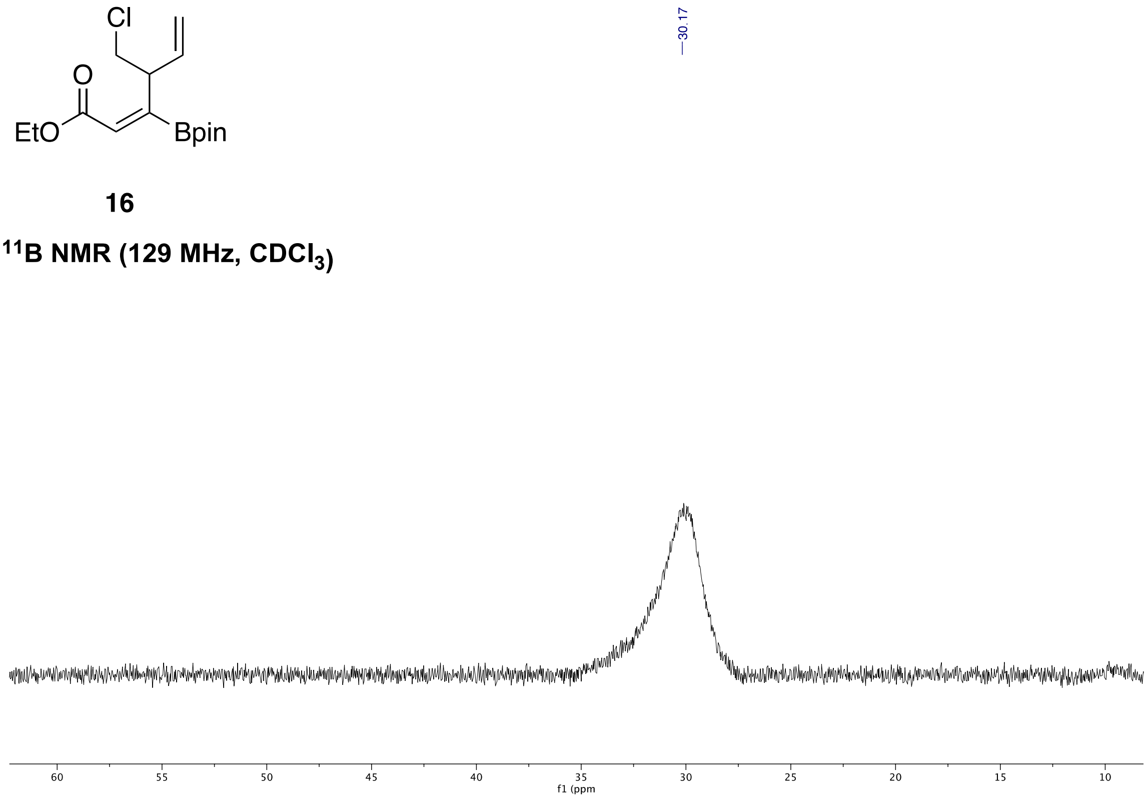

16

^{11}B NMR (129 MHz, $CDCl_3$)

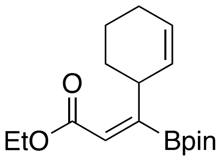

17a

^{1}H NMR (400 MHz, $CDCl_3$)

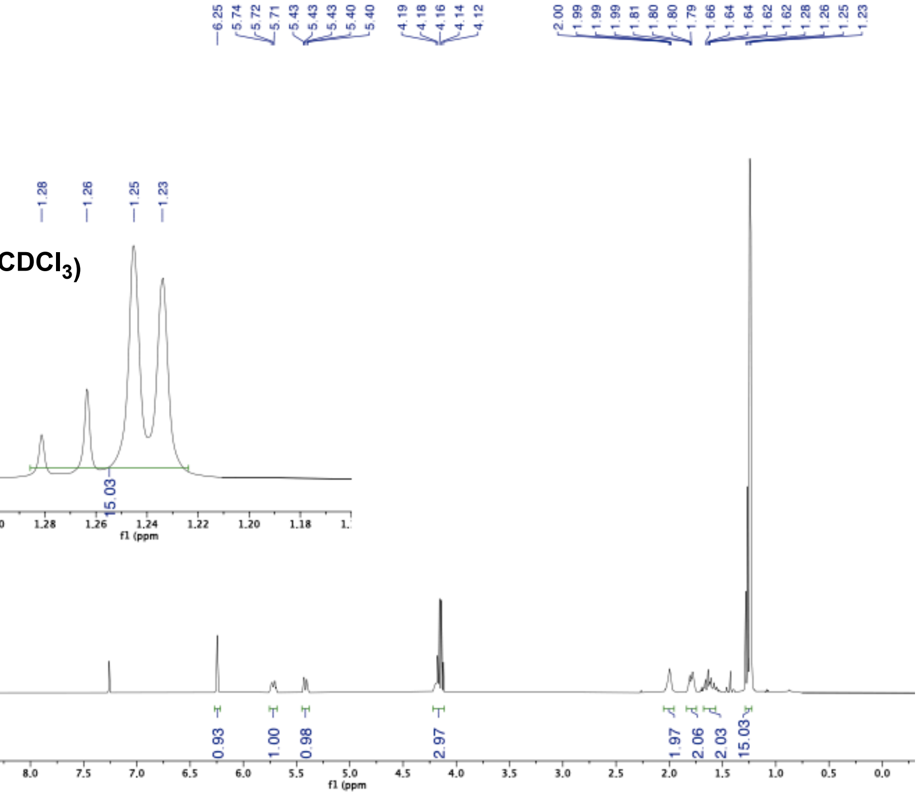

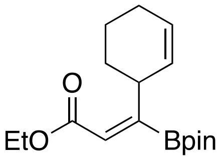

17a

^{13}C NMR (100 MHz, $CDCl_3$)

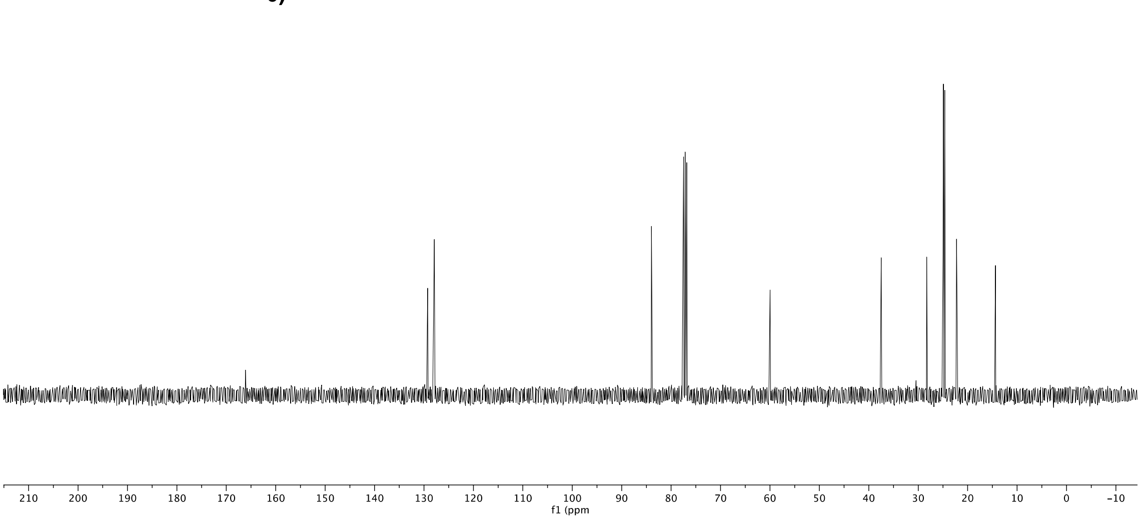

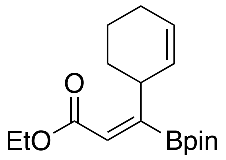

17a

^{11}B NMR (129 MHz, $CDCl_3$)

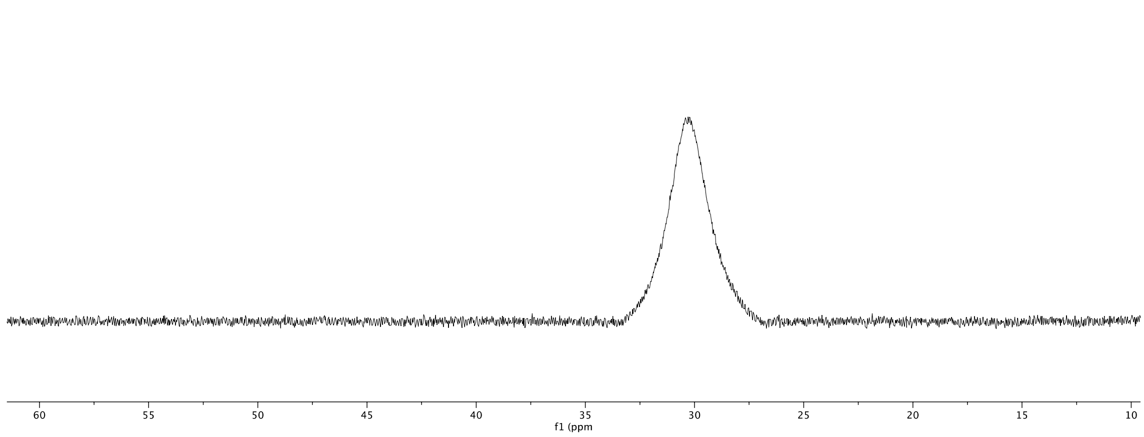

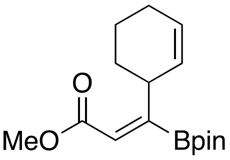

17b

^{1}H NMR (400 MHz, $CDCl_3$)

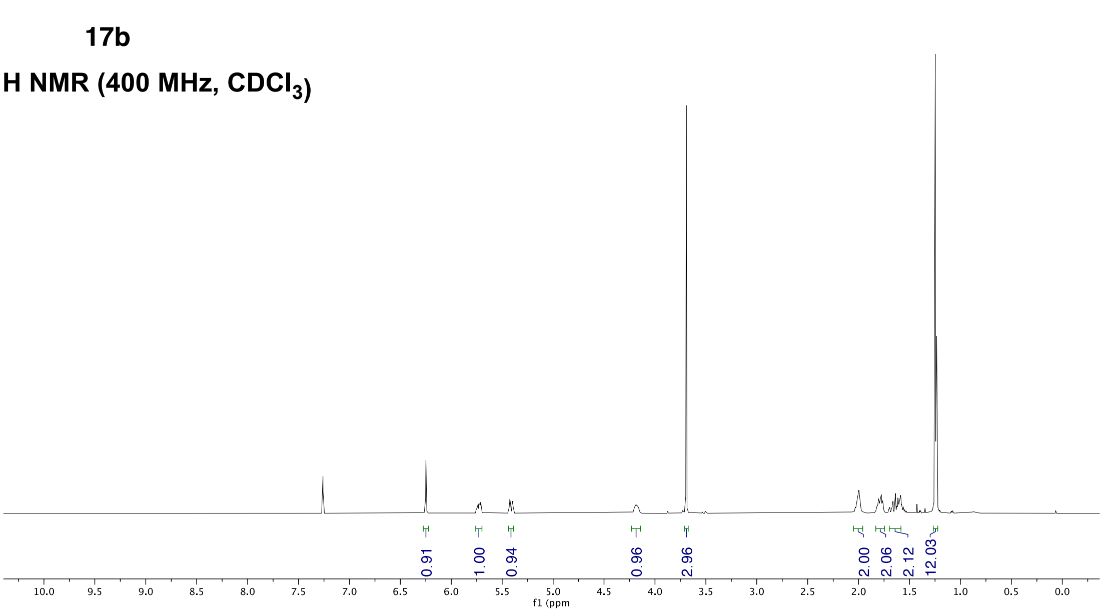

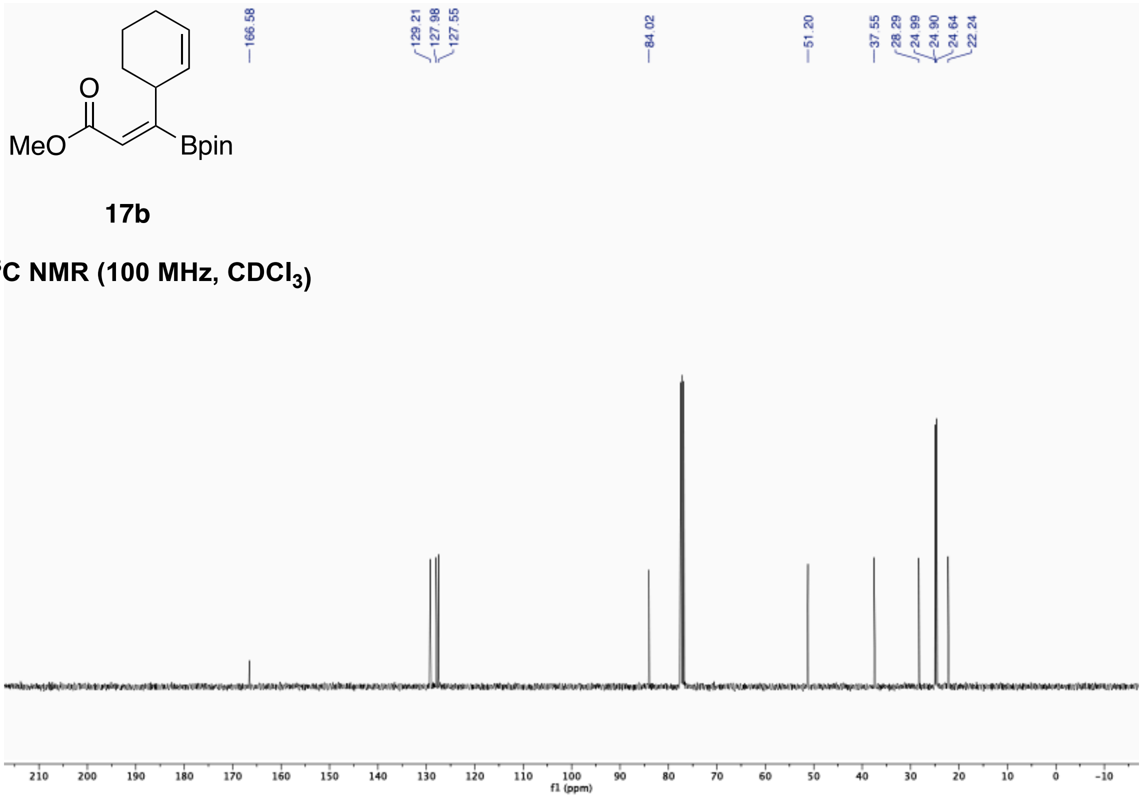

17b

^{13}C NMR (100 MHz, $CDCl_3$)

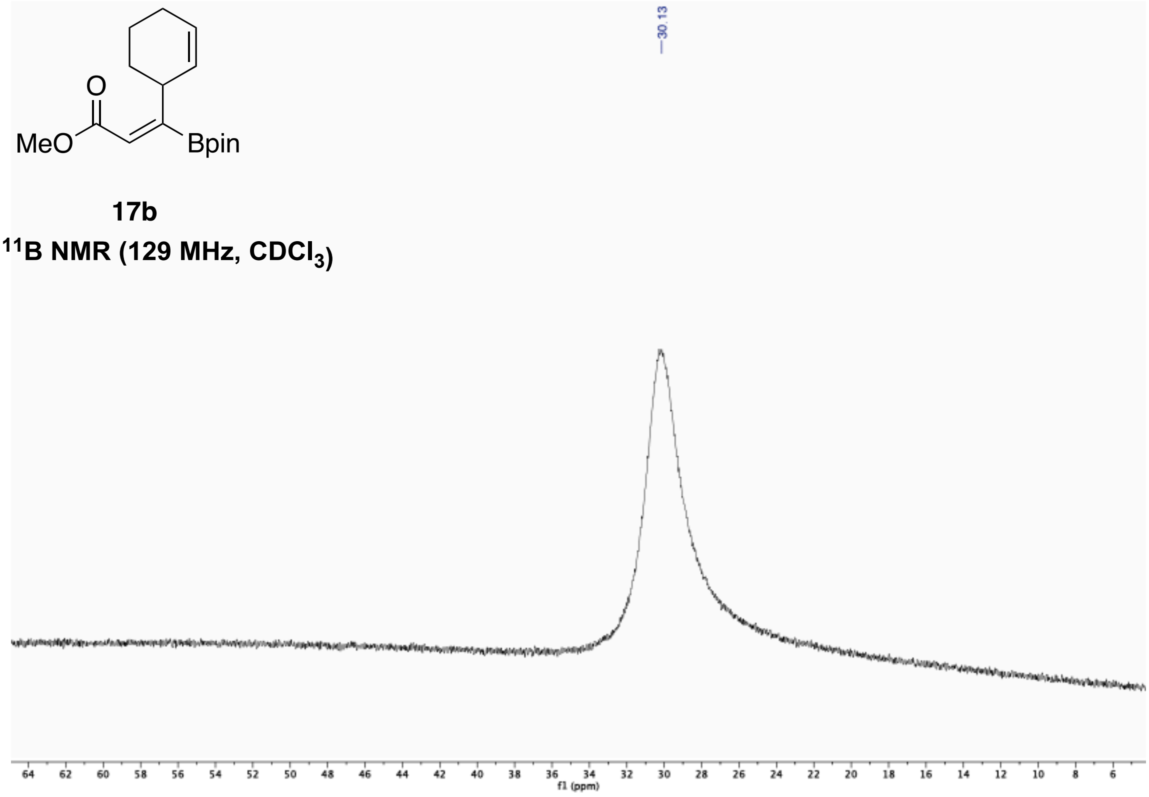

17b

^{11}B NMR (129 MHz, $CDCl_3$)

^{1}H, ^{13}C, ^{11}B Spectra for lactones

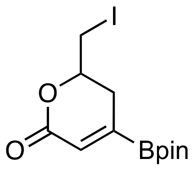

18

^{1}H NMR (400 MHz, $CDCl_3$)

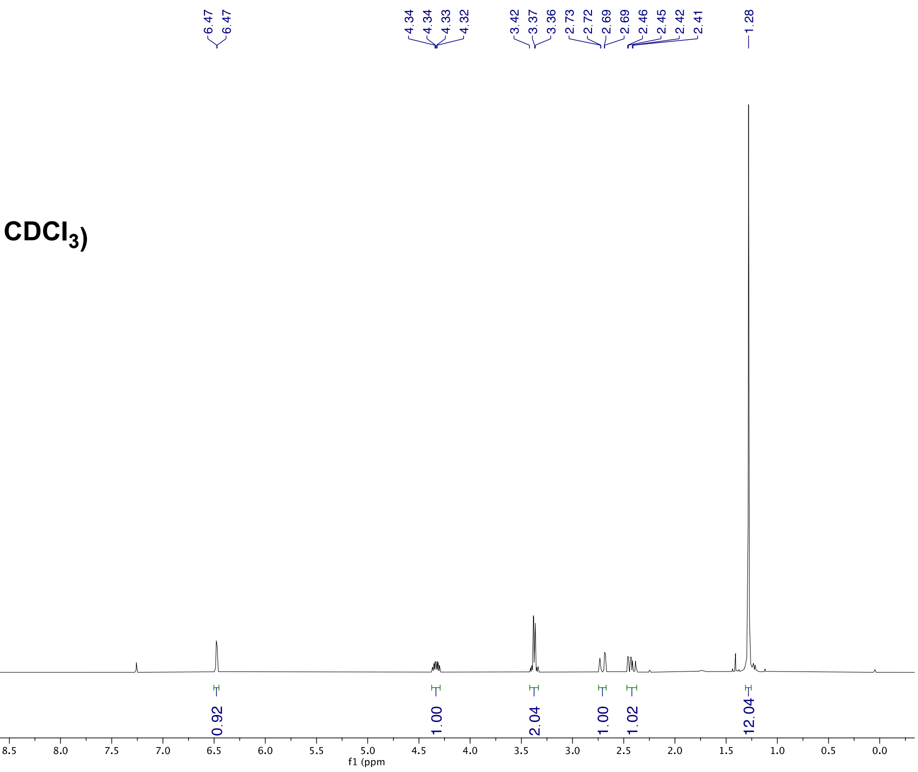

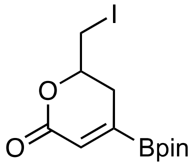

18

^{13}C NMR (100 MHz, $CDCl_3$)

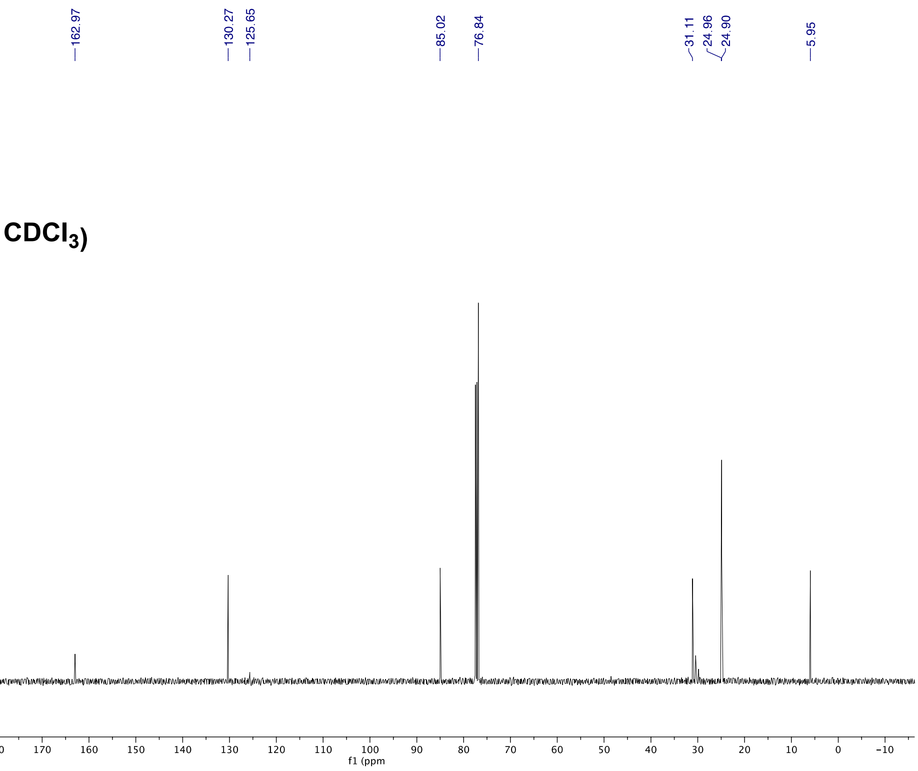

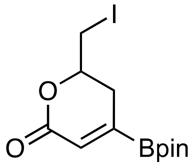

18

^{11}B NMR (129 MHz, $CDCl_3$)

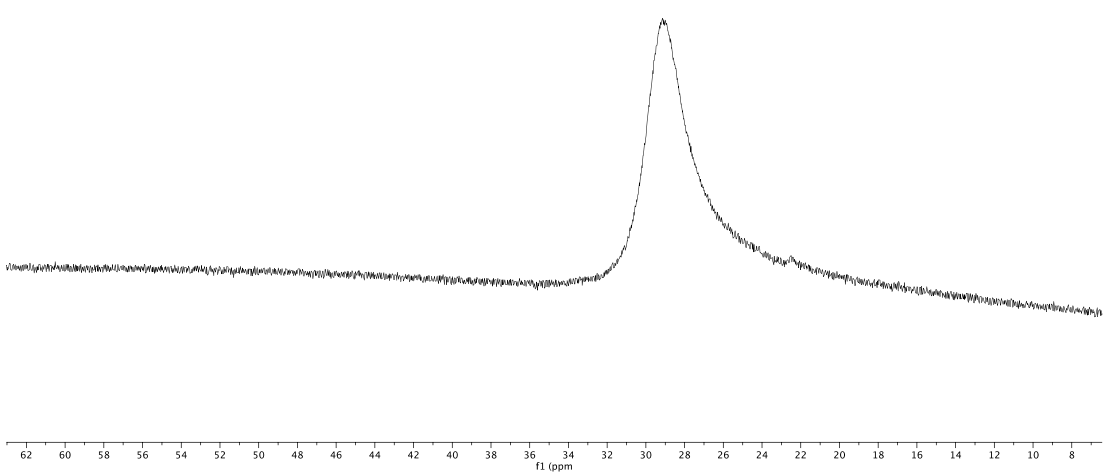

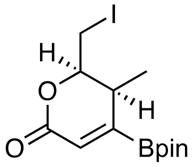

19

^{1}H NMR (400 MHz, $CDCl_3$)

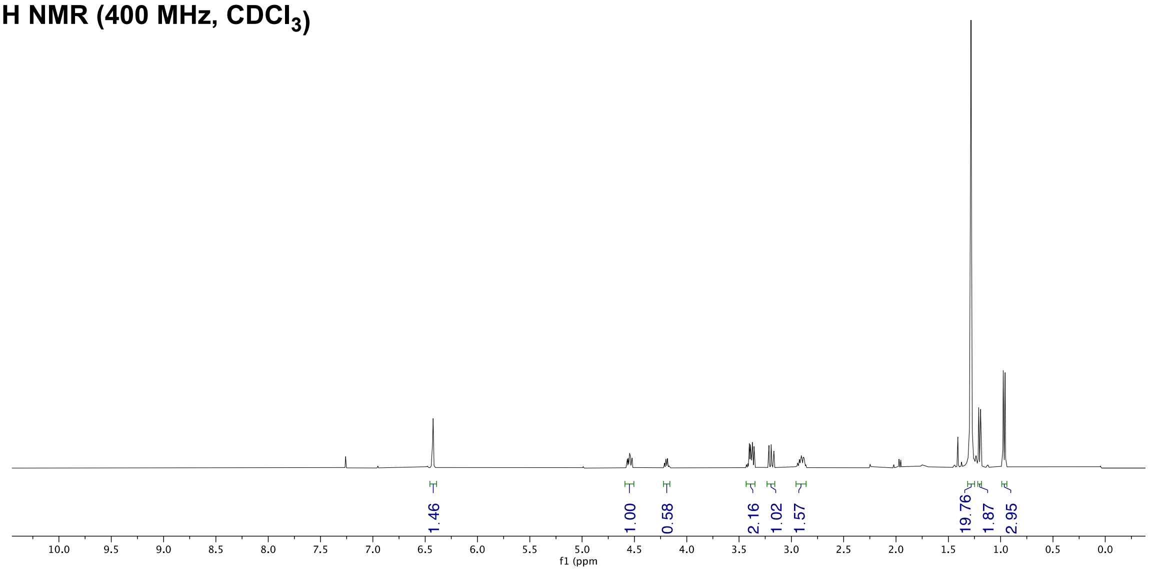

1-D NMR NOE EXPERIMENT

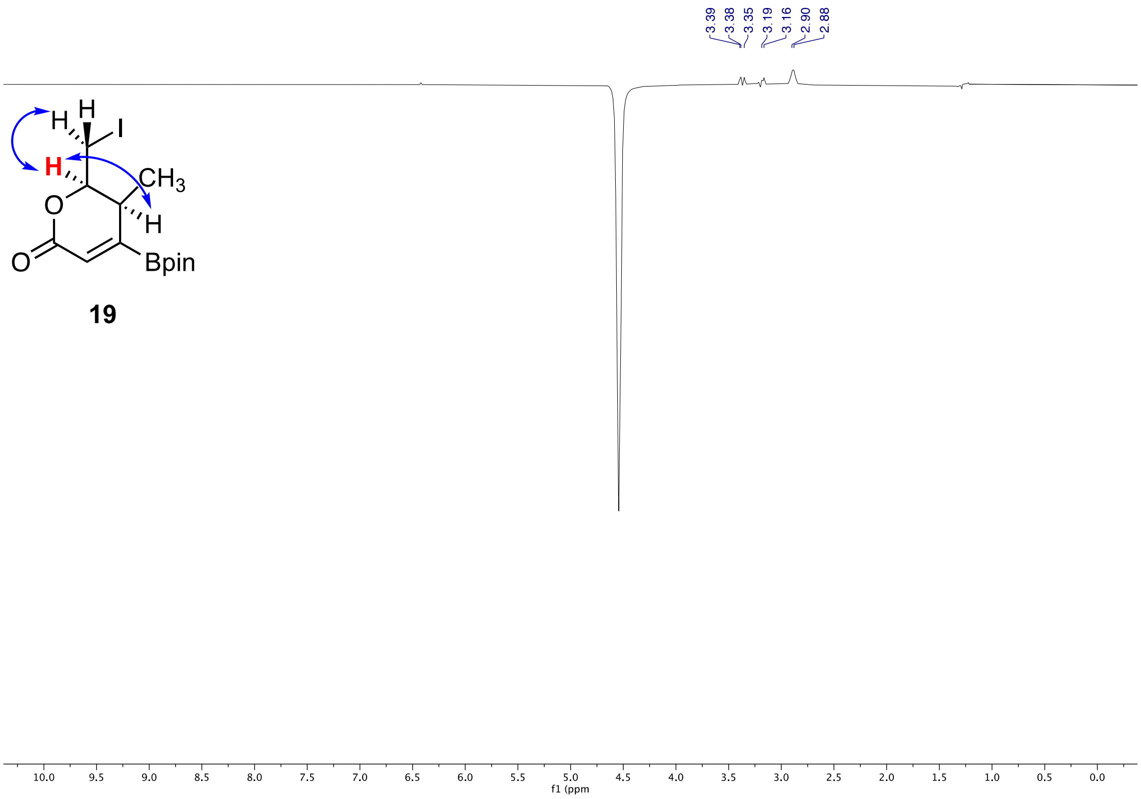

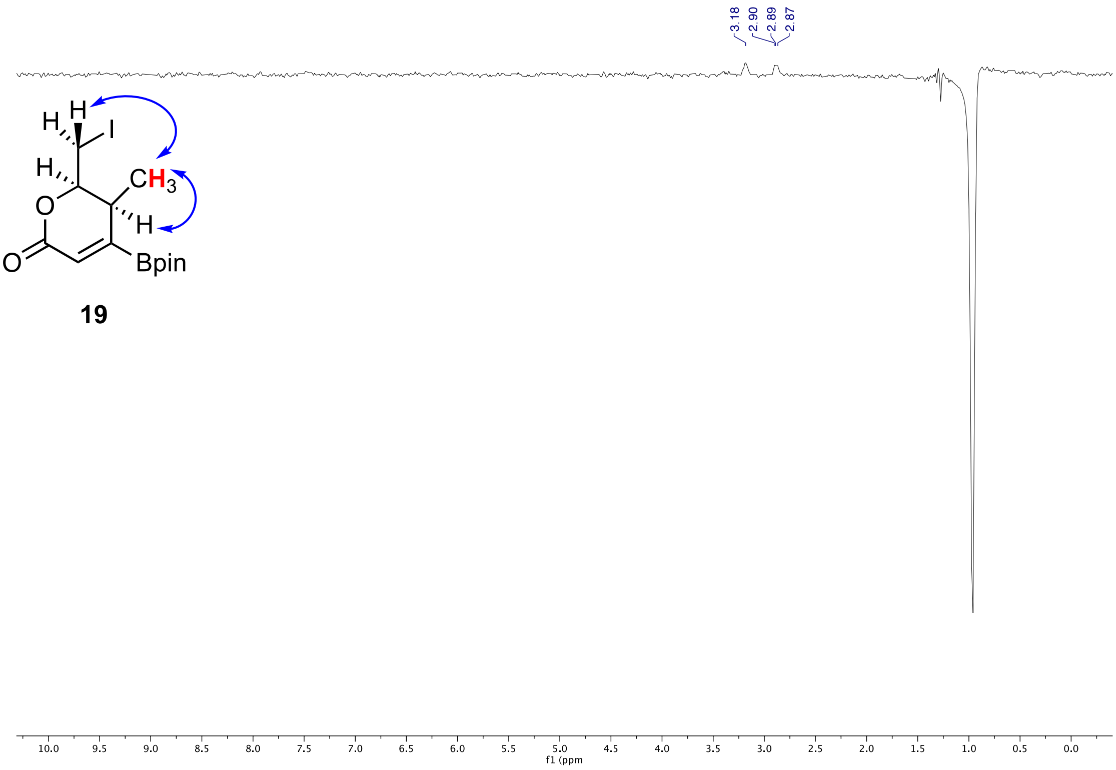

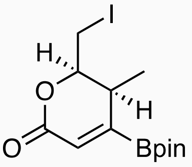

19

^{13}C NMR (100 MHz, $CDCl_3$)

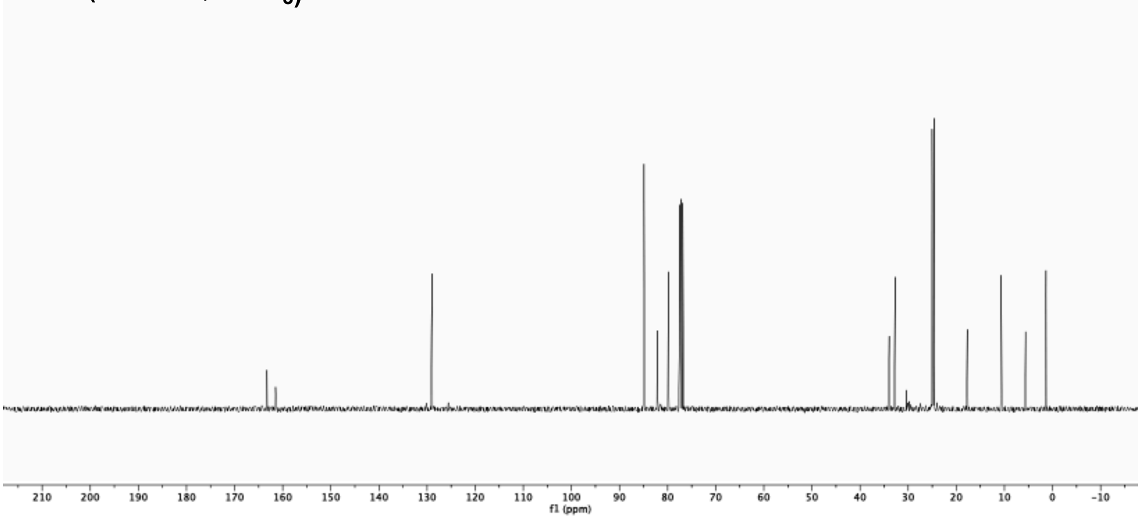

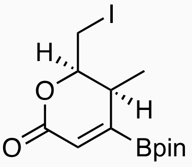

19

^{11}B NMR (129 MHz, $CDCl_3$)

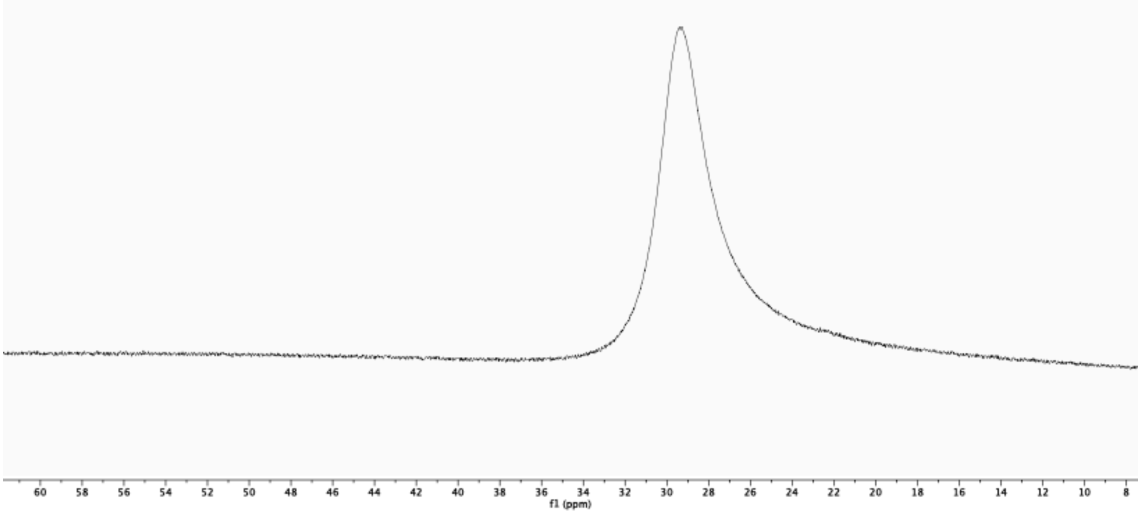

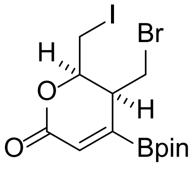

20

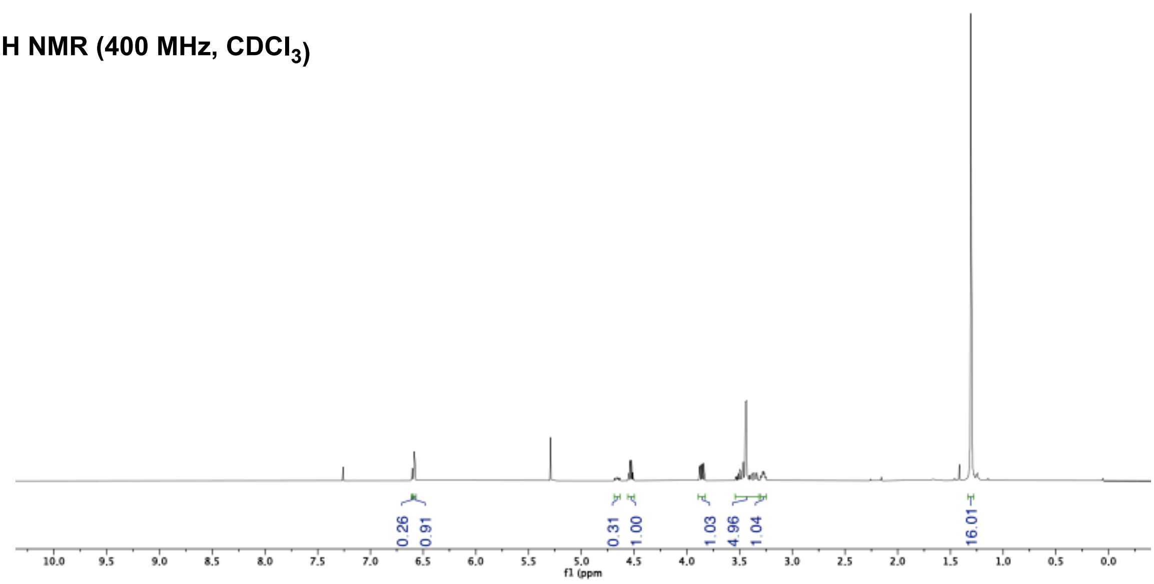

^{1}**H NMR (400 MHz, CDCl$_3$)**

1-D NMR NOE EXPERIMENT

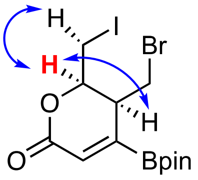

20

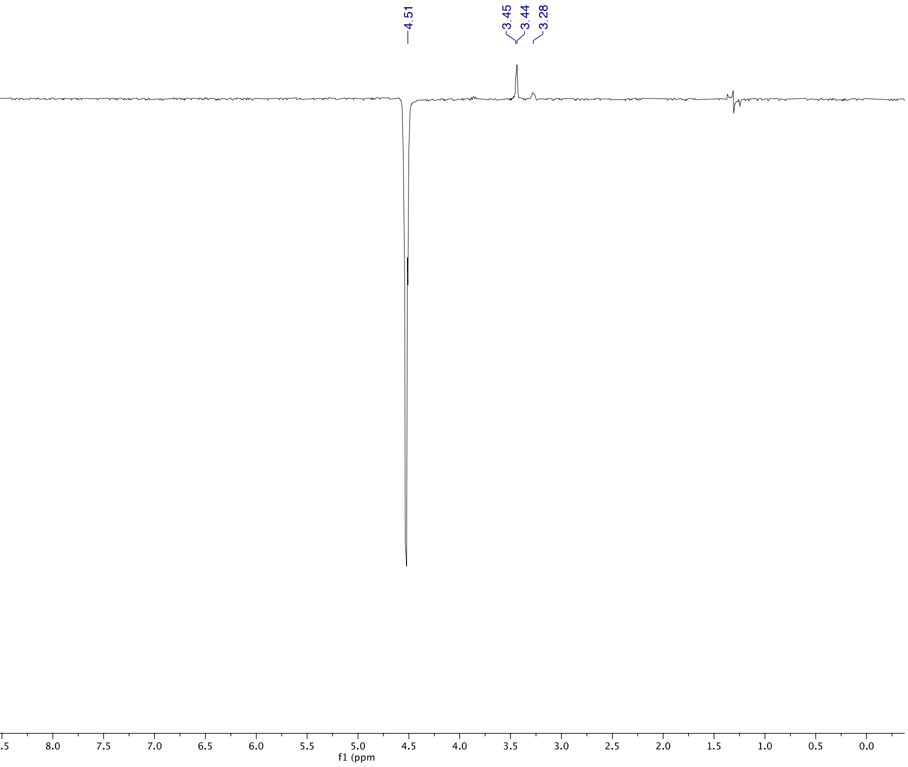

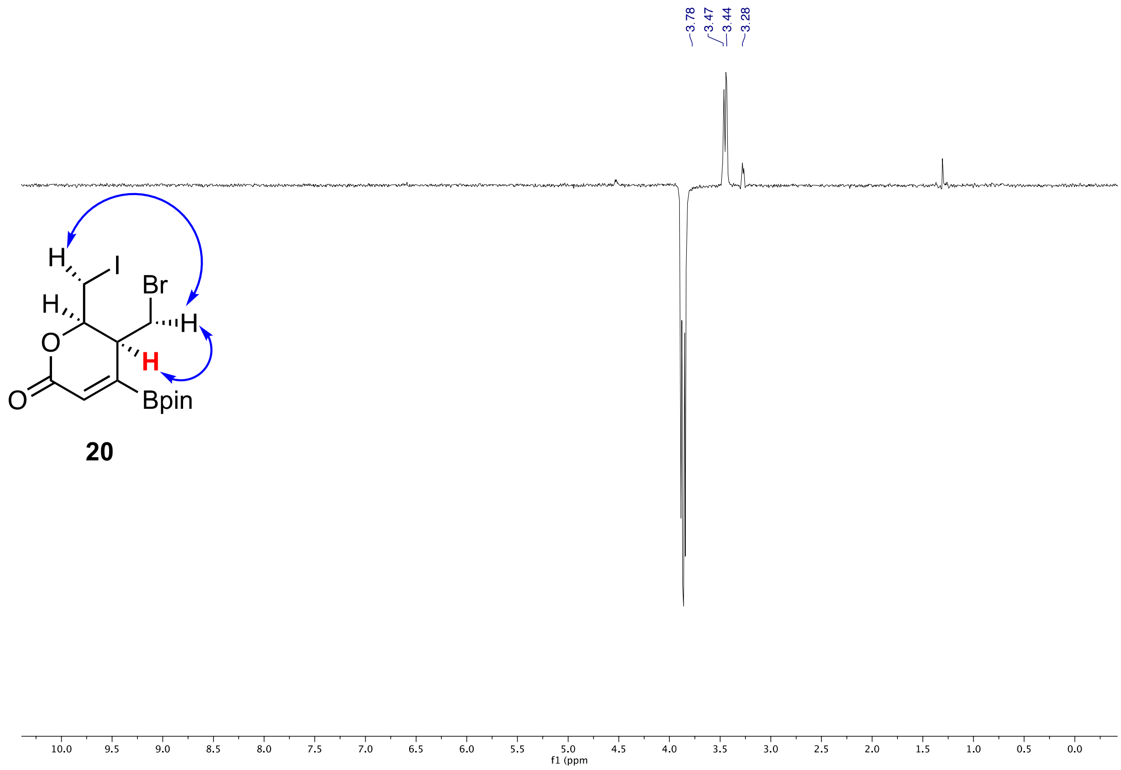
3.78
3.47
3.44
3.28
H
I
H
Br
O
H
H
O
Bpin
20
10.0 9.5 9.0 8.5 8.0 7.5 7.0 6.5 6.0 5.5 5.0 4.5 4.0 3.5 3.0 2.5 2.0 1.5 1.0 0.5 0.0
f1 (ppm

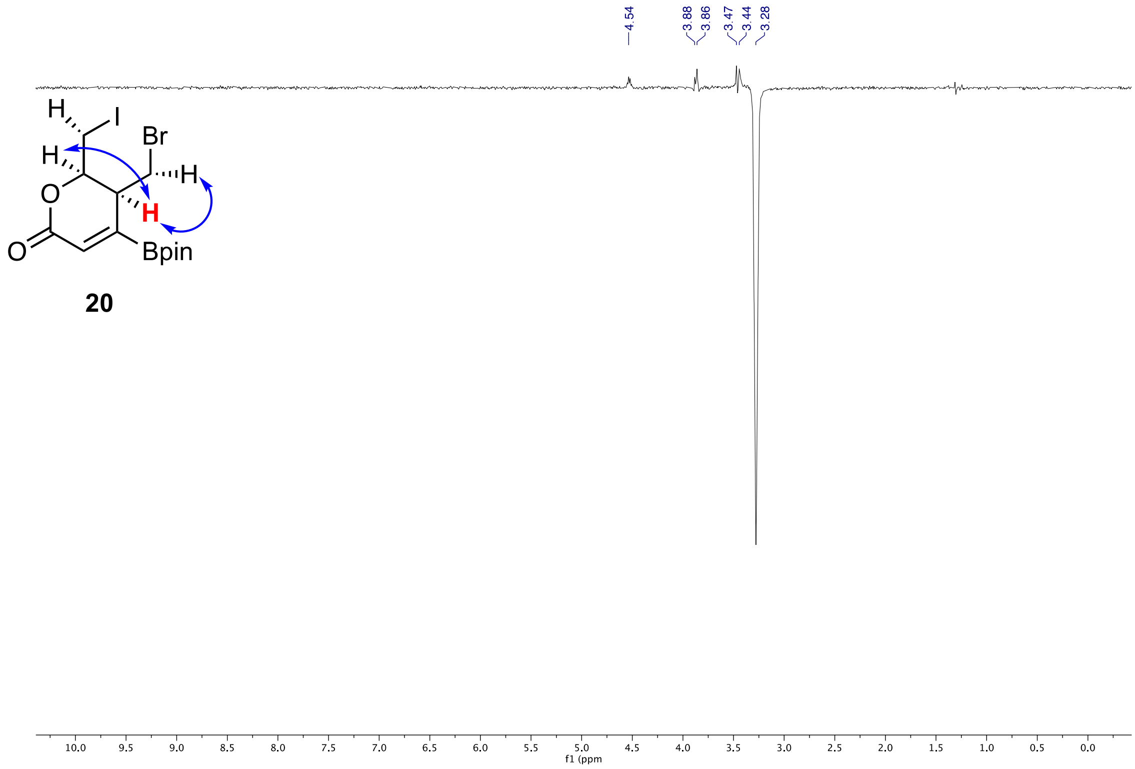
4.54
3.88
3.86
3.47
3.44
3.28
H
I
H
Br
H
O
H
O
Bpin
20
10.0 9.5 9.0 8.5 8.0 7.5 7.0 6.5 6.0 5.5 5.0 4.5 4.0 3.5 3.0 2.5 2.0 1.5 1.0 0.5 0.0
f1 (ppm

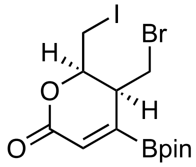

20

^{13}C NMR (100 MHz, $CDCl_3$)

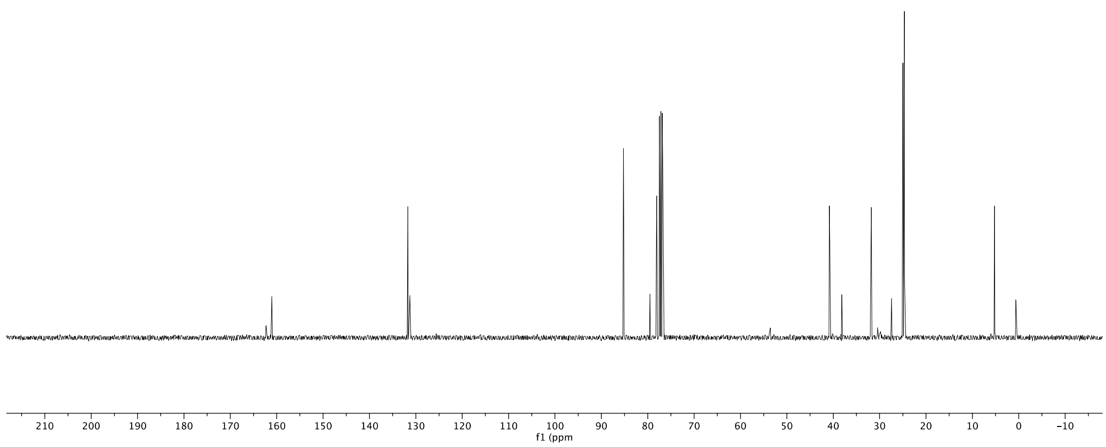

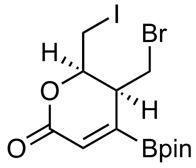

20

^{11}B NMR (129 MHz, $CDCl_3$)

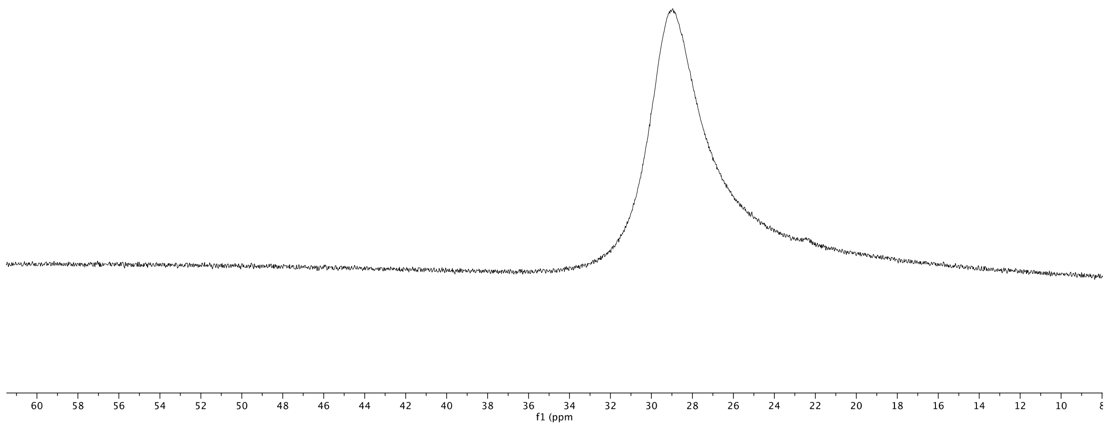

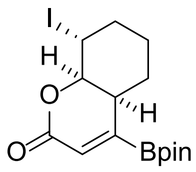

21

^{1}H NMR (400 MHz, $CDCl_3$)

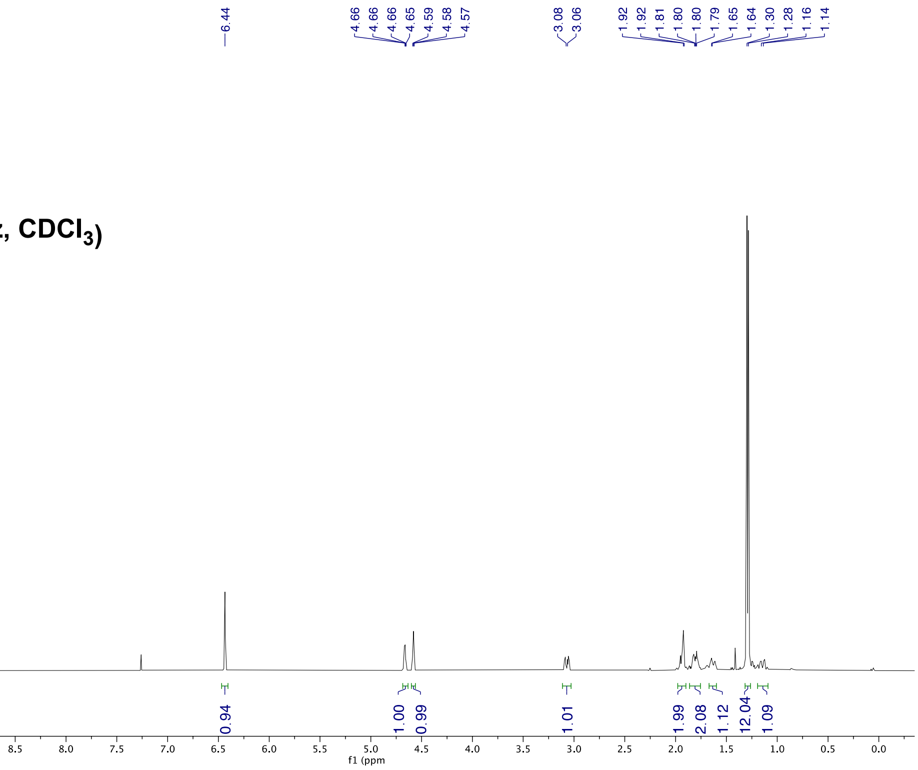

1-D NMR NOE EXPERIMENT

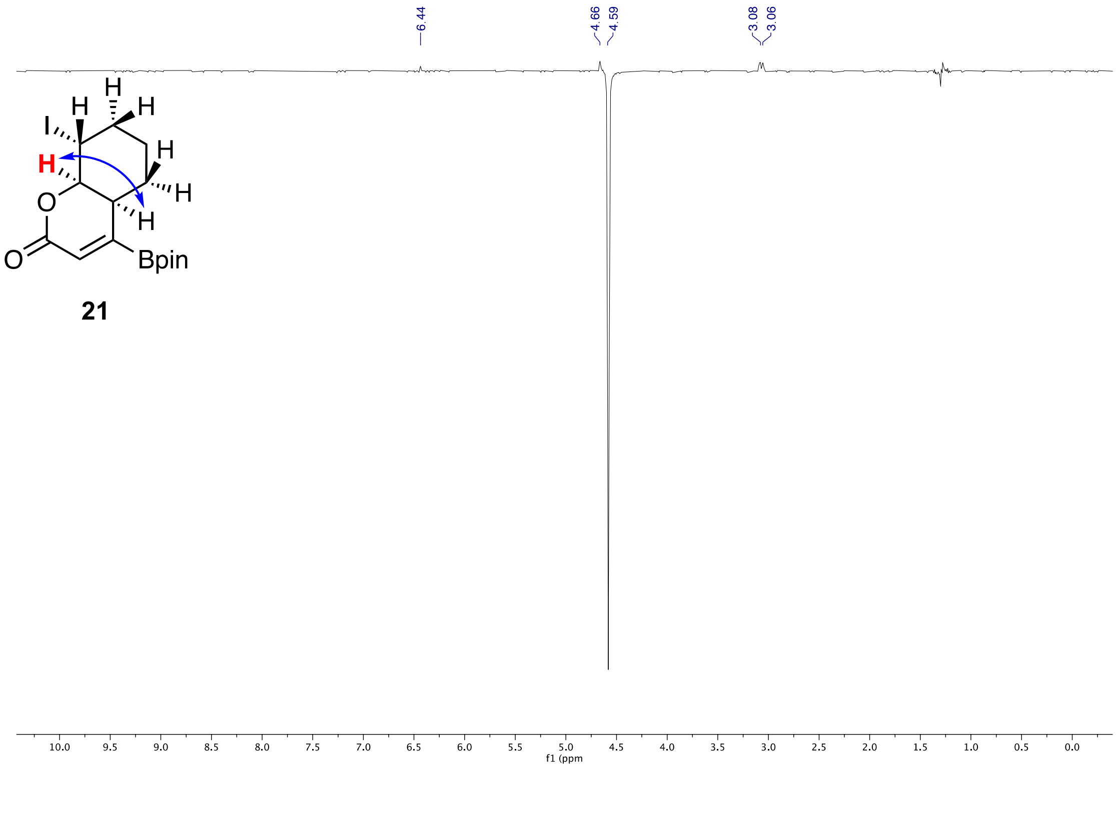

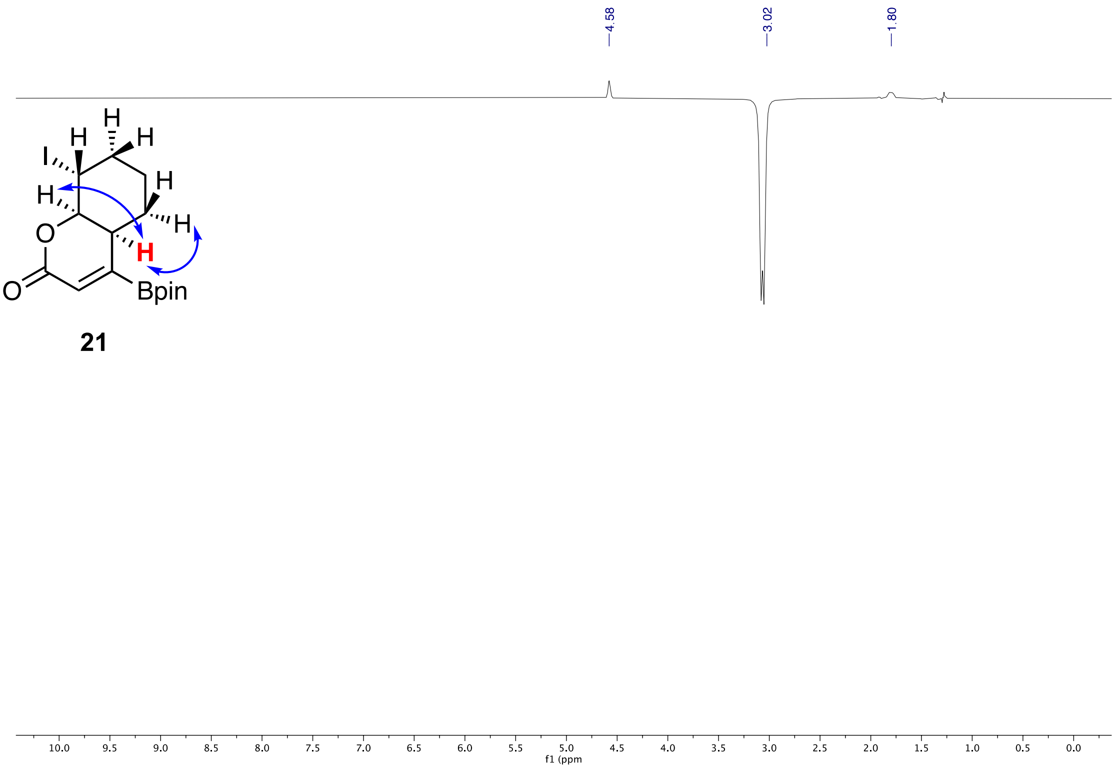
4.58
3.02
1.80
21
f1 (ppm)

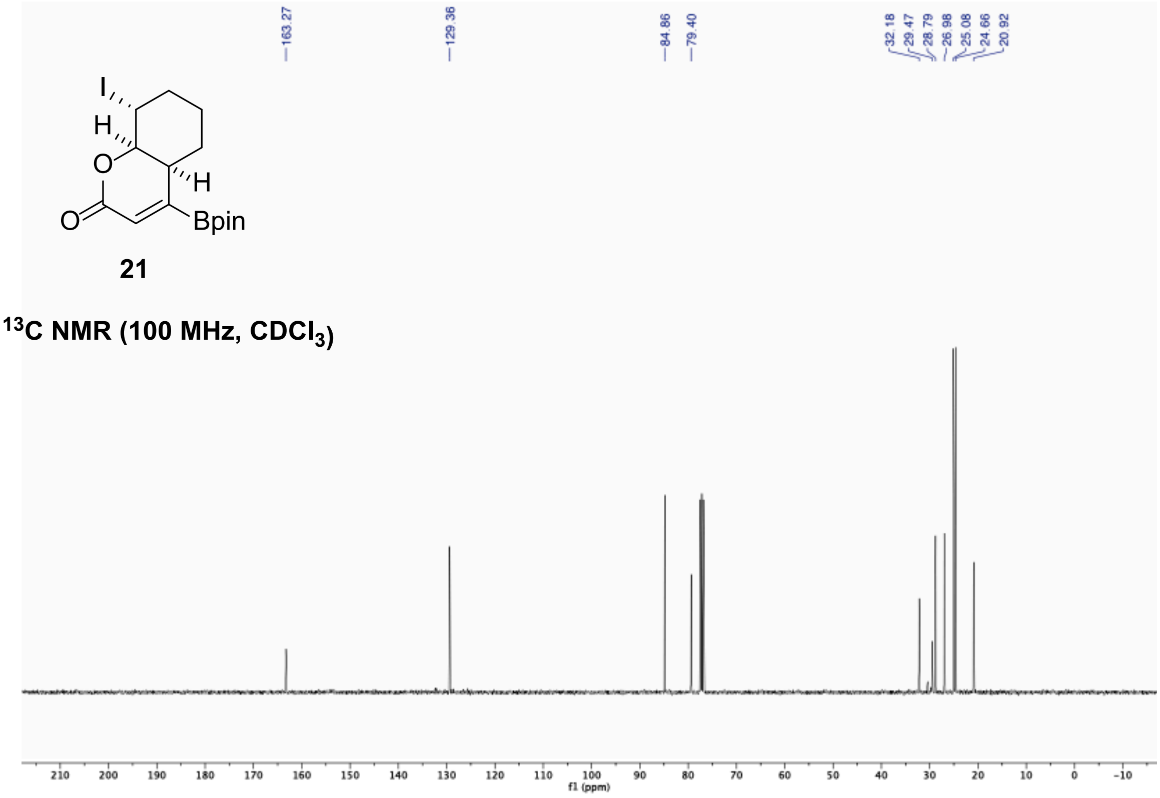
163.27
129.36
84.86
79.40
32.18
29.47
28.79
26.98
25.08
24.66
20.92
21
^{13}C NMR (100 MHz, $CDCl_3$)
f1 (ppm)

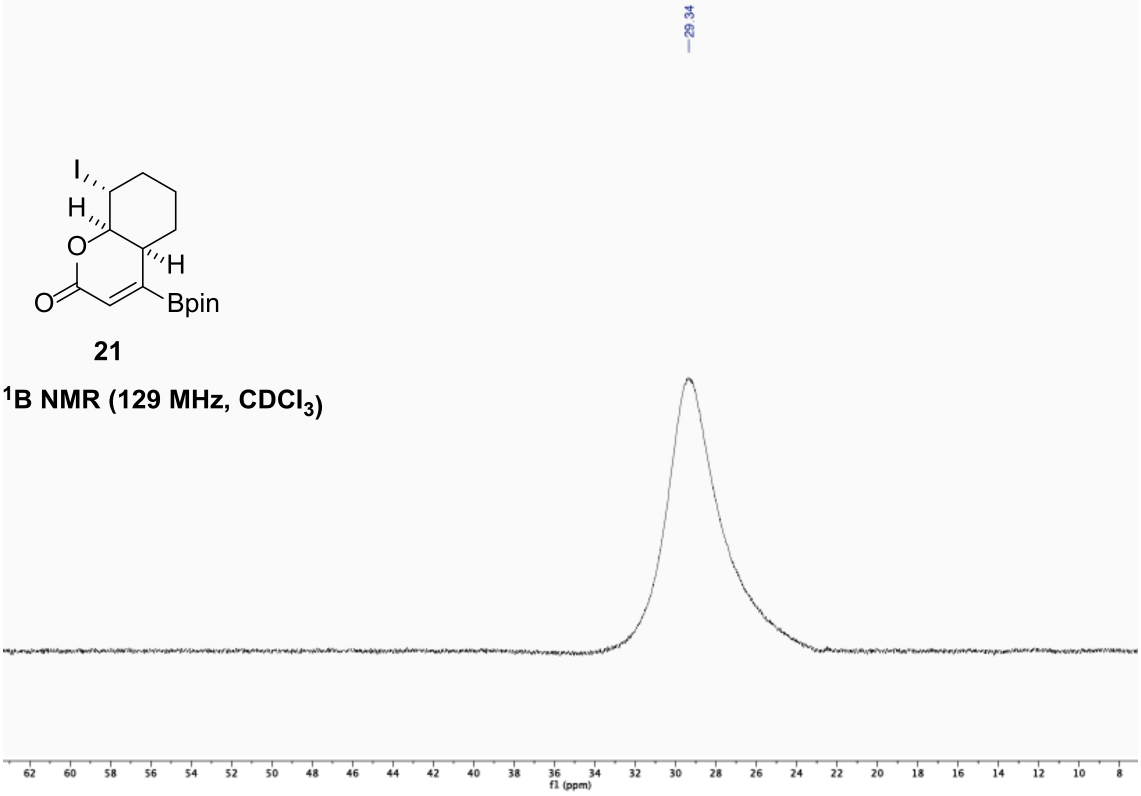

21

^{11}B NMR (129 MHz, CDCl$_3$)

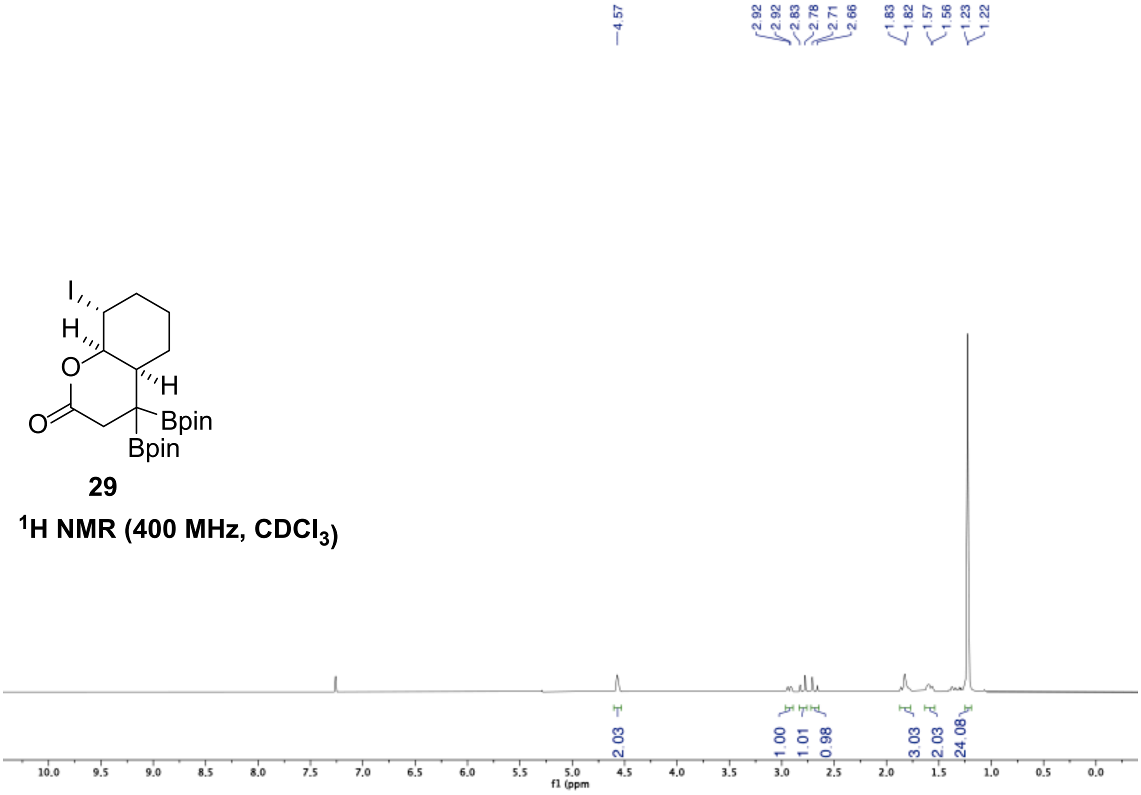

29

^{1}H NMR (400 MHz, CDCl$_3$)

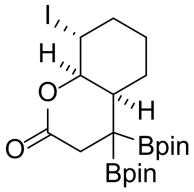

29

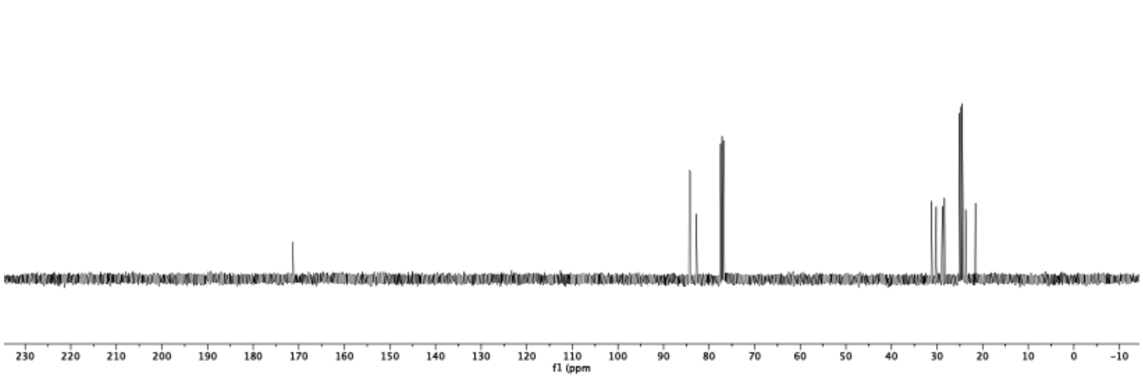

^{13}C NMR (100 MHz, $CDCl_3$)

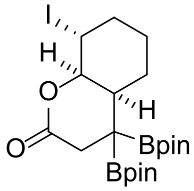

29

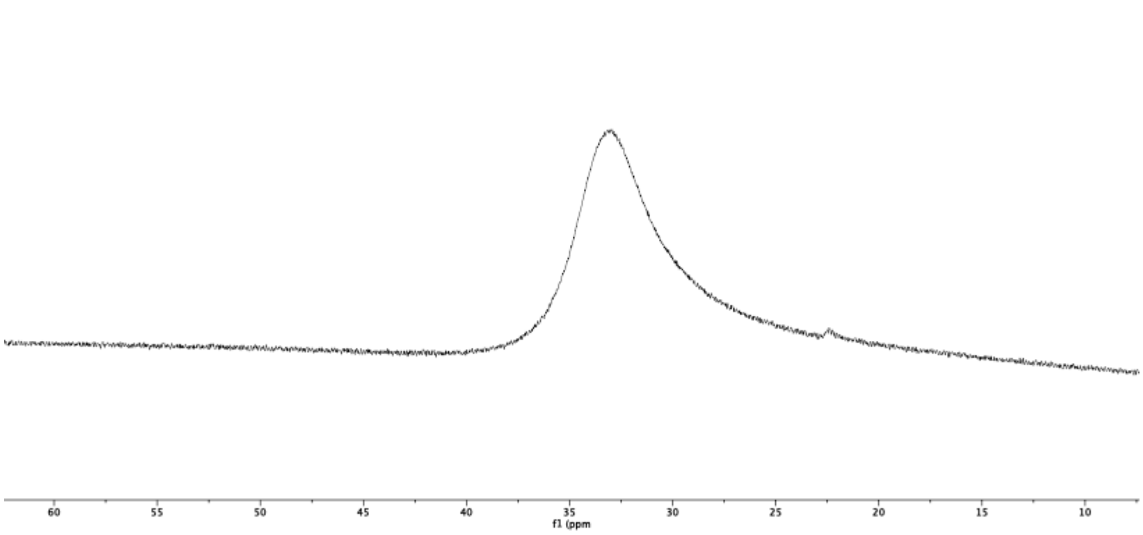

^{11}B NMR (129 MHz, $CDCl_3$)

^{1}H, ^{13}C, ^{11}B Spectra for coupled products

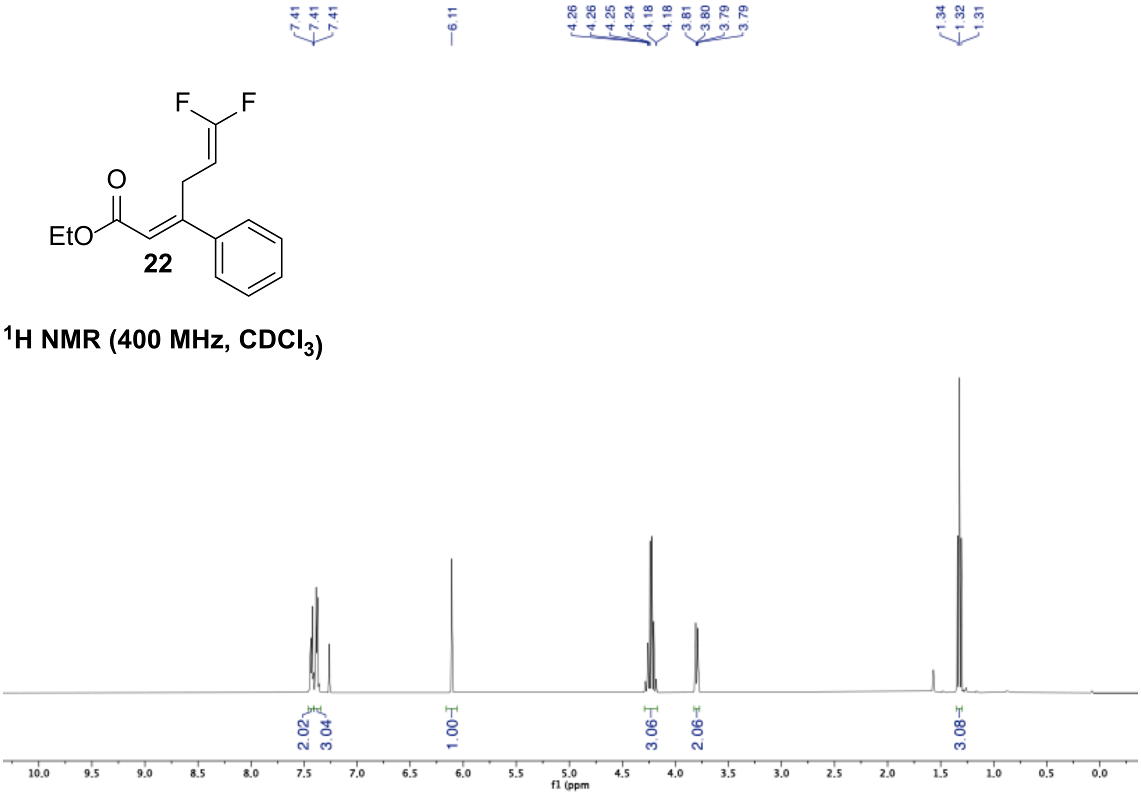

^{1}H NMR (400 MHz, $CDCl_3$)

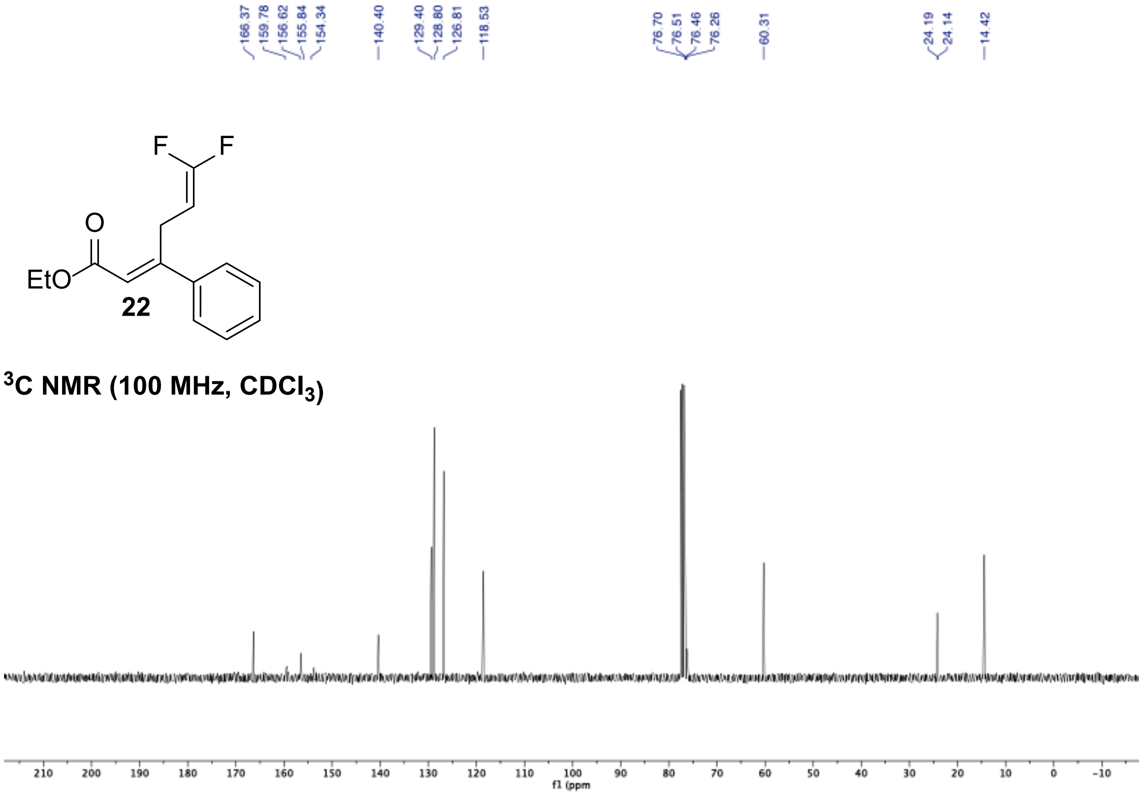

^{13}C NMR (100 MHz, $CDCl_3$)

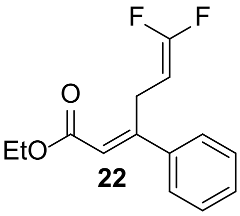

22

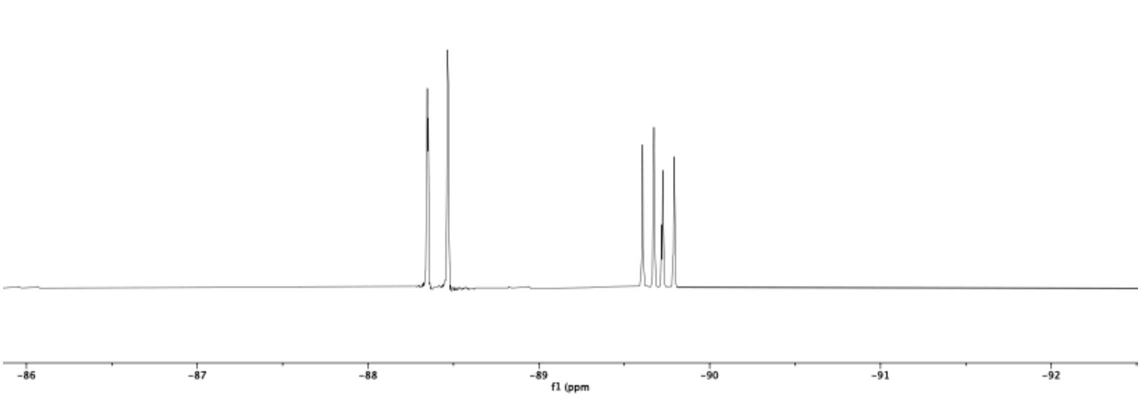

^{19}F NMR (377 MHz, $CDCl_3$)

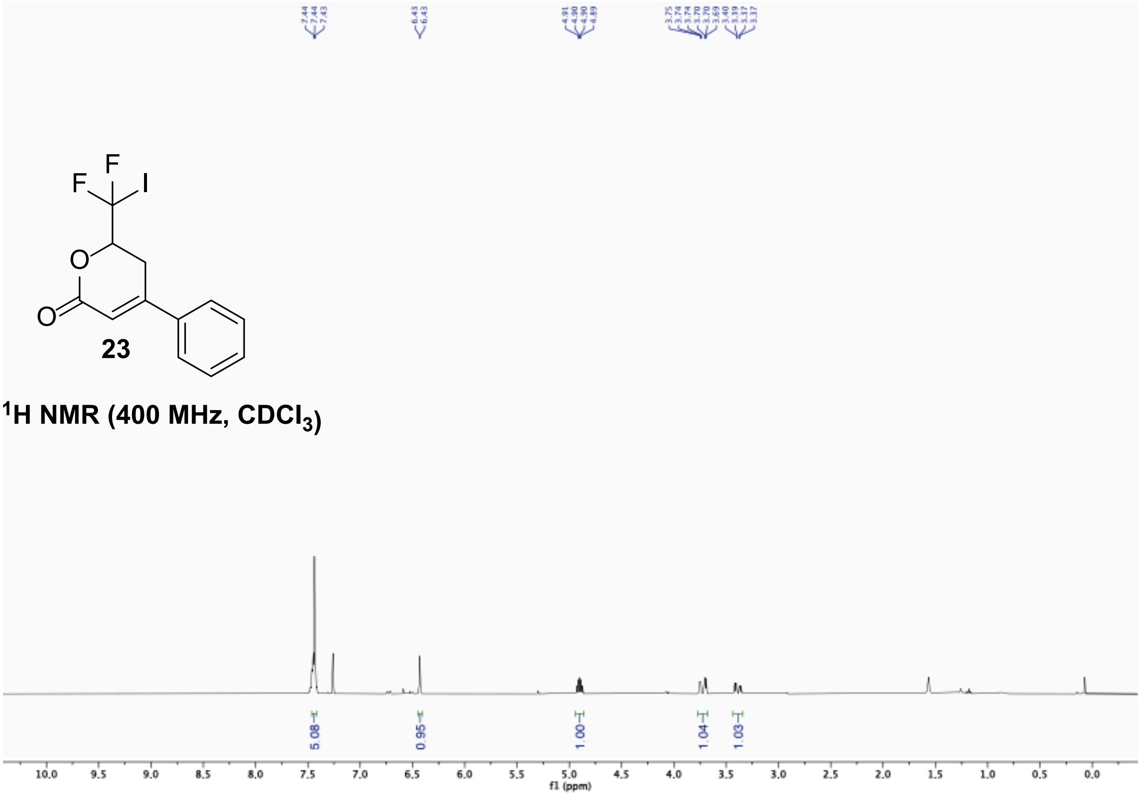

23

^{1}H NMR (400 MHz, $CDCl_3$)

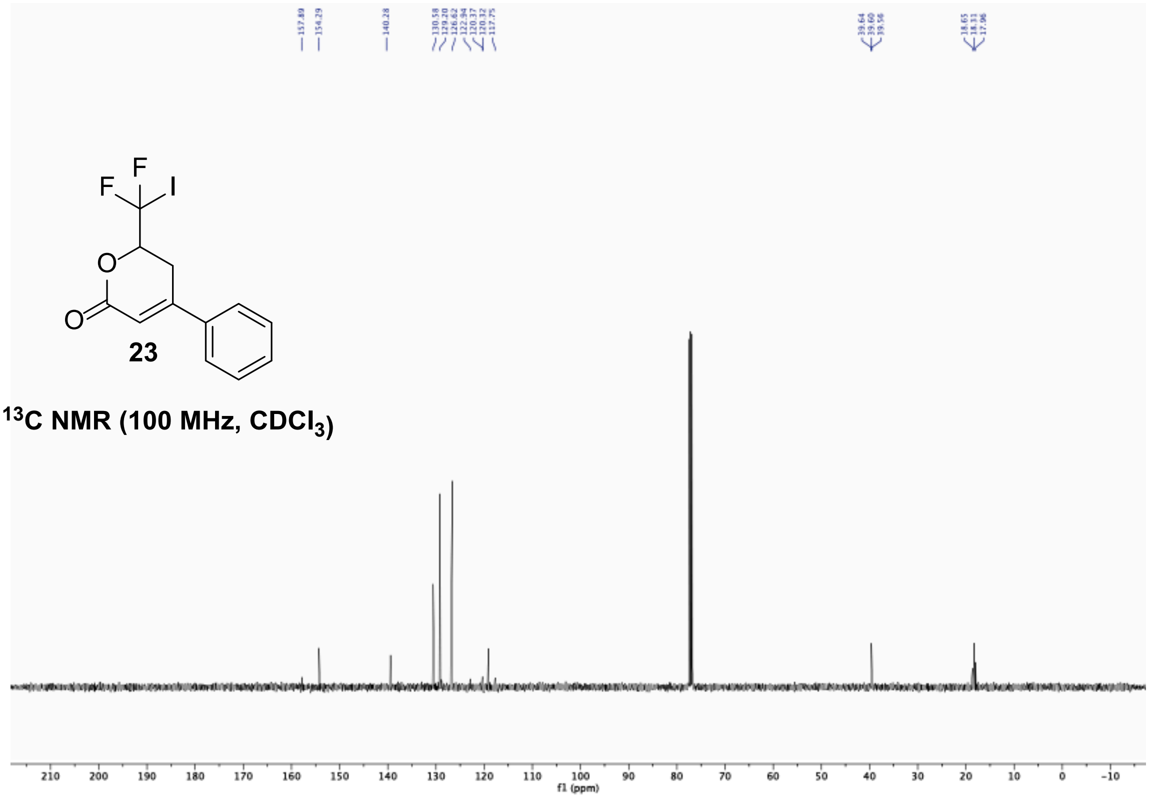

157.89
154.29
140.28
130.58
129.20
126.62
122.94
120.37
120.32
117.75
39.64
39.60
39.56
18.65
18.31
17.98
F
F
I
O
O
23
^{13}C NMR (100 MHz, CDCl$_3$)
f1 (ppm)

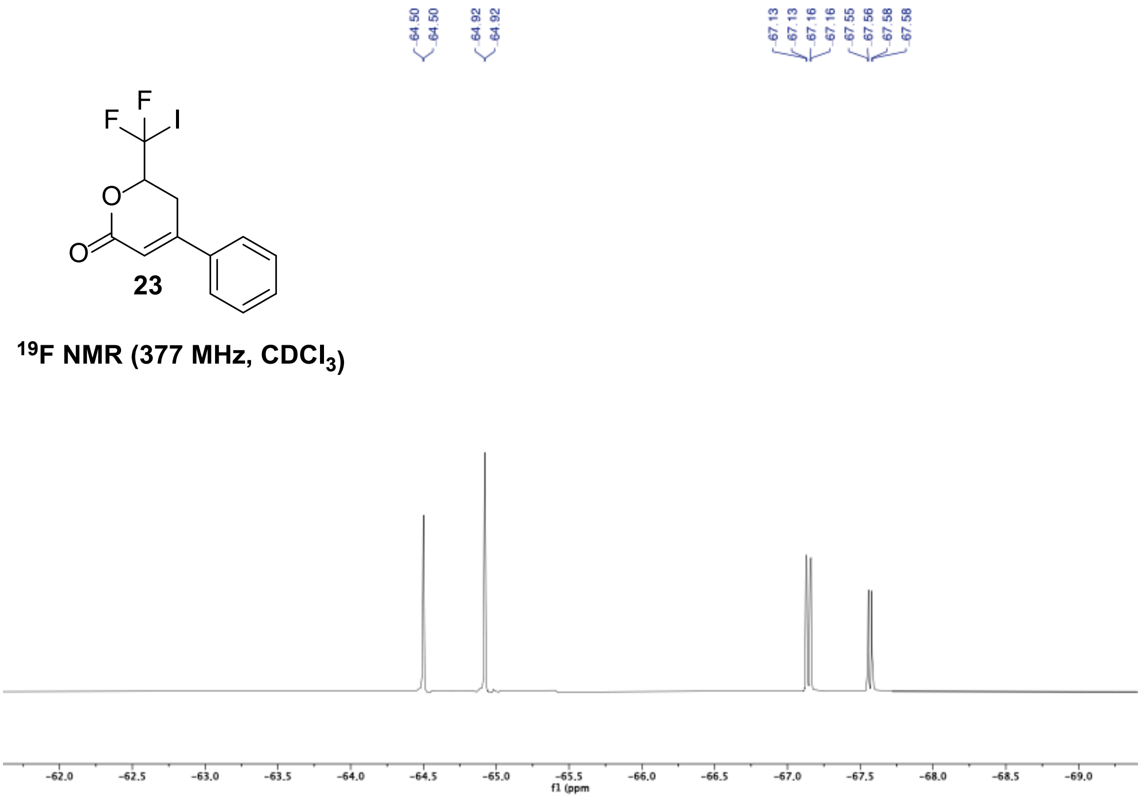

-64.50
-64.50
-64.92
-64.92
-67.13
-67.13
-67.16
-67.16
-67.55
-67.56
-67.58
-67.58
F
F
I
O
O
23
^{19}F NMR (377 MHz, CDCl$_3$)
f1 (ppm

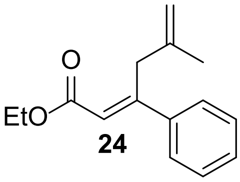

24

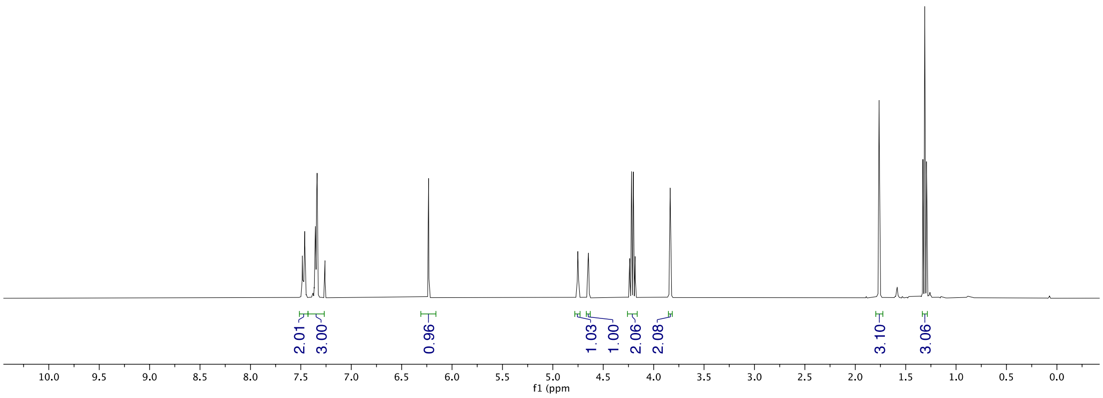

^{1}H NMR (400 MHz, $CDCl_3$)

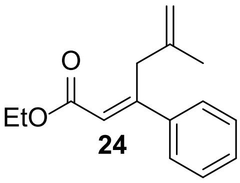

24

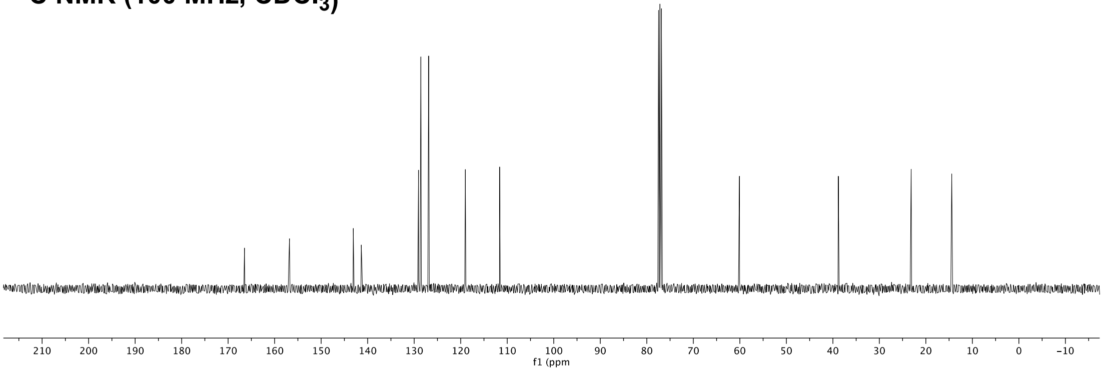

^{13}C NMR (100 MHz, $CDCl_3$)

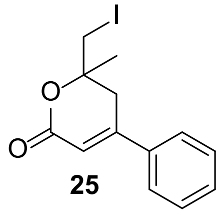

25

^{1}H NMR (400 MHz, $CDCl_3$)

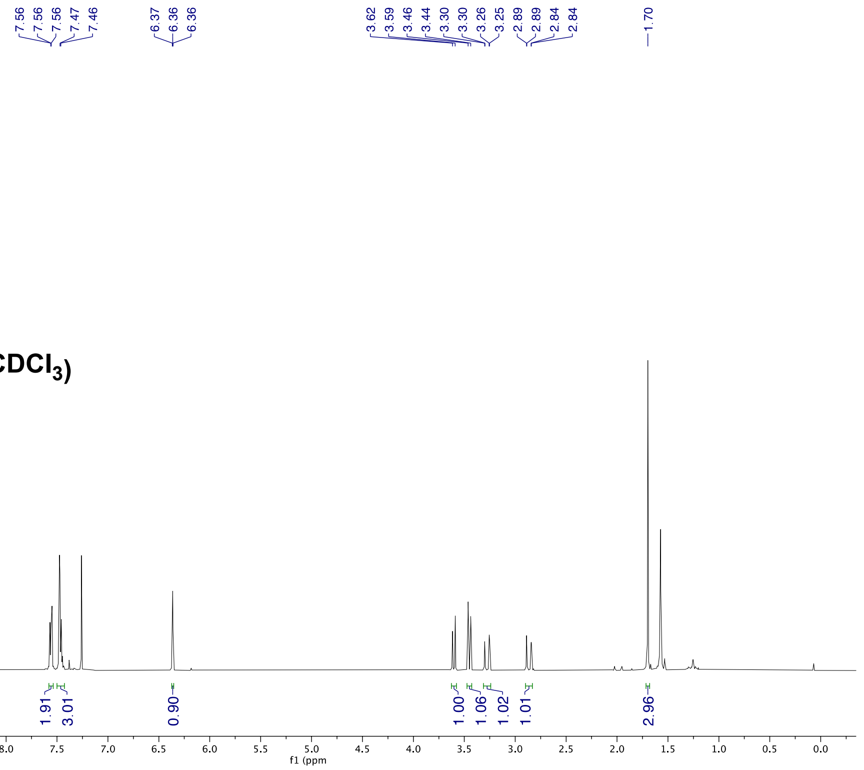

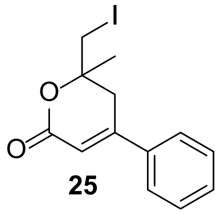

25

^{13}C NMR (100 MHz, $CDCl_3$)

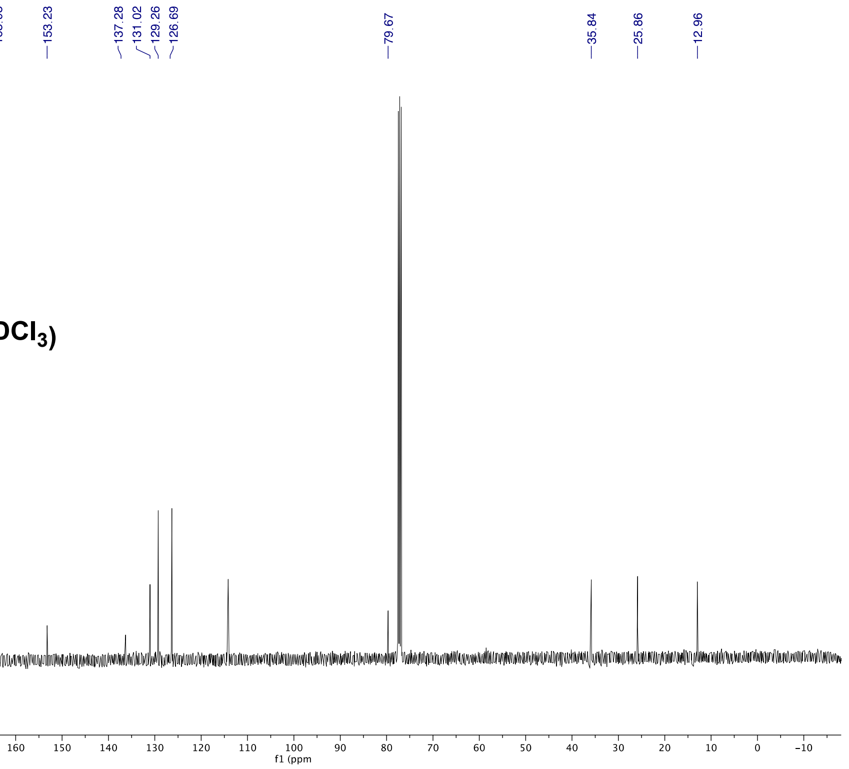

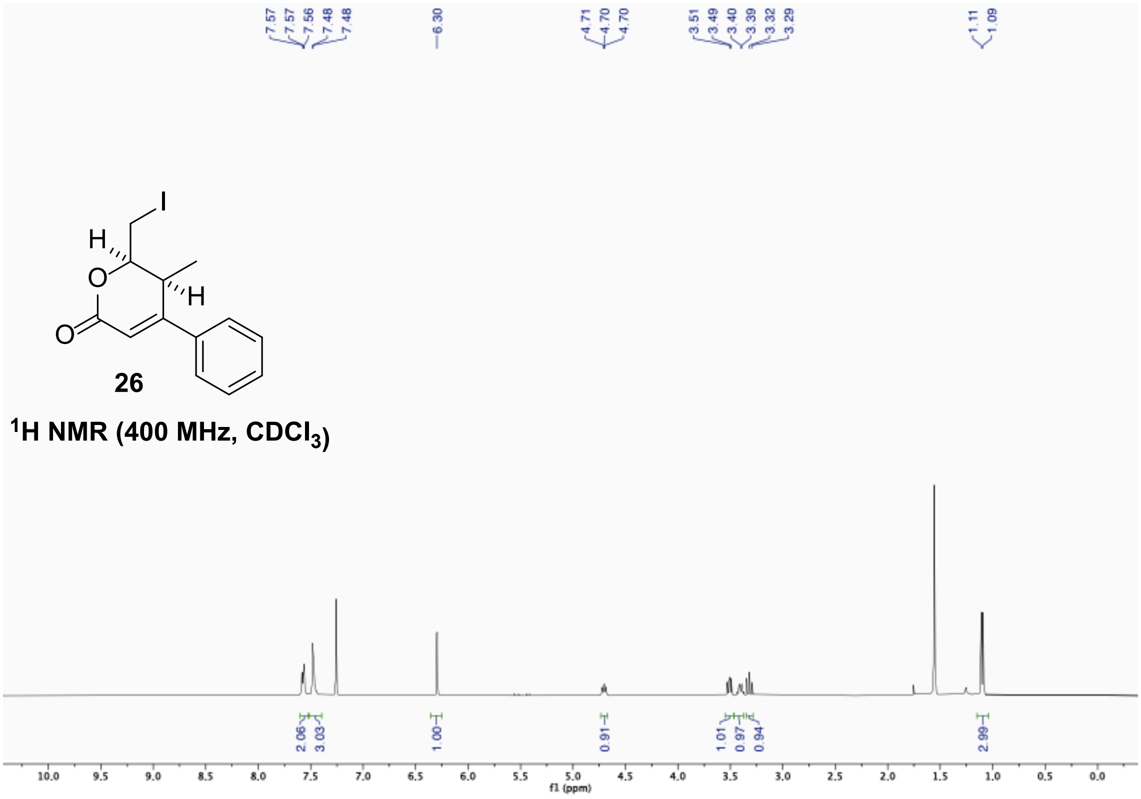
7.57 7.57 7.56 7.48 7.48
6.30
4.71 4.70 4.70
3.51 3.49 3.40 3.39 3.32 3.29
1.11 1.09
26
^{1}H NMR (400 MHz, CDCl$_3$)
2.06 3.03
1.00
0.91
1.01 0.97 0.94
2.99
f1 (ppm)

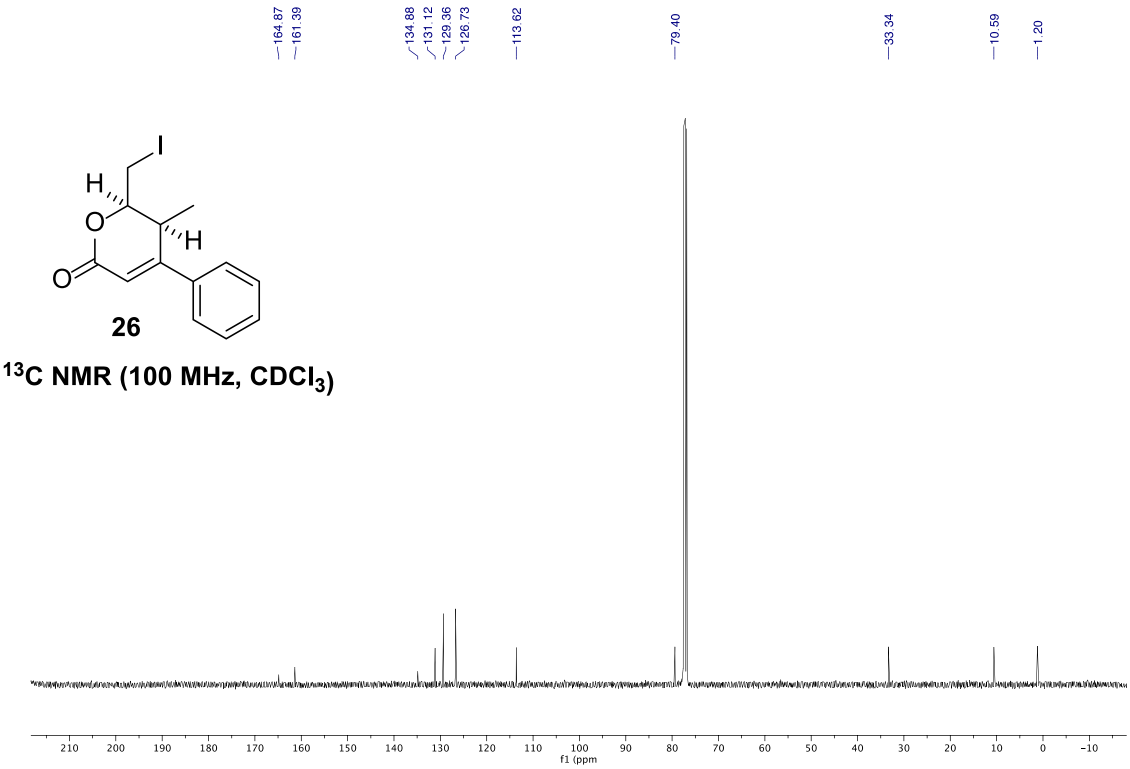
164.87
161.39
134.88
131.12
129.36
126.73
113.62
79.40
33.34
10.59
1.20
26
^{13}C NMR (100 MHz, CDCl$_3$)
f1 (ppm

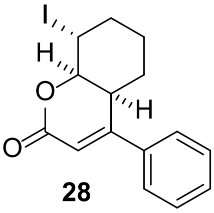

28

^{1}H NMR (400 MHz, $CDCl_3$)

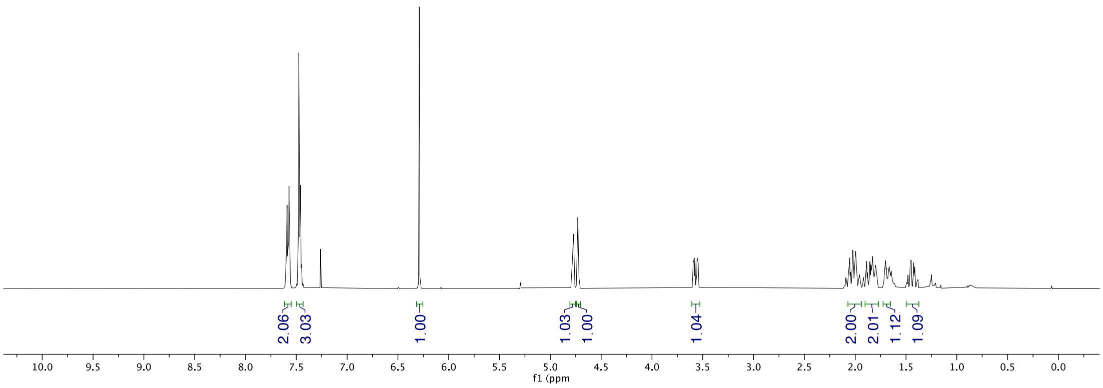

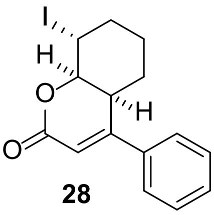

28

^{13}C NMR (100 MHz, $CDCl_3$)

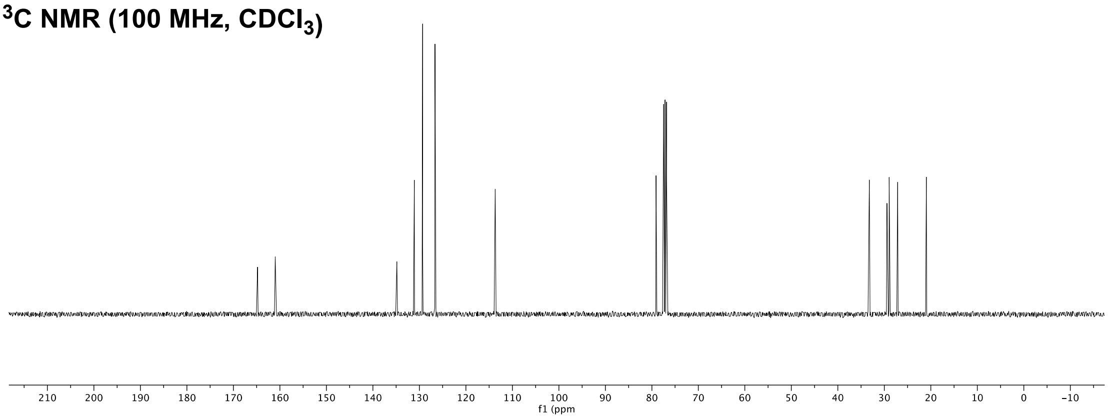

^{1}H, ^{13}C, ^{11}B Spectra for α-pyrone 27

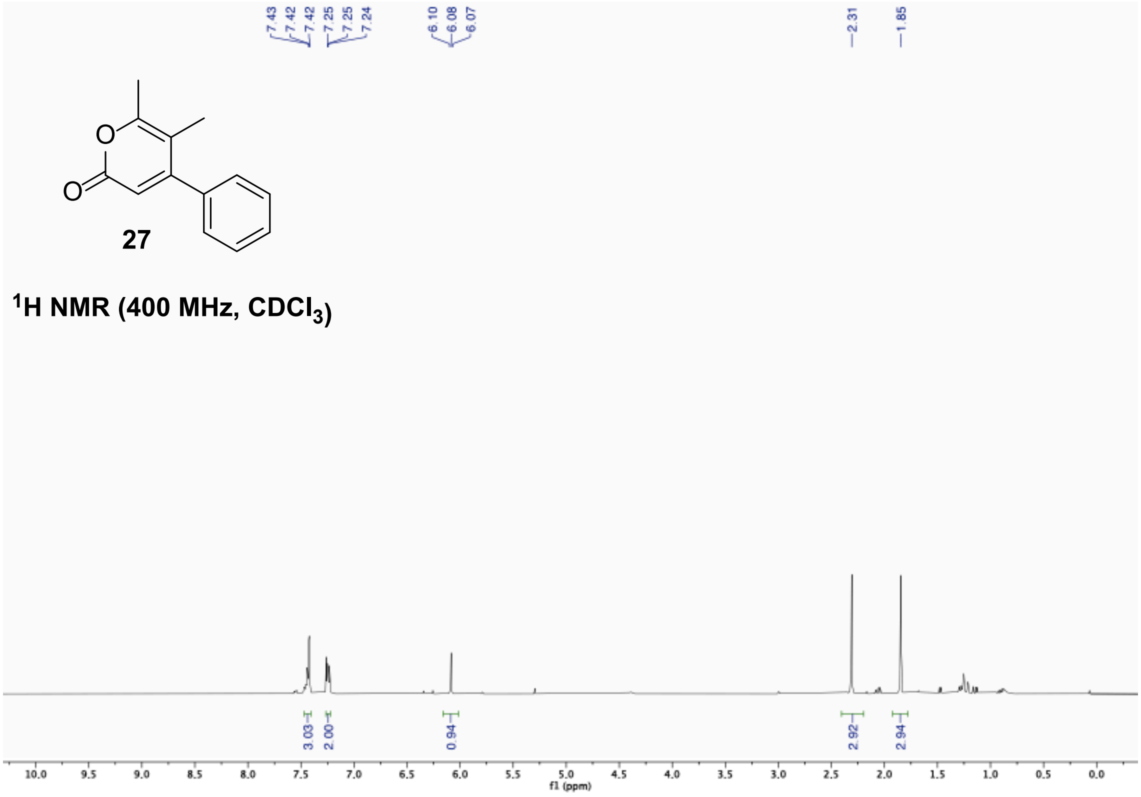

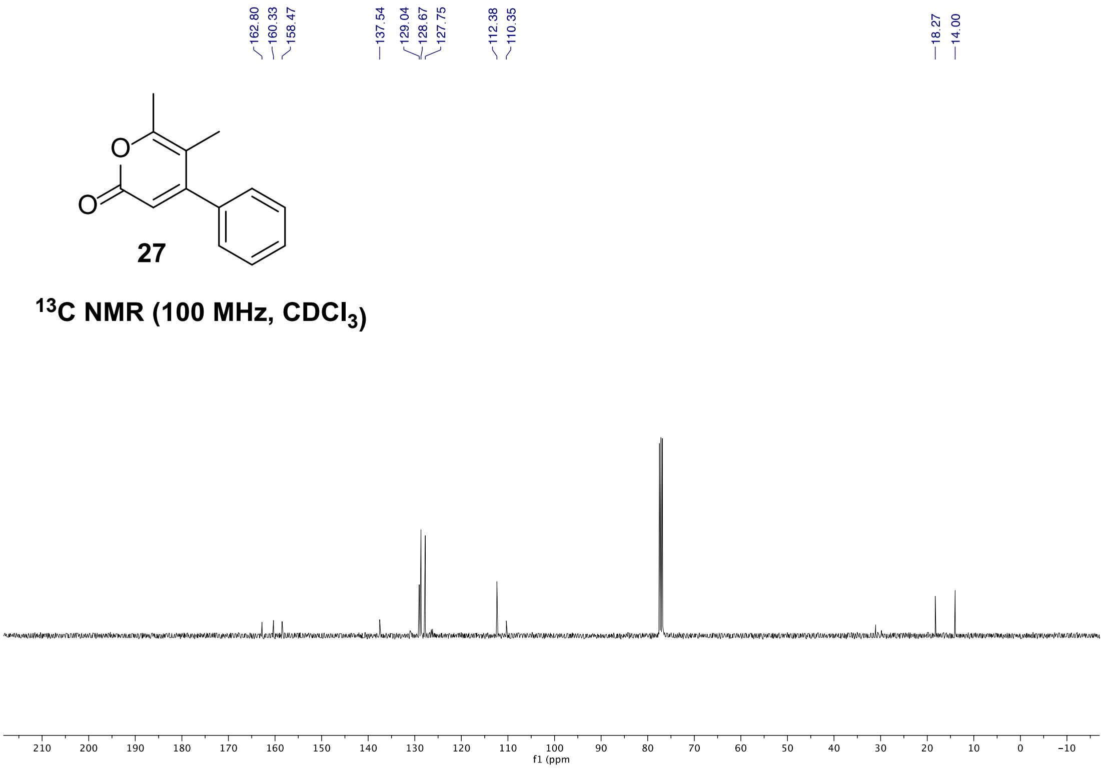

X-Ray single-crystal diffraction analysis for product 21

For Crystal growth, we add the product inside a vial which is positioned at 45º angle. We add inside the vial 1mL hexane and next we slowly add drop by drop 0.3 mL of dichloromethane. We close the vial with a septum and we put a needle inside to let the solvent evaporate at r.t. until we got the crystals.

Data collection: Crystal structure determination was carried out using a Rigaku diffractometer equipped with a Pilatus 200K area detector, a Rigaku MicroMax-007HF microfocus rotating anode with MoK_{α} radiation, Confocal Max Flux optics and an Oxford Cryosystems low temperature device Cryostream 700 plus (T = -173 ˚C). Full-sphere data collection was used with ω and φ scans. *Programs used:* Data collection and reduction with CrysAlisPro V/.60A and absorption correction with Scale3 Abspack scaling algorithm.

Structure Solution and Refinement: Crystal structure solution was achieved using the computer program SHELXT. Visualization was performed with the program SHELXle. Missing atoms were subsequently located from difference Fourier synthesis and added to the atom list. Least-squares refinement on F using all measured intensities was carried out using the program SHELXL 2015. All non-hydrogen atoms were refined including anisotropic displacement parameters.

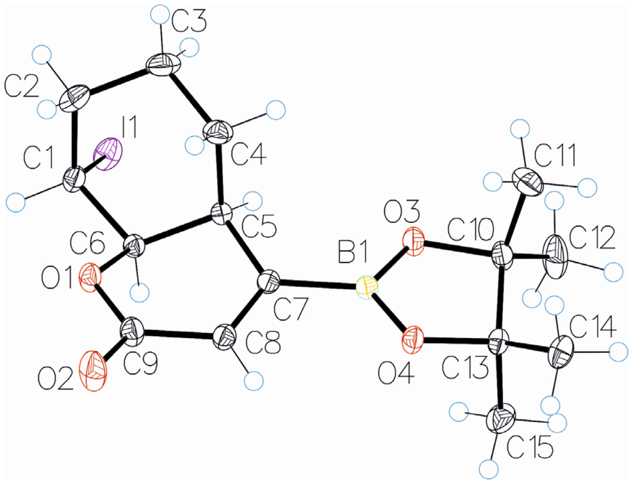

Table 1 Crystal data and structure refinement for MP-490.

Identification code	MP-490
Empirical formula	$C_{15}H_{22}BO_4I$
Formula weight	404.03
Temperature/K	100
Crystal system	monoclinic
Space group	$P2_1/c$
a/Å	9.7788(2)
b/Å	15.9386(2)
c/Å	10.8361(2)
α/°	90
β/°	96.336(2)
γ/°	90
Volume/Å^3	1678.60(5)
Z	4
ρ_{calc}g/cm^3	1.599
μ/mm^{-1}	1.919
F(000)	808.0
Crystal size/mm^3	0.8 × 0.4 × 0.2
Radiation	MoKα (λ = 0.71073)
2Θ range for data collection/°	4.564 to 69.232
Index ranges	$-15 \le h \le 13$, $-25 \le k \le 18$, $-17 \le l \le 16$
Reflections collected	26681
Independent reflections	6708 [R_{int} = 0.0395, R_{sigma} = 0.0707]
Data/restraints/parameters	6708/0/194
Goodness-of-fit on F^2	1.045
Final R indexes [I>=2σ (I)]	R_1 = 0.0223, wR_2 = 0.0563
Final R indexes [all data]	R_1 = 0.0301, wR_2 = 0.0573
Largest diff. peak/hole / e Å^{-3}	1.16/-0.80

Table 2 Fractional Atomic Coordinates (×10^4) and Equivalent Isotropic Displacement Parameters (Å^2×10^3) for MP-490. U_{eq} is defined as 1/3 of the trace of the orthogonalised U_{IJ} tensor.

Atom	*x*	*y*	*z*	U(eq)
I1	1364.2(2)	8073.7(2)	5298.1(2)	22.43(3)
O1	1259.9(9)	5477.5(6)	4269.4(8)	18.60(16)
O2	1072.8(12)	4287.5(7)	3224.5(11)	32.7(2)
O3	5784.4(9)	6734.5(6)	2797.9(9)	18.92(16)
O4	5522.4(9)	5497.8(5)	1716.4(8)	17.17(16)
C1	1273.0(12)	6715.8(8)	5441.0(10)	16.6(2)
C2	2076.1(15)	6407.1(9)	6633.1(11)	23.1(2)
C3	3629.6(15)	6482.0(10)	6627.5(11)	24.9(3)
C4	4094.7(13)	6020.8(9)	5507.4(11)	20.9(2)
C5	3338.5(11)	6347.7(7)	4277.3(10)	13.70(18)

Table 2 Fractional Atomic Coordinates (×10^4) and Equivalent Isotropic Displacement Parameters (Å^2×10^3) for MP-490. U_{eq} is defined as 1/3 of the trace of the orthogonalised U_{IJ} tensor.

Atom	*x*	*y*	*z*	U(eq)
C6	1782.1(11)	6338.2(7)	4280.4(10)	13.79(18)
C7	3713.2(11)	5825.3(7)	3202.2(10)	14.37(18)
C8	2897.8(12)	5179.1(8)	2810.0(11)	16.8(2)
C9	1678.9(13)	4941.9(8)	3417.0(12)	19.4(2)
C10	7024.3(12)	6638.4(8)	2160.9(12)	19.0(2)
C11	8148.2(15)	6315.5(11)	3132.8(14)	31.5(3)
C12	7417.3(17)	7487.9(9)	1673.7(17)	32.8(3)
C13	6558.8(12)	5981.5(7)	1135.2(10)	15.64(19)
C14	7668.8(14)	5383.3(9)	802.5(12)	22.4(2)
C15	5801.3(14)	6363.6(10)	-38.7(12)	25.2(3)
B1	5037.6(13)	6020.2(8)	2566.6(12)	14.8(2)

Table 3 Anisotropic Displacement Parameters (Å^2×10^3) for MP-490. The Anisotropic displacement factor exponent takes the form: -2π^2[h^{2}a*^{2}U$_{11}$+2hka*b*U$_{12}$+…].

Atom	U_{11}	U_{22}	U_{33}	U_{23}	U_{13}	U_{12}
I1	28.51(5)	16.31(5)	24.14(5)	-2.37(3)	10.31(3)	0.75(3)
O1	18.1(4)	16.3(4)	22.7(4)	-3.5(3)	8.0(3)	-5.1(3)
O2	31.9(5)	21.5(5)	47.9(6)	-12.1(4)	19.1(5)	-12.1(4)
O3	16.1(4)	18.7(4)	23.2(4)	-5.2(3)	7.4(3)	-3.4(3)
O4	17.6(4)	16.3(4)	18.7(4)	-1.4(3)	7.3(3)	-3.6(3)
C1	18.0(5)	16.5(5)	16.3(4)	-0.6(4)	6.2(4)	-2.3(4)
C2	32.0(7)	23.9(6)	14.2(5)	1.1(4)	6.0(4)	-0.8(5)
C3	27.7(6)	30.9(7)	14.8(5)	-1.9(5)	-3.7(4)	2.1(5)
C4	19.3(5)	25.6(6)	16.8(5)	-0.7(4)	-2.5(4)	2.9(4)
C5	12.3(4)	15.4(5)	13.5(4)	0.1(4)	1.8(3)	-1.0(4)
C6	13.7(4)	13.8(5)	14.1(4)	-0.8(3)	2.7(3)	-1.7(4)
C7	13.7(4)	15.8(5)	13.8(4)	1.4(4)	2.1(3)	1.3(4)
C8	16.5(5)	17.2(5)	17.2(5)	-1.7(4)	4.4(4)	-0.9(4)
C9	17.7(5)	17.8(5)	23.6(5)	-2.9(4)	6.1(4)	-2.8(4)
C10	14.0(5)	20.3(5)	23.6(5)	-3.2(4)	5.9(4)	-3.0(4)
C11	17.7(6)	46.7(9)	28.8(7)	-8.3(6)	-2.7(5)	-1.6(6)
C12	29.7(7)	21.1(6)	51.0(9)	-3.7(6)	20.4(6)	-8.8(5)
C13	14.9(5)	16.6(5)	16.1(4)	1.8(4)	4.7(4)	-1.0(4)
C14	22.4(6)	22.9(6)	23.4(5)	0.8(5)	9.8(4)	4.2(5)
C15	23.9(6)	32.9(7)	19.0(5)	6.2(5)	3.7(5)	4.7(5)
B1	13.2(5)	16.3(5)	15.0(5)	0.5(4)	1.9(4)	-0.2(4)

Table 4 Bond Lengths for MP-490.

Atom	Atom	Length/Å	Atom	Atom	Length/Å
I1	C1	2.1723(13)	C4	C5	1.5419(16)
O1	C6	1.4634(14)	C5	C6	1.5224(15)
O1	C9	1.3541(15)	C5	C7	1.5097(15)
O2	C9	1.2064(16)	C7	C8	1.3425(16)
O3	C10	1.4680(14)	C7	B1	1.5636(16)
O3	B1	1.3608(16)	C8	C9	1.4729(16)
O4	C13	1.4694(14)	C10	C11	1.525(2)
O4	B1	1.3649(15)	C10	C12	1.5180(19)
C1	C2	1.5178(18)	C10	C13	1.5579(17)
C1	C6	1.5264(16)	C13	C14	1.5178(17)
C2	C3	1.525(2)	C13	C15	1.5263(17)
C3	C4	1.5305(18)			

Table 5 Bond Angles for MP-490.

Atom	Atom	Atom	Angle/°	Atom	Atom	Atom	Angle/°
C9	O1	C6	117.71(9)	O1	C9	C8	117.61(10)
B1	O3	C10	106.28(9)	O2	C9	O1	118.89(11)
B1	O4	C13	106.06(9)	O2	C9	C8	123.44(11)
C2	C1	I1	111.23(8)	O3	C10	C11	106.27(10)
C2	C1	C6	112.84(10)	O3	C10	C12	108.93(11)
C6	C1	I1	108.32(7)	O3	C10	C13	102.37(9)
C1	C2	C3	113.17(10)	C11	C10	C13	113.42(11)
C2	C3	C4	110.43(11)	C12	C10	C11	110.50(12)
C3	C4	C5	111.54(11)	C12	C10	C13	114.62(11)
C6	C5	C4	112.27(9)	O4	C13	C10	101.99(8)
C7	C5	C4	110.28(10)	O4	C13	C14	108.50(10)
C7	C5	C6	108.91(9)	O4	C13	C15	105.80(10)
O1	C6	C1	103.36(9)	C14	C13	C10	115.55(10)
O1	C6	C5	110.94(9)	C14	C13	C15	110.29(10)
C5	C6	C1	114.52(9)	C15	C13	C10	113.78(11)
C5	C7	B1	120.91(10)	O3	B1	O4	114.54(10)
C8	C7	C5	118.76(10)	O3	B1	C7	122.60(10)
C8	C7	B1	120.32(10)	O4	B1	C7	122.85(11)
C7	C8	C9	122.49(11)				

Table 6 Torsion Angles for MP-490.

A	B	C	D	Angle/°	A	B	C	D	Angle/°
I1	C1	C2	C3	-71.67(12)	C7	C5	C6	C1	170.06(10)
I1	C1	C6	O1	-161.87(7)	C7	C8	C9	O1	10.53(18)

Table 6 Torsion Angles for MP-490.

A	B	C	D	Angle/°	A	B	C	D	Angle/°
I1	C1	C6	C5	77.33(10)	C7	C8	C9	O2	-166.81(14)
O3	C10	C13	O4	-28.97(11)	C8	C7	B1	O3	169.94(12)
O3	C10	C13	C14	-146.43(10)	C8	C7	B1	O4	-8.63(18)
O3	C10	C13	C15	84.47(12)	C9	O1	C6	C1	-172.49(10)
C1	C2	C3	C4	-55.70(16)	C9	O1	C6	C5	-49.29(13)
C2	C1	C6	O1	74.52(12)	C10	O3	B1	O4	-8.34(14)
C2	C1	C6	C5	-46.28(14)	C10	O3	B1	C7	172.98(11)
C2	C3	C4	C5	56.48(15)	C11	C10	C13	O4	85.08(12)
C3	C4	C5	C6	-52.77(14)	C11	C10	C13	C14	-32.39(14)
C3	C4	C5	C7	-174.41(10)	C11	C10	C13	C15	-161.48(11)
C4	C5	C6	O1	-68.88(12)	C12	C10	C13	O4	-146.73(11)
C4	C5	C6	C1	47.64(13)	C12	C10	C13	C14	95.80(13)
C4	C5	C7	C8	94.12(13)	C12	C10	C13	C15	-33.29(15)
C4	C5	C7	B1	-84.89(13)	C13	O4	B1	O3	-11.58(14)
C5	C7	C8	C9	-2.23(18)	C13	O4	B1	C7	167.10(10)
C5	C7	B1	O3	-11.07(17)	B1	O3	C10	C11	-96.08(12)
C5	C7	B1	O4	170.36(11)	B1	O3	C10	C12	144.86(12)
C6	O1	C9	O2	-165.84(12)	B1	O3	C10	C13	23.12(12)
C6	O1	C9	C8	16.70(16)	B1	O4	C13	C10	24.84(12)
C6	C1	C2	C3	50.32(15)	B1	O4	C13	C14	147.25(10)
C6	C5	C7	C8	-29.49(14)	B1	O4	C13	C15	-94.40(11)
C6	C5	C7	B1	151.50(10)	B1	C7	C8	C9	176.78(11)
C7	C5	C6	O1	53.54(11)					

Table 7 Hydrogen Atom Coordinates (Å×10^4) and Isotropic Displacement Parameters (Å^2×10^3) for MP-490.

Atom	*x*	*y*	*z*	U(eq)
H1	288.26	6550.56	5454.73	20
H2A	1839.05	5812.14	6763.44	28
H2B	1793.9	6734.42	7339.05	28
H3A	4098.88	6239.58	7402.57	30
H3B	3888.78	7081.32	6594.33	30
H4A	3913.38	5413.1	5585.01	25
H4B	5098.21	6097.33	5497.87	25
H5	3636.69	6938.96	4152.46	16
H6	1346.11	6641.96	3530.54	17
H8	3115.29	4862.82	2114.63	20

Table 7 Hydrogen Atom Coordinates (Å×10⁴) and Isotropic Displacement Parameters (Å²×10³) for MP-490.

Atom	*x*	*y*	*z*	U(eq)
H11A	9008.6	6252.49	2756.11	47
H11B	7873.75	5770.79	3444.99	47
H11C	8284.41	6715.93	3821.76	47
H12A	8185.5	7420.16	1172.95	49
H12B	7693.32	7862.78	2373.88	49
H12C	6626.78	7729.01	1159.82	49
H14A	7261.72	4966.4	206.49	34
H14B	8081.15	5098.15	1554.34	34
H14C	8380.3	5699.13	431.88	34
H15A	6448.47	6690.87	-473.54	38
H15B	5064.45	6730.9	186.55	38
H15C	5404.74	5914.3	-582.85	38

Crystal structure determination of [MP-490]

Crystal Data for $C_{15}H_{22}BO_4I$ (M =404.03 g/mol): monoclinic, space group $P2_1/c$ (no. 14), a = 9.7788(2) Å, b = 15.9386(2) Å, c = 10.8361(2) Å, β = 96.336(2)°, V = 1678.60(5) Å^3, Z = 4, T = 100 K, μ(MoKα) = 1.919 mm^{-1}, $Dcalc$ = 1.599 g/cm^3, 26681 reflections measured (4.564° ≤ 2Θ ≤ 69.232°), 6708 unique (R_{int} = 0.0395, R_{sigma} = 0.0707) which were used in all calculations. The final R_1 was 0.0223 (I > 2σ(I)) and wR_2 was 0.0573 (all data).

Refinement model description

Number of restraints - 0, number of constraints - unknown.

Details:

```
1. Fixed Uiso
 At 1.2 times of:
  All C(H) groups, All C(H,H) groups
 At 1.5 times of:
  All C(H,H,H) groups
2.a Ternary CH refined with riding coordinates:
 C1(H1), C5(H5), C6(H6)
2.b Secondary CH2 refined with riding coordinates:
 C2(H2A,H2B), C3(H3A,H3B), C4(H4A,H4B)
2.c Aromatic/amide H refined with riding coordinates:
 C8(H8)
2.d Idealised Me refined as rotating group:
 C11(H11A,H11B,H11C), C12(H12A,H12B,H12C), C14(H14A,H14B,H14C), C15(H15A,H15B,
 H15C)
```

This report has been created with Olex2, compiled on 2022.04.12 svn.rca3783a0 for Rigaku Oxford Diffraction.

References

1. Doan, S. H.; Ton, N. N.; Mai, B. K.; Nguyen, T. V. Organosuperbase-Catalyzed 1,1-Diboration of Alkynes, *ACS Catalysis* **2022**, *12*, 12409–12418.

2. Luo, T.; Dai, M.; Zheng, Sh.-L.;Schreiber, S. L. Syntheses of α-Pyrones Using Gold-Catalyzed Coupling Reactions, *Org. Lett.* **2011**, *13*, 2834-2836
